# Syntheses of Thailandepsin B Pseudo‐Natural Products: Access to New Highly Potent HDAC Inhibitors via Late‐Stage Modification

**DOI:** 10.1002/chem.202002449

**Published:** 2020-11-03

**Authors:** Jana Brosowsky, Monika Lutterbeck, Amelie Liebich, Manfred Keller, Daniel Herp, Anja Vogelmann, Manfred Jung, Bernhard Breit

**Affiliations:** ^1^ Institut für Organische Chemie Albert-Ludwigs-Universität Freiburg Albertstr. 21 79104 Freiburg Germany; ^2^ Institut für Pharmazeutische Wissenschaften Albert-Ludwigs-Universität Freiburg Albertstr. 25 79104 Freiburg Germany

**Keywords:** asymmetric catalysis, histone deacetylase inhibitors, rhodium, synthetic methods, thailandepsin

## Abstract

New Thailandepsin B pseudo‐natural products have been prepared. Our synthetic strategy offers the possibility to introduce varying warheads via late stage modification. Additionally, it gives access to the asymmetric branched allylic ester moiety of the natural product in a highly diastereoselective manner applying rhodium‐catalyzed hydrooxycarbonylation. The newly developed pseudo‐natural products are extremely potent and selective HDAC inhibitors. The non‐proteinogenic amino acid d‐norleucine was obtained enantioselectively by a recently developed method of rhodium‐catalyzed hydroamination.

## Graphical abstract

**New thailandepsin B** pseudo‐natural products have been prepared applying rhodium‐catalyzed hydrooxycarbonylation. This strategy gives access to the asymmetric branched allylic ester moiety in a highly diastereoselective manner. Varying warheads have been introduced via late stage modification. The newly developed compounds are extremely potent and selective HDAC inhibitors

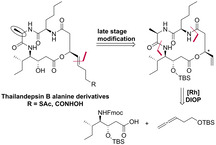

Thailandepsins are a family of natural products which had been isolated from a culture broth of the gram‐negative bacteria *Burkholderia Thailandensis* E264. In 2011, Cheng et al. identified thailandepsins A (**1**) and B (**2**) through systematic overexpression of transcription factors.^[1]^ The isolation of thailandepsins C–F was reported by the same group in the following year (Figure 1).^[2]^ At the same time, Brady et al. independently observed the natural products burkholdac A (**3**) and B (**1**) from the same bacterial strain and gene clusters.^[3]^ They were found to be identical to thailandepsins C (**3**) and A (**1**). Thailandepsins show nanomolar inhibitory activity of histone deacetylases (HDACs) comparable to the approved drug FK228 (romidepsin, **7**)^[4]^ with even enhanced isoform selectivity. HDAC1 (class I) is inhibited with IC_50_ values in a low nanomolar range whereas HDAC4 (class IIa) and HDAC6 (class IIb) are only inhibited in micromolar concentrations.^[2]^ Besides being approved as anticancer agents, inhibitors of zinc‐dependent HDACs are promising for the treatment of further diseases like inflammation, neurodegeneration or metabolic syndrome.^[5]^

**Figure 1:**
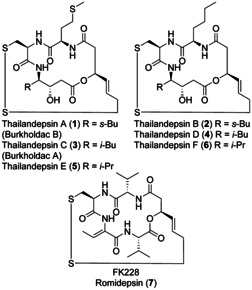
Thailandepsins A‐F and FK228.

For FK228, the disulfide prodrug is reduced intracellularly to the zinc‐binding thiol as enzyme inactivating group.^[6]^ An identical warhead is present in other depsipeptide natural product HDAC inhibitors, for example, in spiruchostatins.^[7]^ Like thailandepsins, they exhibit a statine unit (a β‐hydroxy‐γ‐amino acid) in their macrocyclic backbone. Largazole^[8]^ contains the thiol as a thioester prodrug which is hydrolyzed intracellularly to give the free thiol.

For thailandepsins, the same mechanism of action like in FK228 is assumed; the disulfide bridge can be reduced to the free thiol which acts as the zinc‐binding functional group (Scheme 1).^[1a]^ In accordance with a general structure model for HDAC inhibitors proposed by Jung et al.,^[9]^ thailandepsins contain a macrocyclic backbone as cap group which is suggested to be responsible for enzyme specificity. The cap group is connected by an alkyl chain as spacer to the actual enzyme inhibiting warhead. Thailandepsins A and C have been synthesized by Ganesan et al.,^[10]^ Xu and Ye et al.,^[11]^ and Katoh et al.^[12]^ Total syntheses of thailandepsin B have been reported by Cheng et al.^[13]^ and Katoh et al.^[14]^ The same group published the syntheses of Thailandepsins d–F.^[15]^

**Scheme 1:**
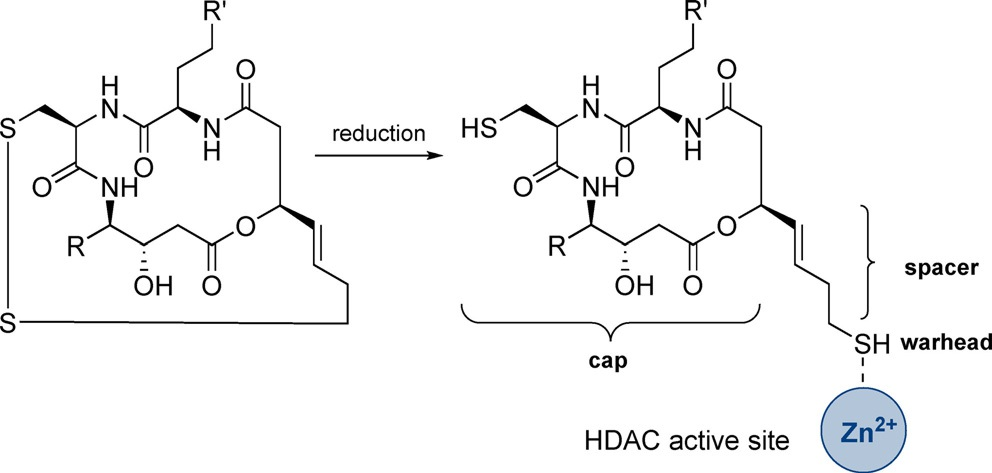
Reduction of the disulfide in thailandepsins to generate the thiol as zinc binding warhead.

In this work, we envisioned the syntheses of thailandepsin B pseudo‐natural products with varying warheads, for example, a hydroxamic acid (compound **8**, Scheme 2). This functionality has shown to be a highly potent zinc‐binding group. It is present in already approved drugs like SAHA.^[16]^ For cyclic tetrapeptide HDAC inhibitors, hydroxamic acid warheads have successfully been introduced. These compounds inhibited HDAC1 at low nanomolar concentrations.^[17]^ Our syntheses are based on the macrocyclic precursor **9** which enables the introduction of varying warheads on late stage via cross metathesis.

**Scheme 2:**
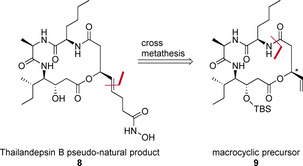
Thailandepsin B pseudo‐natural product **8** and retrosynthetic approach: Macrocyclic precursor **9** offers the possibility of late stage modification to introduce varying warheads via cross‐metathesis. Disconnection of **8** via macrolactamization.

The chiral allylic ester in **9** was targeted to be obtained by the recently developed atom economic and redox‐neutral rhodium‐catalyzed addition of a carboxylic acid to an allene.^[18]^ This method gives access to branched allylic esters in a highly enantio‐ or diastereoselective fashion. In preceding syntheses of the thailandepsin natural products, the highlighted stereocenter was obtained by stoichiometric amounts of a chiral auxiliary,[^10^, ^11^, ^12^] use of l‐malic acid^[19]^ or the separation of two diastereomers derived from a racemate.[^15^, ^20^] d‐cysteine in the cyclic backbone is exchanged to d‐alanine.^[21]^ Retrosynthetically, compound **9** is obtained by macrolactamization (Scheme 2). Next, the carboxylic acid **10** is yielded by deprotection of the primary silyl ether in **11** followed by oxidation of the alcohol (Scheme 3).

**Scheme 3:**
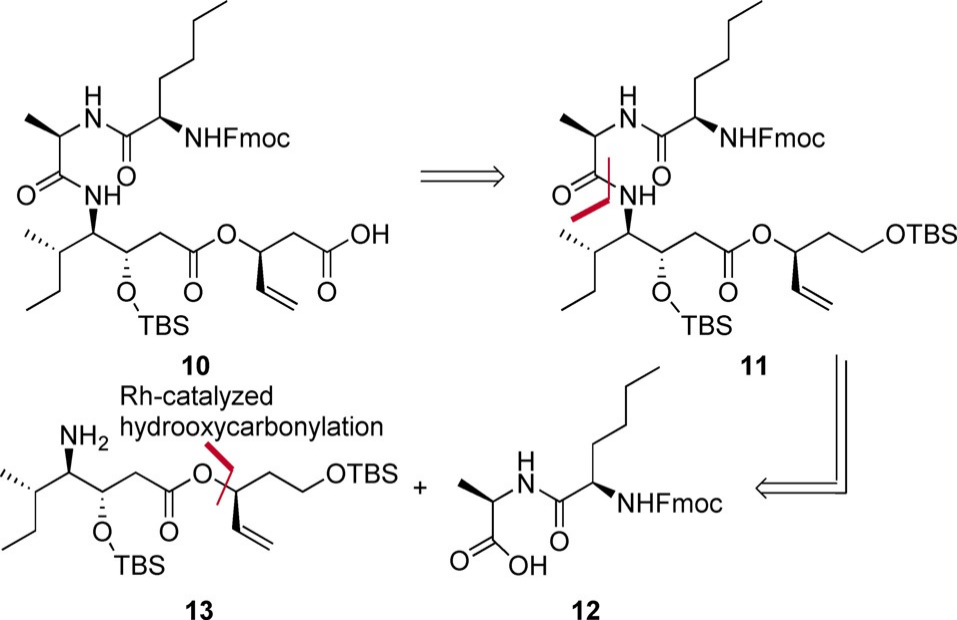
Retrosynthetic analysis.

**11** is built up from a dipeptide (**12**) containing d‐alanine and N‐protected d‐norleucine and compound **13** with the chiral allylic ester which is obtained by rhodium‐catalyzed hydrooxycarbonylation (Scheme 3). d‐Norleucine is a non‐proteinogenic amino acid. As an alternative to commercially available d‐norleucine, we show an elegant pathway applying the recently developed method of enantioselective catalytic hydroamination (Scheme 4) for its synthesis.^[22]^ Benzophenonimine (**14**) is added to hepta‐1,2‐diene (**15**) using [Rh(COD)Cl]_2_, a Josiphos‐ligand and PPTS as an additive. The adduct **16** is directly hydrolyzed by aq. HCl followed by introduction of the protecting group Fmoc (**17**) or Cbz (**18**). For **17**, an excellent enantiomeric excess of 96 % *ee* was determined.

**Scheme 4:**
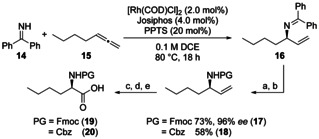
Synthesis of Fmoc‐protected d‐norleucine via rhodium‐catalyzed hydroamination. a) aq. HCl, Et_2_O, r.t., 24 h; b) PGCl, Na_2_CO_3_, dioxane, 0 °C to r.t., 17 h; c) K_2_OsO_2_(OH)_4_, NMO, acetone, H_2_O, THF, 18 h, PG=Fmoc 79 %, PG=Cbz 91 %; d) NaIO_4_, THF/H_2_O, 2 h; e) NaClO_2_, NaH_2_PO_4_, *t*BuOH/H_2_O, PG=Fmoc 1.5 h 70 %, PG=Cbz 20 h, 82 %. NMO=*N*‐Methylmorpholine *N*‐oxide.

Dihydroxylation of the double bond followed by oxidative cleavage delivered the protected d‐amino acids **19** and **20**. The high enantiomeric excess remained unchanged during these transformations. Coupling of **19** to the *tert*‐butyl ester of d‐alanine (**21**) using EDCI*HCl and HOBt followed by cleavage of the *tert*‐butyl ester under acidic conditions with TFA and triethylsilane afforded dipeptide **12** (Scheme 5).

**Scheme 5:**
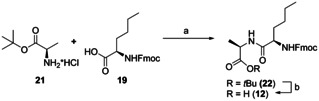
Synthesis of compound **12**. a) EDCI*HCl, HOBt, NEt_3_, DCM, r.t., 18 h, 76 %; b) TFA, Et_3_SiH, DCM, r.t., 18 h, 81 %. EDCI=1‐Ethyl‐3‐(3‐dimethylaminopropyl)carbodiimide, TFA=trifluoroacetic acid.

The known statine^[23]^ **23** was silylated with a TBS group at its secondary alcohol to afford **24**. The chiral allylic ester was built up by addition of **24** to allene **25**. Selected results of the optimization for the rhodium‐catalyzed hydrooxycarbonylation are shown in Table 1. [Rh(COD)Cl]_2_ and DIOP were used as catalyst system.^[18a]^ High diastereomeric ratios were achieved with a temperature of 10 °C and a solution in DCE with a concentration of 0.1 M. Increasing the catalyst loading from 2.5 mol % of [Rh(COD)Cl]_2_ and 5.0 mol % of (*R*,*R*)‐DIOP to 4.5 mol %/9.0 mol % at a reaction time of 48 h gave a high yield of 80 % and a very good d.r. of 88:12. Higher catalyst loadings or longer reaction times increased the yield even more, but let the diastereoselectivity drop. Use of (*S*,*S*)‐DIOP delivered the other diastereomer in an excellent ratio of 5:95. Apparently, here a matched case of substrate and catalyst control is present. The configuration of the newly constructed stereocenter of the allylic ester in **26** was confirmed by the Mosher ester method.^[24]^ For use of (*R*,*R*)‐DIOP, the ester in **26** was hydrolyzed and the obtained chiral alcohol was esterified with both the (*R*)‐ and the (*S*)‐Mosher's acid. Comparison of the ^1^H NMR spectra of both diastereomers of the Mosher esters led to the conclusion that the newly formed chiral center shows the desired (*S*) configuration^[25]^ as expected from previous results.^[18a]^

**Table 1:** Optimization of rhodium‐catalyzed coupling of statine **24** to allene **25**.

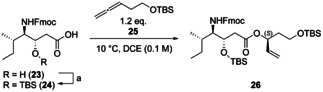				
Entry	Time [h]	[Rh(COD)Cl]_2_/ DIOP	Yield [%]^[b]^	d.r.^[c]^
**1**	48	2.5/ 5.0 mol % (*R*,*R*)	45	82:18
**2**	48	4.5/ 9.0 mol % (*R*,*R*)	80	88:12
**3**	48	7.5/ 15 mol % (*R*,*R*)	98	80:20
**4**	96	4.5/ 9.0 mol % (*R*,*R*)	96	84:16
**5**	48	4.5/ 9.0 mol % (*S*,*S*)	71	5:95

[a] TBSCl, imidazole, DMAP, r.t., 27 h, 54 %; [b] Isolated combined yield of diastereomers; [c] d.r. determined from ^1^H NMR.

The product of the rhodium‐catalyzed hydrooxycarbonylation underwent Fmoc‐deprotection with diethylamine in 5 min (Scheme 6). **13** was used in the coupling to dipeptide **12** with HATU and HOBt without further purification. The primary alcohol was deprotected selectively with HF*pyridine within 2 h. **27** was obtained in a yield of 58 % over three steps starting from **26**. The oxidation to carboxylic acid **10** turned out to be challenging. Commonly used two‐step‐methods via the aldehyde using for example, DMP followed by Pinnick oxidation were unsuccessful. Only a fast procedure using Jones reagent (CrO_3_ in H_2_SO_4_) delivered the desired acid in a reasonable yield. Under these conditions, oxidation takes place very fast which avoids the formation of significant amounts of side products. A quick basic work‐up is required to save the secondary TBS‐group. Fmoc‐deprotection was performed by piperidine. In situ macrolactamization to **9** with peptide coupling reagents was not possible. Purification of the amino acid by reversed phase column chromatography was necessary. Otherwise, the presence of piperidine adducts avoided the formation of the macrocycle. Macrolactamization was achieved using HATU and Hünig's base under high dilution conditions.

**Scheme 6:**
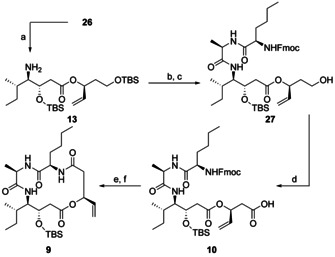
Synthesis of macrocyclic precursor **8**. a) HNEt_2_ (10 %), DMF, r.t., 5 min; b) **12**, HATU, HOBt, DIPEA, DCM, r.t., 24 h; c) HF*pyridine (70 %), pyridine, THF, 0 °C to r.t., 2 h, 58 % over 3 steps; d) Jones reagent, acetone, r.t., 55 sec, 60 %; e) piperidine, DCM, r.t., 45 min, 90 %; f) HATU, DIPEA, DCM (1 mm), r.t., 16 h, 51 %. DIPEA=*N,N‐*Diisopropylethylamine.

The macrocycle **9** with its terminal alkene offered the possibility of functionalization via cross‐metathesis. The thiol was introduced as a thioester like in largazole.^[8]^ We followed a two‐step‐sequence which had successfully been applied in the recent synthesis of homolargazole by our group.^[26]^ The introduction of homoallylic bromide (**29**) with Grubbs II catalyst at 80 °C followed by nucleophilic substitution of the bromide with thioacetate afforded the desired compound **32** (Scheme 7). The silylether was cleaved under acidic conditions to yield the pseudo‐natural product **33**. For another pseudo‐natural product with the same depsipeptide backbone, but with a hydroxamic acid warhead, **9** was subjected to cross‐metathesis with **34** (Scheme 7).^[27]^ Removal of TBS with HCl in acetone followed by cleavage of the Boc‐groups with TFA and triethylsilane delivered the desired hydroxamic acid **8**. Formation of the *Z* isomers was not observed during cross‐metathesis of **9**.

**Scheme 7:**
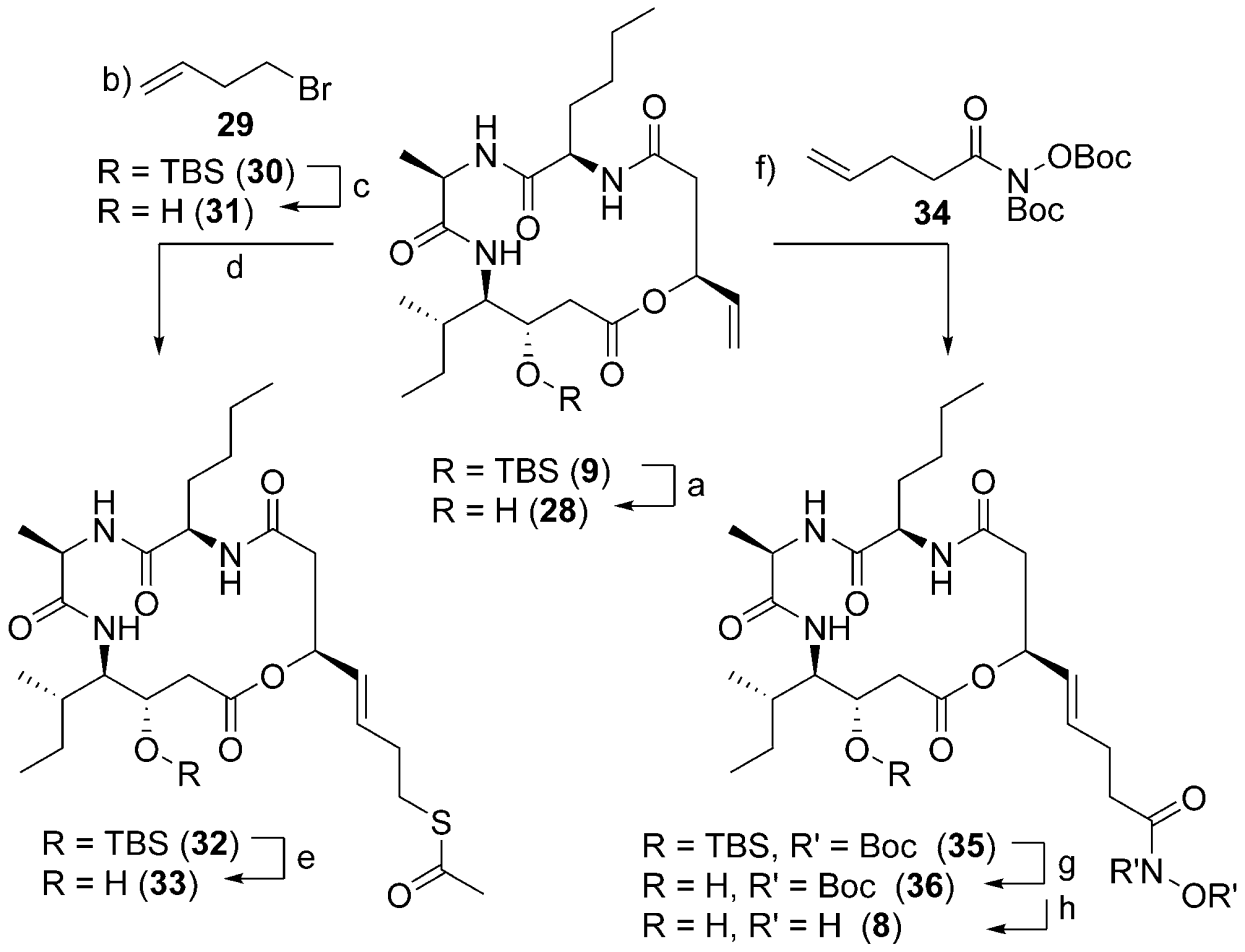
Late stage modification of **9**. a) HCl, acetone, r.t., 2.5 h, 66 %; b) Grubbs II catalyst (20 mol %), toluene, 80 °C, 18 h, 69 % brsm; c) HCl, acetone, r.t., 3 h, 78 %; d) potassium thioacetate, acetone, r.t., 3 h, 78 %; e) HCl, acetone, r.t., 3 h, 61 %; f) Grubbs II catalyst (20 mol %), toluene, 80 °C, 18 h, 52 % brsm; g) HCl, acetone, r.t., 2.5 h, 58 %; h) TFA, Et_3_SiH, DCM, r.t., 5 min.

The activity of compounds **9**, **28**, **32**, **33**, **35** and **8** was tested in assay systems for HDAC1, 6 and 8 (Table 2).^[28]^ The ring structures **9** and **28** without a warhead did not show inhibition for any of the classes of HDACs. Compound **32** containing the thioester and the alcohol protected by TBS showed a moderate inhibitory activity of HDAC1 (class I) with an IC_50_ of 18.7 μm, whereas HDAC6 (class II) was not inhibited. However, the deprotected compound **33** showed a significantly higher activity with an IC_50_ of 12.3 nm. Noteworthy is the outstanding HDAC1 selectivity. For the hydroxamic acid, the fully protected compound **35** was not active in HDAC inhibition. With its free hydroxy and hydroxamic acid, **8** had an IC_50_ of 28.0 nm for HDAC1. Hence, it was in the same range like thioester **33** and showed the same HDAC1 selectivity even with the hydroxamic acid warhead as a very strong zinc‐binding group. In comparison to IC_50_ values of the reduced (free thiol) Thailandepsin B (2*, Table 2),^[1a]^ our newly developed compounds **8** and **33** show superior HDAC1 selectivity while preserving nanomolar potency.

**Table 2:** HDAC inhibition of prepared compounds using a ZMAL trypsin assay for HDAC1 and 6 and a ZMTFAL trypsin assay for HDAC8;^[26]^ IC_50_ or inhibition @10 μm; n.i.=<5 % inhibition @10 μm.; n. d.=not determined. [a] literature data; **2** was reduced to the active thiol prior to being assayed, for assay details see Supporting Information.^[1a]^

Comp.	HDAC1	HDAC6	HDAC8
9	n. i.	n. i.	n. d.
28	n. i.	n. i.	n. d.
32	18.7±8.9 μm	n. i.	30 %
33	12.3±1.6 nm	30 %	22 %
35	n. i.	n. i.	n. d.
8	28.0±2.0 nm	39 %	21 %
2^[a]^	6.5 nm ^[1a]^	610 nm ^[1a]^	1000 nm ^[1a]^

Both pseudo‐natural products **33** and **8** are highly selective HDAC1 inhibitors. As expected, the newly introduced amino acid d‐alanine instead of d‐cysteine compared to thailandepsin B did not influence the inhibitory activity of the compounds. Most likely, the macrocyclic depsipeptide as backbone drives the subclass selectivity which was maintained with the strong zinc‐binding hydroxamic acid. Protection of the hydroxy group with bulky TBS decreased the activity. The active compounds **32**, **33** and **8** were subjected to cellular testing. In cell viability tests with HL60 (acute promyelotic leukemia) and HeLa cells (cervix carcinoma), micromolar GI_50_ values were obtained for **32** and nanomolar ones for **33. 8** did not inhibit growth of HL60 cells in the MTS assay at a concentration of 5 μm and 30 % inhibition of cell growth was detected for **8** at the same concentration in HeLa cells. HDAC inhibition in cells was determined with a cellular HDAC activity assay. Micromolar IC_50_ values were obtained for **32** for HL60 cells (66.3±3.5 μm) and HeLa cells (58.6±1.3 μm). For **33**, the IC_50_ values were nanomolar (HL60: 9.1±0.8 nm; HeLa: 34.0±2.2 nm). Hence, they were in the same range as in vitro. For **8**, no HDAC inhibition was found in cells at a concentration of 5 μm. Additionally, isotype selectivity for class I HDACs in cells was investigated by western blotting. Constant levels of acetylated α‐tubulin and increasing levels of acetylated histone 3 with increasing inhibitor concentration confirmed selective inhibition of class I HDACs for compound **33** in cells. Activity of **8** in vitro, but not in cells led to the conclusion that **8** might not be cell‐permeable in contrast to the thioester prodrugs.

In summary, we have developed a highly selective route for the syntheses of new thailandepsin B pseudo‐natural products. Rhodium‐catalyzed addition of the statine **24** to an allene was applied to build up the chiral allylic ester stereoselectively with a high d.r. of 88:12. Thioester and hydroxamic acid warheads were introduced via cross metathesis as a late stage modification of the macrocyclic backbone. The HDAC inhibitory activity of the new pseudo‐natural products was tested and insights to the structure–activity‐relationship of thailandepsins were obtained. Thioester **33** and hydroxamic acid **8** are highly potent HDAC1 inhibitors with nanomolar IC_50_ values in vitro. The synthetic strategy provides a platform for the introduction of varying warheads to the cyclic depsipeptide backbone on late stage. Different linker lengths and other zinc‐binding functional groups are the goal of future investigations.

## Experimental Section

[Rh(COD)Cl]_2_ (4.9 mg, 0.010 mmol, 4.5 mol %), *(R,R)*‐DIOP (9.9 mg, 0.020 mmol, 9.0 mol %) and statine **24** (113 mg, 0.220 mmol, 1.0 equiv.) were placed in a flame‐dried argon‐purged Young Schlenk round‐bottom flask. The flask was connected to high vacuum (4 h). Then, freshly distilled DCE (2.2 mL, 0.1 m) was added. The solution was stirred for 5 min before allene **25** (52 mg, 0.26 mmol, 1.2 equiv.) filtered over basic allox was added at r.t. With addition of the allene, the color of the reaction mixture turned from orange to light yellow. The reaction was allowed to stir at 10 °C for 48 h. A crude NMR was taken to determine the d.r. from the allylic signal. The crude was purified by column chromatography (silica gel, PE:EA 95:5–90:10) to provide the product **26** as colorless oil (mixture of diastereomers, 119 mg, 0.168 mmol, 80 %). The d.r. was determined to be 88:12 from the crude ^1^H NMR.

## Conflict of interest

The authors declare no conflict of interest.

## Supporting information

As a service to our authors and readers, this journal provides supporting information supplied by the authors. Such materials are peer reviewed and may be re‐organized for online delivery, but are not copy‐edited or typeset. Technical support issues arising from supporting information (other than missing files) should be addressed to the authors.

Supplementary
