# Supplementary material for: Syntheses of Thailandepsin B Pseudo‐Natural Products: Access to New Highly Potent HDAC Inhibitors via Late‐Stage Modification
Source: Chemistry. 2020 Nov 3;26(69):16241–5. doi: 10.1002/chem.202002449 (PMC7756392; doi:10.1002/chem.202002449)
Supplement: Supplementary file 1 — Supplementary [file CHEM-26-16241-s001.pdf]

Chemistry–A European Journal

Supporting Information

Syntheses of Thailandepsin B Pseudo-Natural Products: Access to New Highly Potent HDAC Inhibitors via Late-Stage Modification

Jana Brosowsky,[a] Monika Lutterbeck,[a] Amelie Liebich,[a] Manfred Keller,[a] Daniel Herp,[b] Anja Vogelmann,[b] Manfred Jung,[b] and Bernhard Breit*[a]

Table of Contents

General Experimental and Analytical Techniques 2

Synthesis of D-Norleucine via Rhodium catalyzed Hydroamination.....................................6

Synthesis of the Dipeptide 11...13

Rhodium-catalyzed hydrooxycarbonylation of statine 24 to allene 25...............................15

Determination of the absolute configuration of the newly formed stereocenter 20

Coupling of Northern and Southern Part and further Conversion.....................................23

Functionalization of the Precursor 9 towards a Thailandepsin B Alanine Derivative 32

Functionalization of the Precursor 9 towards a Thailandepsin B Alanine Derivative with Hydroxamic acid Warhead.. 37

Assay Methods 42

Spectral Data...45

References 73

General Experimental and Analytical Techniques

All herein performed reactions and analytical investigations were conducted in the research group of Prof. Dr. B. BREIT in the Department for Organic Chemistry at Albert-Ludwigs-University of Freiburg.

All reactions were performed in glassware that had been evacuated, flame-dried by a heat gun and backfilled with argon (Argon 5.0, SAUERSTOFFWERKE FRIEDRICHSHAFEN) with magnetic stirring under argon atmosphere unless otherwise noted. All commercial reagents were used without purification unless otherwise noted. Air and moisture-sensitive liquids and solutions were transferred via stainless steel needle and introduced into the reaction vessel through rubber septa. Needles have been dried at 50 °C and purged with argon before use. Reactions conducted below room temperature were cooled by an external bath: dry ice in acetone for -78 °C or ice in water for 0 °C. If a temperature below room temperature had to be hold over night or longer, a cryostate filled with ethylenglycol/water was used to cool down the external bath.

Rhodium-catalysis reactions were run in a screw-cap flask with a YOUNG cap.

For needs of high vacuum, a rotary vane vacuum pump from VACUUBRAND GMBH & CO. KG (pressure < 0.1 mbar) was used.

Chromatography

Thin-layer Chromatography (TLC)

Thin-layer chromatography was performed on pre-coated silica gel TLC-plates SIL G-25 UV_{254} (0.25 mm) from MACHEREY-NAGEL GMBH & CO. KG. As eluents, PE/EA and DCM/MeOH were used in various ratios. Visualization of the developed chromatogram was performed at UV-light at a wavelength of 254 nm and staining with one of the following solutions followed by drying with a heat gun:

$KMNO_4$ stain:	$KMnO_4$ (3.0 g), Na_2CO_3 (20 g), aq. NaOH (5%, 5 ml), water (300 ml).
Cer-MOPS:	Phosphomolybdic acid (6.5 g), Cerium (IV)-sulfate (2.5 g), H_2SO_4 (conc., 12 ml), water (230 ml).
Vanillin stain:	Vanillin (10 g) H_2SO_4 (conc, 1 mL) in EtOH (250 mL).

Column Chromatography

Chromatographic purification of products was accomplished using flash column chromatography on silica gel 60 (particle size 0.040-0.063 mm) from MACHERY- NAGEL GMBH & CO. KG. Columns were packed with a plug of cotton, a layer of seasand, a silica gel layer of various height and another layer of seasand.

Evaporation of solvents

Solvents were removed under reduced pressure at 40 °C on a rotation evaporator Laborota 4001 efficient from HEIDOLPH. Reaction mixtures were further concentrated on high vacuum.

Melting points

Melting points were determined with a SMP10 capillary melting point apparatus from STUART according to DR. TOTTOLI and are uncorrected.

Optical Rotation

Angles of rotation were measured with a P8000-T Polarimeter from A. KRÜSS OPTRONIC GMBH. Specific rotation $[\alpha]_D^T$ was calculated with the formula

$$[\alpha]_D^T = \frac{\alpha \cdot 100}{c \cdot d}$$

T = temperature in °C, D = sodium D-line emission, α = angle of rotation, c = concentration in g/100 ml, d = length of polarimeter tube in dm (here 1 dm).

Nuclear Magnetic Resonance Spectroscopy (NMR)

^{1}H- and ^{13}C- spectroscopy was performed on a BRUKER Avance 500 operating at 500 MHz (^{1}H) and 125 MHz (^{13}C), a BRUKER Avance 400 operating at 400 MHz (^{1}H) and 100 MHz (^{13}C) or a BRUKER Avance 300 NMR operating at 300 MHz (^{1}H) and 75 MHz (^{13}C). Chemical shifts are reported in ppm relative to residual proton solvent signals: $CDCl_3$ (δ = 7.26 ppm), C_6D_6 (δ = 7.16 ppm), $(CD_3)_2SO$ (δ = 2.50 ppm), CD_3OD (δ = 3.31, 4.87 ppm), $(CD_3)_2CO$ (δ = 2.05 ppm) and D_2O (δ = 4.75 ppm), respectively. All ^{13}C-NMR spectra are proton decoupled, chemical shifts are referenced to the signal of the deuterated solvent: $CDCl_3$ (δ = 77.0 ppm), C_6D_6 (δ = 128.4 ppm), $(CD_3)_2SO$ (δ = 39.5 ppm) and CD_3OD (δ = 49.2 ppm). Data for ^{1}H are reported in terms of chemical shift (δ ppm), multiplicity (s = singlet, d = doublet, t = triplet, q = quartet, m = multiplet, br. s. = broad singlet), coupling constant, and integration; data for ^{13}C are reported in terms of chemical shift.

Mass Spectrometry (MS)

High resolution mass spectrometry (ESI-HRMS or APCI-HRMS with positive or negative ion mode) was performed on an Exactive FT-Mass Spectrometer from THERMO FISHER SCIENTIFIC INC. with orbitrap analyzer with a mass resolution up to 100.000 and a mass accurancy better than 5 ppm.

Electronspray ionization mass spectrometry (ESI) was performed on a LCQ Advantage or Exactive mass spectrometer from Thermo Fisher Scientific Inc. At the LCQ Advantage instrument, 2.5 μL/min of the sample solution were injected into a flow of 100 - 200 μL/min of methanol or acetonitrile. The spray voltage was 4 – 5 kV. The ion transfer tube had a temperature of 250 - 300 °C.
Atmospheric pressure chemical ionization mass spectrometry (APCI) was performed on a LCQ Advanatge instrument, 2.5 μl/min of the sample solution were injected into a flow of 200 - 400 μL/min of methanol or acetonitrile. The spray current was 5 μA. The ion transfer tube had a temperature of 150 - 180 °C; the vaporizer had a temperature of 300 - 400 °C.
For gas chromatography-mass spectrometry (GC-MS) the TSQ 700 mass spectrometer (EI or CI) was connected to a 3400 gas chromatograph from Varian Inc.
For liquid chromatography-mass spectrometry (LC-MS) the LSQ Advantage mass spectrometer was connected to a Surveyor LC system from Thermo Fisher Scientific Inc.
resolution of M/ΔM = 20 0000 - 100 000.
All measurements of mass spectrometry have been done in the analytics department of the Institute of Organic Chemistry at Albert-Ludwigs-University of Freiburg

High Pressure Liquid Chromatography

Chiral HPLC measurements were performed on a Merck Hitachi HPLC apparatus (pump: L-7100, UV detector: L-7400, auto sampler: L-7200, oven: L-7360), a Varian Pro Star (pump: 230, UV detector: 310, auto sampler: 410,moven: 510) and a Hitachi Primade (pump: 1100, DAD detector: 1430, auto sampler: 1210, oven: 1310).

Solvents

Solvents for extraction and chromatography were purchased in technical grade and purified by rotary evaporation if necessary.
If required, solvents used for reactions were dried and purified and kept under argon.
Tetrahydrofuran was heated to reflux over potassium and freshly distilled under argon.
Toluene was heated to reflux over potassium and freshly distilled under argon.
1,2-Dichloroethane was heated to reflux over calcium hydride and freshly distilled under argon.
Other solvents were dried in a Solvent Purification System 800 from BRAUN where they were pressed through two columns under argon before filled in evacuated, flame-dried and argon-purged flasks.
Diethylether: column 1: aluminum oxide, 2. column: molecular sieves (2Å).
Dichloromethane: column 1 and 2: aluminum oxide.

Nomenclature

Structural formula was created in ChemDraw Professional 17.0. Nomenclature was automatically suggested by ChemDraw Professional 17.0. In some cases, trivial names were preferred.

Synthesis of D-Norleucine via Rhodium catalyzed Hydroamination

Hepta-1,2-diene (15)

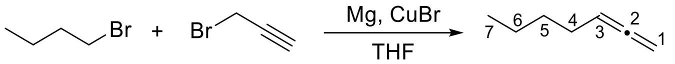

C_3H_7Br	C_3H_3Br		C_7H_{12}
122.99	118.96		96.17
**38**	**39**		**15**

To flame-dried Mg (2.48 g, 102 mmol, 1.2 eq.) in THF (30 ml) was added 2 ml of 1-bromobutane (**38**, 10.7 ml, 13.7 g, 100 mmol, 1.2 eq.) until the Grignard reaction started (refluxing observed). The remaining amount of 1-bromobutane was diluted with THF (50 ml) and added slowly *via* dropping funnel. Heating was started to keep the reaction mixture refluxing. After full addition, the reaction mixture was heated to reflux for 2 h. Then, it was allowed to come to r.t. and was cooled to -78 °C. LiBr (2.35 g, 27.0 mmol, 0.31 eq.) and CuBr (1.15 g, 8.00 mmol, 0.092 eq.) were added with THF (20 ml) at this temperature, followed by dropwise addition of propargyl bromide (**39**, 6.50 ml, 10.3 g, 86.3 mmol, 1.0 eq.) in THF (50 ml) during 30 min. The color turns to green/yellow. The reaction mixture was allowed to stir for 30 min at -78 °C and additional 30 min at r.t. before being quenched with sat. NH_4Cl (200 ml).The aq. phase was extracted with pentane (3×150 ml). The combined org. layers were washed with water and brine and dried over Na_2SO_4. Purification by distillation failed. Column chromatography (silica gel, pentane) delivered the desired product **15** as colorless liquid (2.26 g, 23.5 mmol, 27%) with traces of pentane after rotary evaporation at 0 °C. The product is extremely volatile.

^{1}H-NMR (400 MHz, $CDCl_3$): δ = 0.91 (t, *J* = 7.1 Hz, 3H, C^7-H_3), 1.30 - 1.45 (m, 4H, C^5-H_2 and C^6-H_2), 1.97 - 2.04 (m, 2H, C^4-H_2), 4.65 (dt, *J* = 6.6, 3.3 Hz, 2H, C^1-H_2), 5.09 (quin, *J* = 6.8 Hz, 1H, C^3-H) ppm.
^{13}C-NMR (101 MHz, $CDCl_3$): δ = 13.9 (C^7-H_3), 22.2 (C^6-H_2), 28.1 (C^5-H_2), 31.4 (C^4-H_2), 74.6 (C^1-H_2), 90.2 (C^3-H), 208.6 (C^2) ppm.
HRMS (pos. APCI): Calcd for $C_7H_{16}N$ $[M+NH_4]^+$:. 114.1277. Found 114.1278.
Spectral data matched with those reported for this compound.[1]

One-pot synthesis of allylic amides

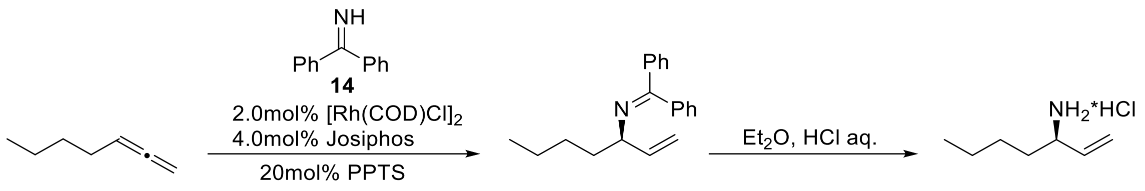

C_7H_{12}
96.17
15

$C_{20}H_{23}N$
277.41
16

$C_7H_{16}ClN$
149.66
40

General procedure 1:[2]

Hydroamination: To an Argon-purged and flame-dried Young tube was added $[Rh(COD)Cl]_2$ (3.94 mmol, 0.008 mmol, 2 mol%), commercially available Josiphos-J003-1 (0.016 mmol, 4 mol%), PPTS (20.1 mg, 0.080 mmol, 20 mol%), benzophenone imine (72.5 mg, 0.400 mmol, 1.0 eq.), DCE (1 ml, 0.4 M) and hepta-1,2-diene (57.7 mg, 0.600 mmol, 1.5 eq.). The Young tube was sealed and the reaction mixture was allowed to stir for 18 h at 80 °C. After cooling to r.t., the solvent was removed under reduced pressure. The crude NMR was taken with DMF as internal standard to determine the NMR-yield.

Hydrolysis: Et_2O (2.00 ml) and aq. HCl (2 M, 2.00 ml, 4.00 mmol, 10 eq.) were added. The reaction mixture was allowed to stir for 24 h. Volatiles were removed aceotropically with acetone under reduced pressure.

(9*H*-Fluoren-9-yl)methyl (*R*)-hept-1-en-3-ylcarbamate (17)

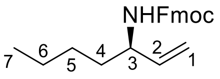

$C_{22}H_{25}NO_2$
335.45
17

The reaction was performed following the general procedure **1**. For amide formation, an aq. solution of Na_2CO_3 (10%, 1.1 ml) and dioxane (1.4 ml) were added. The solution was cooled to 0 °C before Fmoc-Cl (114 mg, 0.440 mmol, 1.1 eq.) in dioxane (0.5 ml, additional 0.4 ml to rinse the flask) was added. The reaction mixture was allowed to stir for 17 h. Water and DCM were added. Layers were separated and the aq. phase was extracted with DCM (3×). The combined org. layers were dried over Na_2SO_4 and the solvent was removed under reduced pressure. Purification by column chromatography (silica gel, PE/EA 9.5:0.5 – 9:1) delivered the desired product **17** as colorless solid (97.9 mg, 0.292 mmol, 73%).

Mp.: 127 - 129 °C.

$[\alpha]_D^{20}$ = -8.2, c = 1.0, $CHCl_3$.

^{1}H-NMR (500 MHz, $CDCl_3$): δ = 0.90 (t, *J* = 6.9 Hz, 3H, C^7-*H_3*), 1.27 - 1.38 (m, 4H, C^5-*H_2* and *C^6*-*H_2*), 1.42 - 1.51 (m, 1H, C^4-*H_2*), 1.52 – 1.58 (m, 1H, C^4-*H_2*), 4.11 -4.19 (m, 1H, C^3-*H*), 4.23 (t, *J* = 6.8 Hz, 1H, *Fmoc*-C*H*), 4.43 (d, *J* = 6.7 Hz, 2H, *Fmoc*-C*H_2*), 4.64 (br. s., 1H, C^3-N*H*), 5.11 (dd, *J* = 16.9, 10.2 Hz, 2H, C^1-*H_2*), 5.70 - 5.81 (m, 1H, C^2-*H*), 7.32 (td, *J* = 7.5, 1.1 Hz, 2H, Ar-C*H*), 7.40 (tt, *J* = 7.5, 0.9 Hz, 2H, Ar-C*H*), 7.60 (dd, *J* = 7.5, 0.9 Hz, 2H, Ar-C*H*), 7.77 (d, *J* = 7.5 Hz, 2H, Ar-C*H*) ppm.

^{13}C-NMR (126 MHz, $CDCl_3$): δ = 14.1 (*C^7*-H_3), 22.5 (*C^6*-H_2), 27.9 (*C^5*-H_2), 34.9 (*C^4*-H_2), 47.5 (*Fmoc*-*C*H), 53.4 (*C^3*-H), 66.5 (*Fmoc*-*C*H_2), 114.7 (*C^1*-H_2), 120.0 (Ar-*C*H), 125.1 (Ar-*C*H), 127.1 (Ar-*C*H), 127.7 (Ar-*C*H), 138.9 (*C^2*-H), 141.4 (Ar-C_{quart}), 144.1 (Ar-C_{quart}), 155.9 (*C*(=O)) ppm.

HRMS (pos. ESI): Calcd for $C_{22}H_{25}O_2NNa$ $[M+Na]^+$: 358.1778. Found 358.1776.

HPLC: 96% *ee*; t_R = 15.83 min (major) and 17.14 min (minor), [LC-2, heptane/IPA 98:2, 0.7 ml/min, 267 nm, 22 °C].

Benzyl (*R*)-hept-1-en-3-ylcarbamate (18)

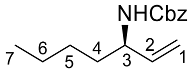

$C_{15}H_{21}NO_2$
247.33
18

The reaction was performed following the general procedure **1.** For amide formation, a solution of K_2CO_3 (138 mg, 1.00 mmol, 2.5 eq.) in water (0.6 ml) and ethyl acetate (0.6 ml) were added. The solution was cooled to 0 °C before Cbz-Cl (72 mg, 0.44 mmol, 1.1 eq.) was added dropwise. The reaction mixture was allowed to stir for 17 h. Water and EA were added. Layers were separated and the aq. phase was extracted with EA (3×). The combined org. layers were washed with HCl (1 M, 2×) and brine (1×) and dried over Na_2SO_4. The solvent was removed under reduced pressure. Purification by column chromatography (silica gel, PE/EA 20:1 – 15:1 – 9:1) delivered the desired product **18** as colorless oil (57 mg, 0.23 mmol, 58%).

$[\alpha]_D^{20}$ = -12.0, c = 1.0, $CHCl_3$.

^{1}H-NMR (400 MHz, $CDCl_3$): δ = 0.86 - 0.93 (m, 3H, C^7-*H_3*), 1.28 - 1.38 (m, 4H, C^5-*H_2* and *C^6*-*H_2*), 1.43 - 1.60 (m, 2H, C^4-*H_2*), 4.10 - 4.22 (m, 1H, C^3-*H*), 4.70 (br. s., 1H, C^3-N*H*), 5.09 (ddd,

J = 12.6, 1.4 Hz (×2), 3H, *cis*-C[1]-*H*$_2$ and Cbz-C*H*$_2$), 5.16 (d, *J* = 17.2 Hz, 1H, *trans*- C[1]-*H*$_2$), 5.76 (ddd, *J* = 17.1, 10.5, 5.7 Hz, 1H, C[2]-*H*), 7.28 - 7.35 (m, 1H, Ar-C*H*), 7.34 - 7.38 (m, 4H, Ar-C*H*) ppm.

^{13}C-NMR (101 MHz, $CDCl_3$): δ = 14.0 (*C*[7]-H_3), 22.5 (*C*[6]-H_2), 27.8 (*C*[5]-H_2), 34.9 (*C*[4]-H_2), 53.4 (*C*[3]-H_2), 66.7 (Cbz-*C*H_2), 114.6 (*C*[1]-H_2), 128.1 (Ar-*C*H), 128.6 (Ar-*C*H), 136.7 (Ar-C_{quart}), 138.9 (*C*[2]-H), 155.9 (*C*(=O)) ppm.

HRMS (pos. ESI): Calcd for $C_{15}H_{21}O_2NNa$ $[M+Na]^+$: 270.1465. Found 270.1466.

(9*H*-Fluoren-9-yl)methyl-((3*R*)-1,2-dihydroxyheptan-3-yl)carbamate (41)

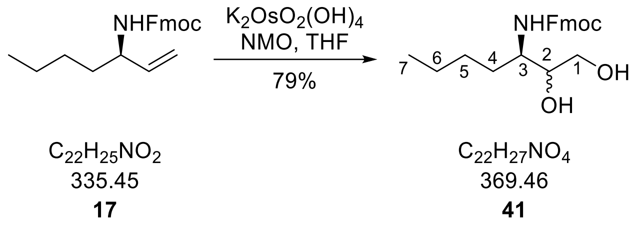

To a solution of **17** (85 mg, 0.25 mmol, 1.0 eq.) in acetone (0.65 ml), water (0.65 ml) and THF (0.95 ml) were added NMO (59 mg, 0.51 mmol, 2.0 eq.) and $K_2OsO_2(OH)_4$ (3.7 mg, 0.010 mmol, 4 mol%). The reaction mixture was allowed to stir over night before aq. $NaHSO_3$ was added. Phases were separated and the aq. phase was extracted with DCM (3×). The combined org. layers were washed with brine and dried over Na_2SO_4. Purification by column chromatography (silica gel, DCM/MeOH 50:1 – 25:1) delivered the desired product as colorless solid (79 mg, 0.21 mmol, 79%). The product **41** was obtained as a mixture of diastereomers which was used in the next step without separation.

Mp.: 119 °C.

^{1}H-NMR (400 MHz, $CDCl_3$): δ = 0.91 (t, *J* = 5.8 Hz, 3H), 1.24 - 1.41 (m, 4H), 1.56 (br. s., 1H), 1.83 (br. s., 1H), 2.48 (br. s., 2 H), 3.32 - 3.38 (m, 1H), 3.45 - 3.61 (m, 3H), 3.67 - 3.76 (m, 1H), 4.21 (t, *J* = 6.4 Hz, 1H), 4.43 - 4.60 (m, 1H), 4.73 (d, *J* = 8.3 Hz, 1H), 4.88 - 4.96 (m, 1H), 7.30 - 7.36 (m, 2H), , 7.38 - 7.44 (m, 2H), 7.56 - 7.62 (m, 2H), 7.75 - 7.80 (m, 2H) ppm.

^{13}C-NMR (101 MHz, $CDCl_3$): δ = 13.9, 22.4, 28.2, 29.7, 30.7, 31.8, 47.4, 51.9, 53.2, 62.9, 63.8, 66.6, 73.2, 74.3, 77.2, 120.0, 124.8, 124.9, 127.1, 127.7, 141.4, 143.6, 143.7, 143.8, 143.8, 157.4, 157.6 ppm.

Due to a mixture of diastereomers, peaks were not assigned.

(*R*)-((9*H*-Fluoren-9-yl)methoxy)carbonyl-D-norleucine (19)

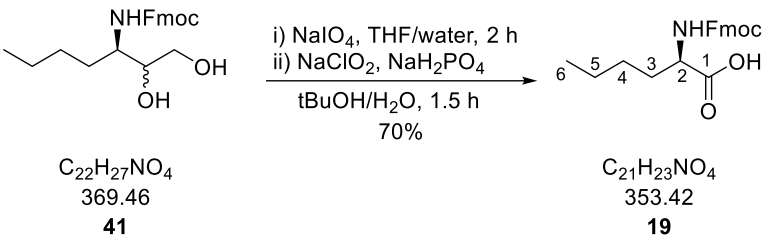

To the diol **41** (67 mg, 0.181 mmol, 1.0 eq.) in THF (1.2 ml) and water (1.0 ml) was added $NaIO_4$ (116 mg, 0.543 mmol, 3.0 eq.). The reaction mixture was allowed to stir for 2 h at r.t. before solvents were removed under reduced pressure.

THF (0.6 ml) and tBuOH (1.8 ml) were added, followed by $NaClO_2$ (164 mg, 1.81 mmol, 10 eq.) and KH_2PO_4 (246 mg, 1.81 mmol, 10 eq.) in water (2.1 ml). The reaction was completed after 1.5 h at r.t. Solvents were removed under reduced pressure. The remaining aq. phase was extracted with EA (3×). The combined org. layers were washed with brine (1×) and dried over Na_2SO_4. The solvent was removed in vacuo. Column chromatography (silica gel, DCM/EA/EtOH 6:1:0.1) delivered the desired product **19** as colorless solid (45 mg, 0.13 mmol, 70%).

Mp.: 145-147 °C.

$[\alpha]_D^{20}$ = -1.4, c = 1.0, $CHCl_3$.

^{1}H-NMR (400 MHz, DMSO-*d*$_6$): δ = 0.87 (t, *J* = 7.1 Hz, 3H, C^6-H_3), 1.21 - 1.36 (m, 4H, C^5-H_2, C^4-H_2), 1.56 - 1.65 (m, 1H, C^3-H_2), 1.66 - 1.76 (m, 1H, C^3-H_2), 3.89 - 3.97 (m, 2H, C^2-*H*), 4.19 - 4.25 (m, 1H, *Fmoc*-C*H*), 4.26 - 4.31 (m, 2H, *Fmoc*-CH_2), 7.33 (tt, *J* = 7.5, 1.3 Hz, 2H, Ar-C*H*), 7.42 (td, *J* = 7.5, 0.7 Hz, 2H, Ar-C*H*), 7.60 (d, *J* = 8.1 Hz, 1H, C^2-N*H*), 7.73 (dd, *J* = 7.5, 0.9 Hz, 2H, Ar-C*H*), 7.89 (d, *J* = 7.6 Hz, 2H, Ar-C*H*), 12.50 (br. s., 1H, C(=O)O*H*) ppm.

^{13}C-NMR (101 MHz, DMSO-*d*$_6$): δ = 13.7 (C^6-H_3), 21.6 (C^5-H_2), 27.6 (C^4-H_2), 30.4 (C^3-H_2), 46.6 (*Fmoc*-*C*H), 53.7 (C^2-H s), 65.5 (*Fmoc*-*C*H_2), 120.0 (Ar-*C*H), 125.2 (Ar-*C*H), 127.0 (d, Ar-*C*H), 127.6 (Ar-*C*H), 140.7 (d, Ar-C_{quart}), 143.8 (d, Ar-C_{quart}), 156.1 (*C*(=O)), 173.9 (*C*(=O)OH) ppm.

HRMS (pos. ESI): Calcd for $C_{21}H_{23}NO_4Na$ $[M+Na]^+$: 376.1519. Found 376.1525.

HPLC: >99% *ee*; t_R = 6.90 min (minor) and 7.48 (major), [AD-3, heptane/EtOH 75:25, 0.8 ml/min, 266 nm, 22 °C].

Analytical data matched with those reported for this compound.[3]

Benzyl-((3*R*)-1,2-dihydroxyheptan-3-yl)carbamate (42)

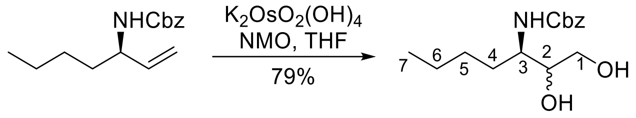

To a solution of **19** (55 mg, 0.22 mmol, 1.0 eq.) in acetone (0.55 ml) and water (0.55 ml) were added NMO (52 mg, 0.44 mmol, 2.0 eq.) and $K_2OsO_2(OH)_4$ (3.3 mg, 90 µmol, 4mol%). The reaction mixture was allowed to stir over night before aq. $NaHSO_3$ was added. Phases were separated and the aq. phase was extracted with $CHCl_3$ (3×). The combined org. layers were washed with brine and dried over Na_2SO_4. Purification by column chromatography (silica gel, DCM/MeOH 25:1) delivered the desired product as colorless solid (57 mg, 0.21 mmol, 91%). The product **42** was obtained as a mixture of diastereomers which was used in the next step without separation.

Mp.: 109 °C.

^{1}H-NMR (400 MHz, $CDCl_3$): δ = 0.90 (t, *J* = 6.3 Hz, 3H), 1.26 - 1.44 (m, 5H), 1.51 - 1.59 (m, 1H), 1.74 - 1.83 (m, 1H), 3.06 (br. s., 2H), 3.43 (br. s., 0.5H), 3.54 (d, *J* = 6.3 Hz, 1H), 3.59 - 3.64 (m, 2H), 3.67 - 3.73 (m, 1H), 4.91 - 5.00 (br. s, 1H), 5.09 - 5.12 (m, 2H), 7.30 - 7.39 (m, 5H) ppm.

^{13}C-NMR (101 MHz, $CDCl_3$): δ = 13.9, 22.4, 22.5, 28.1, 28.2, 30.7, 31.8, 52.0, 53.3, 63.1, 63.9, 67.0, 67.1, 73.2, 74.2, 128.0, 128.0, 128.1, 128.2, 128.5, 128.5, 136.2, 136.3, 157.3, 157.5 ppm.

HRMS (pos. ESI): Calcd for $C_{15}H_{23}O_4NNa$ $[M+Na]^+$: 304.1519. Found 304.1521.

Due to a mixture of diastereomers, peaks were not assigned.

(*R*)-(((Benzyloxy)carbonyl)amino)-D-norleucine (20)

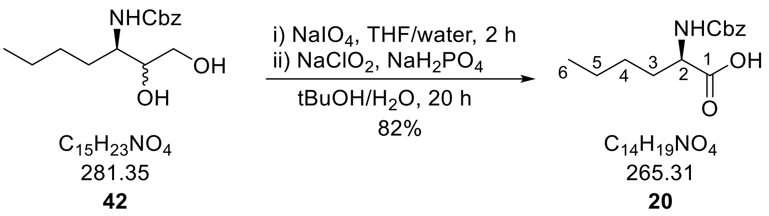

To the diol **42** (56.8 mg, 0.202 mmol, 1.0 eq.) in THF/water (1:1, 2.2 ml) was added $NaIO_4$ (116 mg, 0.586 mmol, 2.7 eq.). The reaction mixture was allowed to stir for 2 h at r.t. before solvents were removed under reduced pressure.

The residue was dissolved in THF/*t*BuOH (1:3, 2.5 ml). $NaClO_2$ (182 mg, 2.02 mmol, 10 eq.) and NaH_2PO_4 (242 mg, 2.02 mmol, 10 eq.) in water (2.5 ml) were added. The reaction mixture was allowed to stir for 20 h at r.t. The org. solvents were removed under reduced pressure and the aq. phase was extracted with EA (3×). The combined org. layers were washed with brine (1×) and dried over $MgSO_4$. The solvent was removed in vacuo. Column chromatography (silica gel, DCM/MeOH 40:1) delivered the desired product **20** as colorless oil (44 mg, 0.166 mmol, 82%).

$[\alpha]_D^{20}$ = -2.0, c = 1.0, $CHCl_3$.

^{1}H-NMR (400 MHz, $CDCl_3$): δ = 0.90 (t, *J* = 6.7 Hz, 3H, C^6-H_3), 1.26-1.40 (m, 4H, C^5-H_2, C^4-H_2), 1.65-1.94 (m, 2H, C^3-H_2), 4.40 (td, *J* = 7.8 Hz, *J* = 5.0 Hz, 1H, C^2-*H*), 5.26 (br. s, 1H, C^2-N*H*), 5.08-5.18 (m, 2H, Cbz-CH_2), 7.29-7.37 (m, 5H, Ar-C*H*), 9.76 (br. s, 1H, C^1(=O)O*H*) ppm.

^{13}C-NMR (101 MHz, $CDCl_3$): δ = 13.9 (C^6-H_3), 22.4 (C^5-H_2), 27.4 (C^4-H_2), 32.3 (C^3-H_2), 53.9 (C^2-H), 67.3 (Cbz-*C*H_2), 128.2 (Ar-*C*H), 128.4 (Ar-*C*H), 128.7 (Ar-*C*H), 136.3 (Ar-C_{quart}), 156.2 (*C*(=O)); 177.6 (*C*(=O)OH) ppm.

HRMS (pos. ESI): Calcd for $C_{14}H_{19}O_4NNa$ $[M+Na]^+$: 288.1206. Found 288.1207.

HPLC: >99% *ee*; t_R = 10.09 min (minor) and 16.27 (major), [AD-3, heptane/IPA 80:20, 0.5 ml/min, 212 nm, 22 °C].

Spectral data matched with those reported for this compound.[4]

Synthesis of the Dipeptide 12

(*R*)-2-((((9*H*-Fluoren-9-yl)methoxy)carbonyl)amino)hexanoic acid (19)

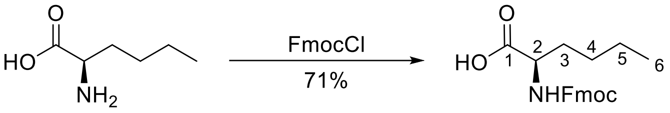

To a solution of D-norleucine (**43**, 1.50 g, 11.4 mmol, 1.0 eq.) in dioxane (26 ml) was added aq. Na_2CO_3 (10%, 51 ml). The reaction mixture was cooled to 0 °C before a solution of Fmoc-chloride (3.25 g, 12.6 mmol, 1.1 eq.) in dioxane (36 ml) was added dropwise at 0 °C. After being stirred for 2 h at r.t., the mixture was poured on water (450 ml). The aq. phase was washed with diethylether (2×150 ml) and then EA (180 ml) was added. The aq. phase was acidified with conc. HCl at 0 °C and extracted with EA (3×180 ml). The combined organic layers were washed with water (1×180 ml), dried over Na_2SO_4 and concentrated under reduced pressure. Recrystallization from PE/EE (1:1, 10 ml) delivered the desired product **19** as colorless solid (2.86 g, 8.09 mmol, 71%).

For analytical data see above.

tert-Butyl ((*R*)-2-((((9*H*-fluoren-9-yl)methoxy)carbonyl)amino)hexanoyl)-D-alaninate (22)

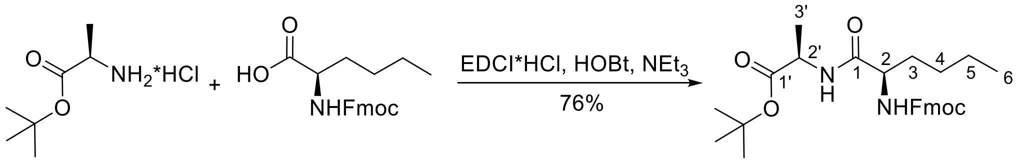

To HOBt (1.79 g, 13.3 mmol, 1.5 eq.) and EDCI*HCl (2.54 g, 13.3 g, 1.5 eq.) in DMF (149 ml) was added Fmoc-D-norleucine (**19**, 3.14 g, 8.89 mmol, 1.0 eq.). After 1 h at 0 °C, NEt_3 (1.4 ml, 1.0 g, 9.8 mmol, 1.1 eq.) was added, followed by D-alanine *tert*-butyl ester hydrochloride (**21**, 1.79 g, 9.78 mmol, 1.1 eq.) after 15 min. The reaction mixture was allowed to slowly warm up to r.t. and was stirred overnight. Water was added. The aq. phase was extracted with DCM (3×150 ml). The combined organic layers were washed with HCl (1 M, 150 ml), aq. $NaHCO_3$

(150 ml) and brine (3×150 ml) and dried over $NaSO_4$. Solvents were removed under reduced pressure. Purification by column chromatography (silica gel, PE/EE 3:1) and recrystallization from Et_2O (24 ml) delivered the desired product **22** as colorless solid (3.23 g, 6.72 mmol, 76%).

Mp.: 129 °C.

$[\alpha]_D^{20}$ = 5.4, c = 1.0, $CHCl_3$.

^{1}H-NMR (400 MHz, $CDCl_3$): δ = 0.91 (t, *J* = 6.8 Hz, 3H, C^6-H_3), 1.30 - 1.40 (m, 4H, C^5-H_2, C^4-H_2), 1.38 (d, *J* = 7.1 Hz, 3H, $C^{3'}$-H_2), 1.47 (s, 9H, $C(CH_3)_3$), 1.59 - 1.71 (m, 1H, C^3-H_2), 1.79 - 1.93 (m, 1H, C^3-H_2), 4.14 - 4.25 (m, 1H, C^2-*H*), 4.23 (t, *J* = 7.1 Hz, 1H, *Fmoc*-C*H*), 4.33 - 4.54 (m, 3H, $C^{2'}$-*H* and *Fmoc*-CH_2), 5.43 (d, *J* = 7.6 Hz, 1H, C^2-N*H*), 6.49 (d, *J* = 5.9 Hz, 1H, $C^{2'}$-N*H*), 7.31 (tt, *J* = 7.6, 1.3 Hz, 2H, Ar-C*H*), 7.37 - 7.43 (m, 2H, Ar-C*H*), 7.60 (d, *J* = 5.7 Hz, 2H, Ar-C*H*), 7.77 (dd, *J* = 7.6, 0.7 Hz, 2H, Ar-C*H*) ppm.

^{13}C-NMR (101 MHz, $CDCl_3$): δ = 13.8 (C^6-H_3), 18.5 ($C^{3'}$-H_3), 22.4 (C^5-H_2), 27.4 (C^4-H_2), 27.9 ($C(CH_3)_3$), 32.7 (C^3-H_2), 47.2 (*Fmoc*-*C*H), 48.7 ($C^{2'}$-H), 54.9 (C^2-H), 67.0 (*Fmoc*-*C*H_2), 82.1 ($C(CH_3)_3$), 119.9 (d, Ar-*C*H), 125.1 (Ar-*C*H), 127.0 (Ar-*C*H), 127.7 (Ar-*C*H), 141.3 (Ar-*C*H), 143.8 (d, Ar-C_{quart}), 156.1 (Ar-C_{quart}), 171.1 (*C*=O), 171.8 (*C*=O) ppm.

HRMS (pos. ESI): Calcd for $C_{28}H_{36}N_2O_5Na$ $[M+Na]^+$: 503.2516. Found 503.2524.

((*R*)-2-((((9*H*-Fluoren-9-yl)methoxy)carbonyl)amino)hexanoyl)-D-alanine (12)

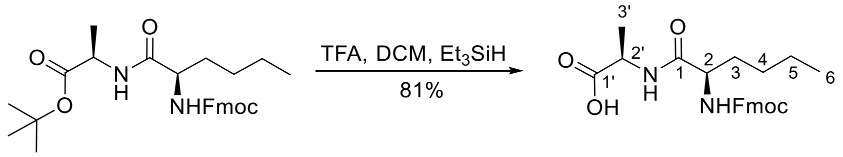

$C_{28}H_{36}N_2O_5$
480.61
22

$C_{24}H_{28}N_2O_5$
424.50
12

To a solution of **22** (1.86 g, 3.87 mmol, 1.0 eq.) in DCM (14 ml) were added triethylsilane (1.55 ml, 1.13 g, 9.68 mmol, 2.5 eq.) and trifluoroacetic acid (3.83 ml, 5.74 g, 50.3 mmol, 13 eq.). The reaction mixture was allowed to stir overnight at r.t. Solvents were removed under reduced pressure and co-evaporated with toluene (1×). The remaining colorless solid was digerated with DCM and filtered off. The desired product **12** was obtained as colorless solid (1.33 g, 3.13 mmol, 81%).

Mp.: 193-194 °C.

$[\alpha]_D^{20}$ = 12.3, c = 0.5, EtOH/$CHCl_3$ 1:1.

^{1}H-NMR (400 MHz, DMSO-d_6): δ = 0.86 (t, J = 6.7 Hz, 3H, C^6-H_3), 1.23 - 1.32 (m, 4H, C^5-H_2, C^4-H_2), 1.27 (d, J = 7.3 Hz, 3H, $C^{3'}$-H_2), 1.45 - 1.57 (m, 1H, C^3-H_2), 1.59 - 1.69 (m, 1H, C^3-H_2), 4.01 (td, J = 8.7, 5.1 Hz, 1H, C^2-H), 4.15 - 4.31 (m, 4H, $C^{2'}$-H, *Fmoc*-C*H* and *Fmoc*-CH_2), 7.29 - 7.35 (m, 2H, Ar-C*H*), 7.42 (td, J = 7.5, 1.1 Hz, 3H, C^2-N*H* and Ar-C*H*), 7.73 (t, J = 6.8 Hz, 2H, Ar-C*H*), 7.89 (d, J = 7.6 Hz, 2H, Ar-C*H*), 8.12 (d, J = 7.2 Hz, 1H, $C^{2'}$-N*H*), 12.47 (br. s., 1H, C(=O)O*H*) ppm.

^{13}C-NMR (101 MHz, DMSO-d_6): δ = 13.8 (C^6-H_3), 17.1 ($C^{3'}$-H_3), 21.8 (C^5-H_2), 27.4 (C^4-H_2), 31.7 (C^3-H_2), 46.6 and 47.3 ($C^{2'}$-H and *Fmoc*-*C*H), 54.2 (C^2-H), 65.5 (*Fmoc*-*C*H_2), 120.0 (Ar-*C*H), 125.2 (Ar-*C*H), 127.0 (Ar-*C*H), 127.6 (Ar-*C*H), 140.6 (Ar-C_{quart}), 143.7 (Ar-C_{quart}), 143.9 (Ar-C_{quart}), 155.8 (*C*(=O)), 171.7 (*C*(=O)), 173.9 (*C*(=O)OH) ppm.

HRMS (pos. ESI): Calcd for $C_{24}H_{28}N_2O_5Na$ $[M+Na]^+$: 447.1890. Found 447.1887.

Rhodium-catalyzed hydrooxycarbonylation of statine 24 to allene 25

***tert*-Butyl (3*S*,4*R*,5*S*)-4-((((9*H*-fluoren-9-yl)methoxy)carbonyl)amino)-3-((*tert*-butyldimethylsilyl)oxy)-5-methylheptanoate (24)**

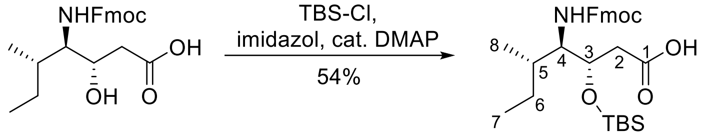

$C_{23}H_{27}NO_5$
397.47
23

$C_{29}H_{41}NO_5Si$
511.73
24

To **23**[5] (4.20 g, 10.2 mmol, 1.0 eq.) in DMF (21 ml) was added imidazole (4.33 g, 63.6 mmol, 6.0 eq.), TBSCl (4.79 g, 31.8 mmol, 3.0 eq.) and DMAP (cat.). The reaction mixture was allowed to stir for 6.5 h before again imidazole (4.33 g, 63.6 mmol, 6.0 eq.) and TBSCl (4.79 g, 31.8 mmol, 3.0 eq.) were added. The reaction mixture was allowed to stir for 20 h. MeOH (100 ml) and citric acid (25%, 150 ml) were added. The aq. phase was extracted with EA (3×150 ml). The combined organic layers were washed with water and brine and dried over Na_2SO_4. The solvent was removed under reduced pressure. The residue was purified by column chromatography (silica gel, PE:EA 4:1 – 1:3). The compound was dissolved and refluxed in hexane, and insoluble impurities were filtered off from the hot solution. Recrystallization from hexane (25 ml) delivered the desired product **24** as colorless solid (2.90 g, 5.67 mmol, 54%).

Mp.: 128 – 139 °C.

$[\alpha]_D^{20}$ = -3.0 (c = 0.50, $CHCl_3$).

^{1}H-NMR (400 MHz, DMSO-d_6): δ = 0.01 (s, 3H, Si-(C*H*$_3$)$_2$), 0.05 (s, 3H, Si-(C*H*$_3$)$_2$), 0.79 - 0.81 (m, 3H, C^8-*H*$_3$), 0.80 (s, 9H, Si-(C*H*$_3$)$_2$), 0.83 - 0.86 (m, 3H, C^7-*H*$_3$), 1.04 - 1.35 (m, 2H, C^6-*H*$_2$), 1.61 - 1.70 (m, 1H, C^5-*H*$_2$), AB-signal (δ_A =2.22, δ_B = 2.40, J_{AB} = 15.8, additional couplings J_A = 8.3 Hz, J_B = 2.7 Hz, 2H, C^2-*H*$_2$), 3.51 (ddd, *J* = 10.2, 7.1, 5.1 Hz, 1H, C^4-*H*), 4.14 (td, *J* = 8.5, 2.5 Hz, 1H, C^3-*H*), 4.21 (d, *J* = 6.8 Hz, 1H, *Fmoc*-C*H*), 4.25 - 4.31 (m, 2H, *Fmoc*-C*H*$_2$), 6.93 (d, *J* = 10.2 Hz, 1H, C^2-N*H*), 7.26 - 7.33 (m, 2H, Ar-C*H*), 7.37 - 7.44 (m, 2H, Ar-C*H*), 7.70 (dd, *J* = 7.3, 2.5 Hz, 2H, Ar-C*H*), 7.87 (d, *J* = 7.6 Hz, 2H, Ar-C*H*), 11.75 (br. s, 1H, C(=O)O*H*) ppm.

^{13}C NMR (101 MHz, DMSO-d_6): δ = -5.0 (Si-(*C*H$_3$)$_2$), -4.5 (Si-(*C*H$_3$)$_2$), 11.2 (C^7-H$_3$), 14.2 (*C*^{8}-H$_3$), 17.7 (Si-*C*(CH$_3$)$_3$), 25.7 (Si-C(*C*H$_3$)$_3$), 26.4 (*C*^{6}-H$_2$), 34.1 (*C*^{5}-H$_2$), 38.9 (*C*^{2}-H$_2$), 46.8 (*Fmoc*-*C*H), 57.8 (*C*^{4}-H), 65.2 (*Fmoc*-*C*H$_2$), 69.5 (*C*^{3}-H), 120.0 (Ar-*C*H), 125.1 (Ar-*C*H), 125.2 (Ar-*C*H), 126.9 (Ar-*C*H), 127.5 (Ar-*C*H), 140.6 (Ar-C_{quart}), 143.8 (Ar-C_{quart}), 156.5 (*C*(=O)), 172.9 (*C*(=O)O) ppm.

HRMS (pos. ESI): Calcd for $C_{29}H_{41}O_5NSiNa$ [M+Na]$^+$: 534.2646. Found 534.2647.

***tert*-Butyldimethyl(penta-3,4-dien-1-yloxy)silylether (25)**

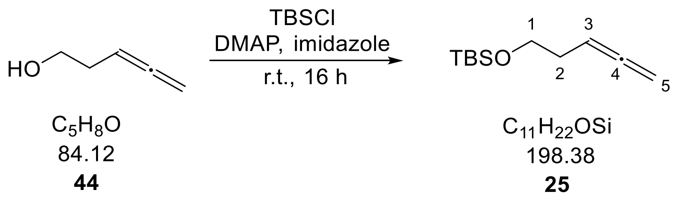

To penta-3,4-dienol[6] (**44**, 4.00 g, 47.5 mmol, 1.0 eq.), imidazole (4.87 g, 71.5 mmol, 1.5 eq.) and TBS-chloride (8.63 g, 57.3 mmol, 1.2 eq.) in DMF (24 ml) was added 4-DMAP (0.589 g, 4.82 mmol, 0.10 eq.) at r.t. The reaction mixture was allowed to stir for 16 h at this temperature before diethylether (10 ml) and water (10 ml) were added. The aq. phase was extracted with diethylether (3×40 ml). The combined org. layers were washed with brine (3×10 ml) and dried over Na_2SO_4. The solvent was removed under reduced pressure. The crude was purified by column chromatography (silica gel, pentane) to provide the desired compound **25** as colorless liquid (7.18 g, 36.2 mmol, 76%).

^{1}H-NMR (400 MHz, CDCl$_3$): δ = 0.06 (s, 6H, Si-*(CH$_3$)$_2$*), 0.90 (s, 9H, Si-C(C*H$_3$*)$_3$), 2.18 - 2.26 (m, 2H, C^2-*H$_2$*), 3.67 (t, *J* = 6.7 Hz, 2H, C^1-*H$_2$*), 4.65 (dt, *J* = 6.8, 3.0 Hz, 2H, C^5-*H$_2$*), 5.11 (quin, *J* = 6.9 Hz, 1H, C^3-*H*) ppm.

^{13}C-NMR (101 MHz, CDCl$_3$): δ = -5.2 (Si-(CH$_3$)$_2$), 18.4 (Si-C(CH$_3$)$_3$), 26.0 (Si-C(CH$_3$)$_3$), 32.1 (C^2-H$_2$), 62.9 (C^1-H$_2$), 74.5 (C^5-H$_2$), 86.8 (C^3-H), 209.2 (C^4) ppm.

HRMS (pos. APCI): Calcd for $C_{11}H_{23}OSi$ [M+H]$^+$: 199.1513. Found 199.1511.

Spectral data matched with those reported for this compound.[7]

(3*S*,4*R*,5*S*)-((*S*)-5-(*tert*-Butyldimethylsilyloxy)pent-1-en-3-yl) 4-(((9*H*-fluoren-9-yl)methoxy)carbonylamino)-3-(*tert*-butyldimethylsilyloxy)-5-methylheptanoate (26)

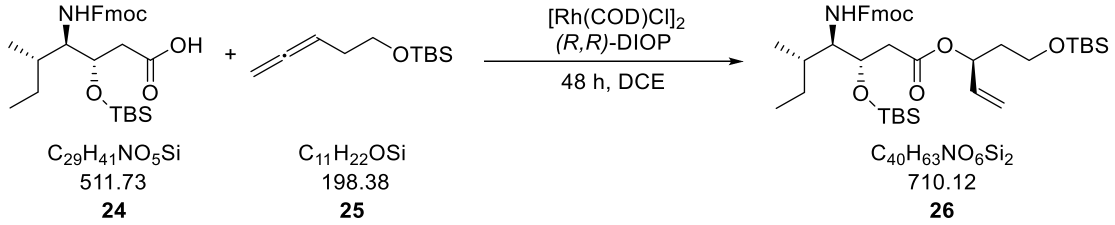

General procedure 2:

[Rh(COD)Cl]$_2$ (4.9 mg, 0.010 mmol, 4.5 mol%), *(R,R)*-DIOP (9.9 mg, 0.020 mmol, 9.0 mol%) and statine **24** (113 mg, 0.220 mmol, 1.0 eq.) were placed in a flame-dried Argon-purged Young Schlenk round-bottom flask. The flask was connected to high vacuum (4 h). Then, freshly distilled DCE (2.2 ml, 0.1 M) was added. The solution was stirred for 5 min before allene **25** (52 mg, 0.26 mmol, 1.2 eq.) filtered over basic allox was added at r.t. With addition of the allene, the color of the reaction mixture turned from orange to light yellow. The reaction was allowed to stir at 10 °C for 48 h. A crude NMR was taken to determine the *dr* from the allylic signal. The crude was purified by column chromatography (silica gel, PE:EA 95:5 – 90:10) to provide the product **26** as colorless oil (mixture of diastereomers, 119 mg, 0.168 mmol, 80%). The *dr* was determined to be 88:12 from the crude ^{1}H-NMR. A quantitative ^{13}C confirmed the ratio.

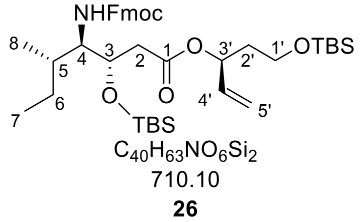

$[\alpha]_D^{20}$ = 1.28, c = 0.90, CHCl$_3$.

^{1}H-NMR (500 MHz, CDCl$_3$): d = 0.04 (s, 6H, Si-(CH_3)$_2$), 0.06 (s, 3H, Si-(CH_3)$_2$), 0.08 (s, 3H, Si-(CH_3)$_2$), 0.86 - 0.89 (m, 12H, Si-C(CH_3)$_3$ and C^8-H_3), 0.89 (s, 9H, Si-C(CH_3)$_3$), 0.92 (t, J = 7.5 Hz, 3H, C^7-H_3), 1.14 - 1.23 (m, 1H, C^6-H_2), 1.29 - 1.37 (m, 1H, C^6-H_2), 1.75 - 1.83 (m, 3H, C$^{2'}$-

H_2 and C^5-H), 1.85 - 1.95 (m, 1H, C$^{2'}$-H_2), AB-signal (δ_A = 2.50, δ_B = 2.60, J_{AB} = 16.6 Hz, additional coupling: J_A = 5.6 Hz, J_B = 6.1 Hz, 2H, C^2-H_2), 3.63 - 3.66 (m, 2H, C$^{1'}$-H_2), 3.74 (ddd, J = 10.4, 7.1, 3.0 Hz, 1H, C^4-H), 4.19 - 4.23 (m, 2H, *Fmoc*-C*H* and C^3-H), 4.32 (dd, J = 10.5, 7.0 Hz, 1H, *Fmoc*-CH_2), 4.48 (dd, J = 10.7, 7.0 Hz, 1H, *Fmoc*-CH_2), 4.75 (d, J = 10.5 Hz, 1H, C^4-NH), 5.11 (d, J = 10.5 Hz, 1H, *cis*-C$^{5'}$-H_2), 5.22 (d, J = 17.2 Hz, 1H, *trans*-C$^{5'}$-H_2), 5.33 - 5.39 (m, 1H, C$^{3'}$-H), 5.75 (ddd, J = 17.3, 10.5, 6.7 Hz, 1H, C$^{4'}$-H), 7.29 - 7.33 (m, 2H, Ar-C*H*), 7.40 (t, J = 7.5 Hz, 2H, Ar-C*H*), 7.60 (d, J = 7.5 Hz, 2H, Ar-C*H*), 7.77 (d, J = 7.6 Hz, 2H, Ar-C*H*) ppm.

^{13}C-NMR (126 MHz, CDCl$_3$): δ = -5.4 (Si-(*C*H$_3$)$_2$ (×2)), -5.0 (Si-(*C*H$_3$)$_2$), -4.4 (Si-(*C*H$_3$)$_2$), 11.8 (C^7-H$_3$), 13.9 (C^8-H$_3$), 18.0 (Si-*C*(CH$_3$)$_3$), 18.2 (Si-*C*(CH$_3$)$_3$), 25.8 (Si-C(*C*H$_3$)$_3$), 25.9 (Si-C(*C*H$_3$)$_3$), 27.5 (C^6-H$_2$), 34.3 (C^5-H$_2$), 37.2 ($C^{2'}$-H$_2$), 40.5 (C^2-H$_2$), 47.4 (*Fmoc*-*C*H), 57.7 (C^4-H), 59.0 ($C^{1'}$-H$_2$), 66.6 (*Fmoc*-*C*H$_2$), 69.9 (C^3-H), 72.7 ($C^{3'}$-H), 117.1 ($C^{5'}$-H$_2$), 119.9 (Ar-*C*H), 125.1 (Ar-*C*H), 127.0 (Ar-*C*H), 127.6 (Ar-*C*H), 136.1 ($C^{4'}$-H), 141.3 (Ar-C_{quart}), 144.0 (Ar-C_{quart}), 156.4 (*C*(=O)), 170.9 (*C*(=O)) ppm.

HRMS (pos. ESI): Calcd for $C_{40}H_{63}O_6NNaSi_2$ [M+Na]$^+$: 732.40861. Found 732.40820.

(*R*)-5-((*tert*-Butyldimethylsilyl)oxy)pent-1-en-3-yl (3*S*,4*R*,5*S*)-4-((((9*H*-fluoren-9-yl)methoxy)carbonyl)amino)-3-((*tert*-butyldimethylsilyl)oxy)-5-methylheptanoate (26)

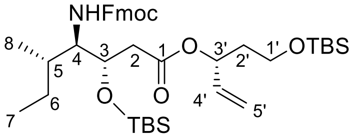

$C_{40}H_{63}NO_6Si_2$
710.10
26

The reaction was performed following the general procedure **2** using [Rh(COD)Cl]$_2$ (4.9 mg, 0.010 mmol, 4.5 mol%), *(S,S)*-DIOP (9.9 mg, 0.020 mmol, 9.0mol%), statine **24** (113 mg, 0.220 mmol, 1.0 eq.), TBS-allene **25** (52 mg, 0.26 mmol, 1.2 eq.) and DCE (2.2 ml). The reaction was run at 10 °C for 48 h. The product **26** was obtained as colorless oil (110 mg, 0.156 mmol, 71%). The *dr* was determined to be 5:95.

$[\alpha]_D^{20}$ = 11.6, c = 0.27, CHCl$_3$.

^{1}H-NMR (500 MHz, CDCl$_3$, solvent residual peak at 2.18 ppm): δ = 0.03 (s, 3H, Si-(CH_3)$_2$), 0.03 (s, 3H, Si-(CH_3)$_2$), 0.09 (s, 3H, Si-(CH_3)$_2$), 0.11 (s, 3H, Si-(CH_3)$_2$), 0.88 (s, 9H, Si-C(CH_3)$_3$), 0.90 - 0.91 (d, 3H, C^8-H_3; s, 9H, Si-C(CH_3)$_3$), 0.93 (t, J = 7.0 Hz, 3H, C^7-H_3), 1.16 - 1.25 (m, 2H, C^6-H_2), 1.58 - 1.63 (m, 1H, C^5-H), 1.75 - 1.83 (m, 1H, C$^{2'}$-H_2), 1.87 - 1.95 (m, 1H, C$^{2'}$-H_2),

AB-signal (δ_A = 2.41, δ_B = 2.54, J_{AB} = 16.6 Hz, additional coupling: J_A = 5.0 Hz, J_B = 7.3 Hz, 2H, C^2-H_2), 3.42 (t, J = 9.5 Hz, 2H, $C^{1'}$-H), 3.64 (td, J = 6.4, 1.4 Hz, 1H, C^4-H_2), 4.24 - 4.28 (m, 1H, *Fmoc*-C*H*), 4.42 (dd, J = 7.0, 2.9 Hz, 2H, *Fmoc*-C*H₂* and m, 1H, C^3-H), 4.94 (d, J = 10.2 Hz, 1H, C^4-NH), 5.17 (dt, J = 10.5, 1.2 Hz, 1H, *cis*-$C^{5'}$-H_2), 5.26 (dt, J = 17.2, 1.2 Hz, 1H, *trans*-$C^{5'}$-H_2),), 5.36 - 5.42 (m, 1H, $C^{3'}$-H), 5.79 (ddd, J = 17.2, 10.4, 6.7 Hz, 1H, $C^{4'}$-H), 7.32 (tt, J = 7.5, 1.5 Hz, 2H, Ar-CH), 7.41 (t, J = 7.5 Hz, 2H, Ar-CH), 7.61 (ddd, J = 7.5, 3.4, 0.8 Hz, 2H, Ar-CH), 7.78 (d, J = 7.6 Hz, 2H, Ar-CH) ppm.

^{13}C-NMR (126 MHz, $CDCl_3$): δ = -5.4 (Si-(CH$_3$)$_2$), -5.4 (Si-(CH$_3$)$_2$), -4.8 (Si-(CH$_3$)$_2$), -4.2 (Si-(CH$_3$)$_2$), 10.9 (C^7-H$_3$), 15.4 (C^8-H$_3$), 18.1 (Si-C(CH$_3$)$_3$), 18.2 (Si-C(CH$_3$)$_3$), 25.9 (Si-C(CH$_3$)$_3$), 25.9 (Si-C(CH$_3$)$_3$), 26.2 (C^6-H$_2$), 36.3 (C^5-H$_2$), 37.3 ($C^{2'}$-H$_2$), 40.5 (C^2-H$_2$), 47.4 (*Fmoc*-CH), 58.8 (C^4-H), 59.0 ($C^{1'}$-H$_2$), 66.5 (*Fmoc*-CH$_2$), 68.1 (C^3-H), 72.6 ($C^{3'}$-H), 117.0 ($C^{5'}$-H$_2$), 120.0 (Ar-CH), 125.1 (Ar-CH), 127.0 (Ar-CH), 127.6 (Ar-CH), 136.3 ($C^{4'}$-H), 141.4 (Ar-C_{quart}), 144.0 (Ar-C_{quart}), 144.1 (m), 156.5 (C(=O)), 170.2 (C(=O)) ppm.

HRMS (pos. ESI): Calcd for $C_{40}H_{63}O_6NNaSi_2$ [M+Na]$^+$: 732.40861. Found 732.40820.

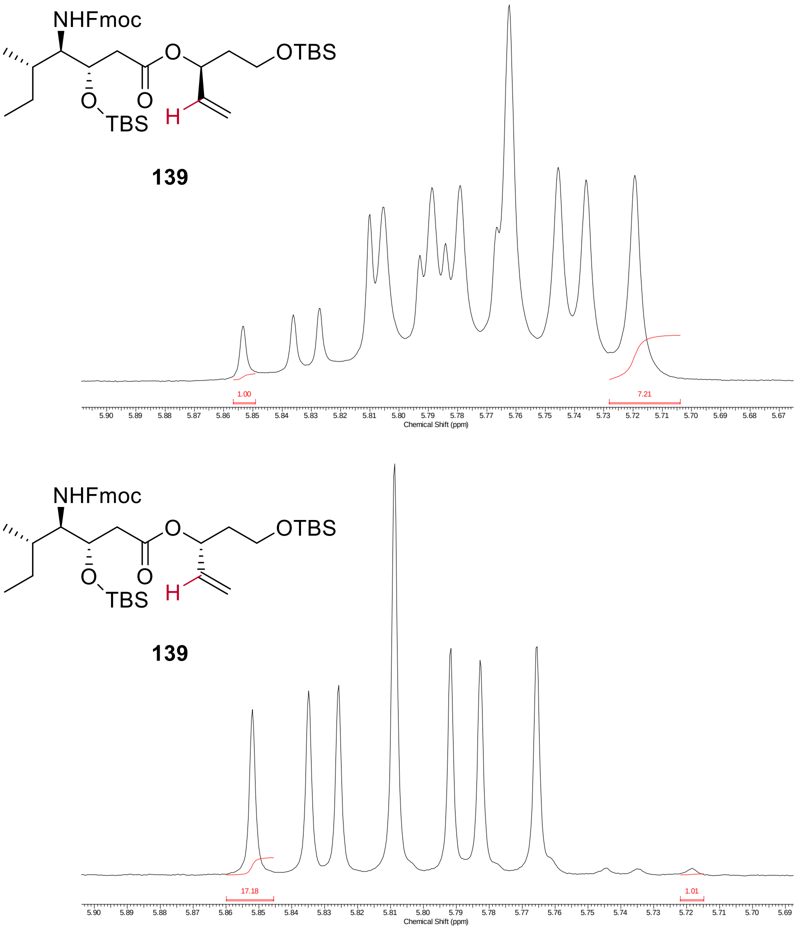

Figure 1: Signal of the highlighted proton in ^{1}H-NMR spectrum of 24.
Upper: Use of (*R*,*R*)-DIOP (*dr* 7.2:1 = 88:12). Lower: Use of (*S*,*S*)-DIOP (*dr* 1:17.2 = 5:95).

Determination of the absolute configuration of the newly formed stereocenter

(*S*)-5-((*tert*-Butyldimethylsilyl)oxy)pent-1-en-3-ol (45)

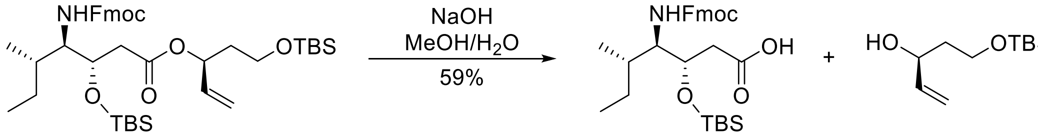

To **26** (120 mg, 0.169 mmol, 1.0 eq.) in methanol (4.5 ml) was added NaOH (350 mg, 8.75 mmol, 52 eq.) in water (1.3 ml). After 1 h at r.t. the colorless suspension had turned to a clear yellow solution. The aq. phase was extracted with pentane (3×5 ml) and DCM (2×5 ml). The combined org. layers were washed with NaCl (1×) and the solvents were removed via a Vigreux distillation apparatus at 40 °C. Due to high volatility of the desired product, the use of a rotation evaporator is not recommended. Purification by column chromatography (silica gel, pentane/Et_2O 8:1, solvent removal via Vigreux distillation apparatus at 40 °C) delivered the desired compound **45** as colorless viscous resin (22 mg, 0.102 mmol, 59%).

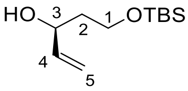

^{1}H-NMR (500 MHz, $CDCl_3$): δ = 0.08 (d, *J* = 1.2 Hz, 6H, Si-(CH_3)$_2$), 0.90 (s, 9H, Si-C(CH_3)$_3$), 1.68 - 1.83 (m, 2H, C^2-H_2), 3.32 (d, *J* = 3.4 Hz, 1H, C^3-O*H*), 3.78 - 3.83 (m, 1H, C^1-H_2), 3.86 - 3.92 (m, 1H, C^1-H_2), 4.33 - 4.38 (m, 1H, C^3-*H*), 5.11 (dt, *J* = 10.5, 1.5 Hz, 1H, *cis*-C^5-H_2), 5.28 (dt, *J* = 17.2, 1.6 Hz, 1H, *trans*-C^5-H_2), 5.88 (ddd, *J* = 17.2, 10.5, 5.4 Hz, 1H, C^4-*H*) ppm. Impurity (TBS) at δ = 0.09, 0.91 ppm.

^{13}C-NMR (126 MHz, $CDCl_3$): δ = -5.5 (Si-(*C*H_3)$_2$), -5.4 (Si-(*C*H_3)$_2$), 18.2 (Si-*C*(CH_3)$_3$), 25.9 (Si-C(*C*H_3)$_3$), 38.3 (C^2-H_2), 62.0 (C^1-H_2), 72.5 (C^3-H), 114.2 (C^5-H_2), 140.7 (C^4-H) ppm. Impurity (TBS) at δ = 3.5 (Si-(*C*H_3)$_2$), 18.1 ((Si-*C*(CH_3)$_3$), 25.7 (Si-C(*C*H_3)$_3$).

HRMS (pos. APCI): Calcd for $C_{11}H_{25}O_2Si$ $[M+H]^+$: 217.1618. Found 217.1619.

(*S*)-5-((*tert*-Butyldimethylsilyl)oxy)pent-1-en-3-yl (*R*)-3,3,3-trifluoro-2-methoxy-2-phenylpropanoate (*R*)-47

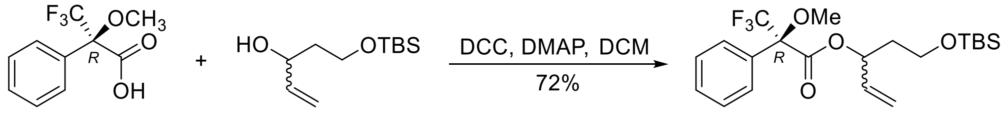

To **45** (10.9 mg, 0.0504 mmol, 1.0 eq.) in DCM (0.3 ml) were added (*R*)-Mosher's acid ((*R*)-**46**, 17.7 mg, 0.0755 mmol, 1.5 eq.) and DMAP (6.2 mg, 0.050 mmol, 1.0 eq.). The reaction mixture was allowed to stir for 10 min before DCC (15.6 mg, 0.0755 mmol, 1.5 eq.) in DCM (0.2 ml) was added. After 2.5 h at r.t. additional DMAP (3.0 mg, 0.0252 mmol, 0.5 eq.) was added. After 3 h more at r.t., the reaction was diluted with pentane (0.5 ml) and filtered over SiO_2 (2 cm) with pentane. The solvent was removed under reduced pressure. Purification by column chromatography (silica gel, pentane to pentane/Et_2O 8:1) delivered the desired compound (*R*)-**47** as colorless resin (14.5 mg, 0.0335 mmol, 72%).

$[\alpha]_D^{20}$ = 24.0, c = 0.5, DCM.

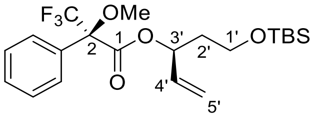

(*R,S*)-**47**/(*S,R*)-**47**:

^{1}H-NMR (500 MHz, $CDCl_3$): δ = 0.04 (s, 6H, Si-(C*H*$_3$)$_2$), 0.89 (s, 9H, Si-C(C*H*$_3$)$_3$), 1.81 - 1.89 (m, 1H, $C^{2'}$-*H*$_2$), 1.93 - 2.00 (m, 1H, $C^{2'}$-*H*$_2$), 3.54 - 3.56 (m, 3H, C^2-OC*H*$_3$), 3.64 - 3.68 (m, 2H, $C^{1'}$-*H*$_2$), 5.22 (dt, *J* = 10.4, 1.0 Hz, 1H, *cis*-$C^{5'}$-*H*$_2$), 5.29 (dt, *J* = 17.2, 1.2 Hz, 1H, *trans*-$C^{5'}$-*H*$_2$), 5.60 - 5.67 (m, 1H, $C^{3'}$-*H*), 5.76 (ddd, *J* = 17.2, 10.5, 6.9 Hz, 1H, $C^{4'}$-*H*), 7.37 - 7.42 (m, 3H, Ar-C*H*), 7.50 - 7.54 (m, 2H, Ar-C*H*) ppm.

Impurity (TBS) at δ = 0.01 (d, *J* = 3.1 Hz), 0.88 (s) ppm.

^{13}C-NMR (126 MHz, $CDCl_3$): δ = -5.3 (Si-(*C*H$_3$)$_2$), 18.3 (Si-*C*(CH$_3$)$_3$), 25.9 (Si-C(C*H*$_3$)$_3$), 37.2 (*C*$^{2'}$-H$_2$), 55.5 (C^2-O*C*H$_3$), 58.7 (*C*$^{1'}$-H$_2$), 74.8 (*C*$^{3'}$-H), 118.5 (*C*$^{5'}$-H$_2$), 123.4 (q, *J* = 289 Hz, *C*-F$_3$), 127.5 (Ar-*C*H), 128.4 (Ar-*C*H), 129.6 (Ar-*C*H), 132.4 (Ar-*C*$_{quart}$), 135.1 (*C*$^{4'}$-H), 165.8 (*C*(=O)) ppm.

HRMS (pos. APCI): Calcd for $C_{21}H_{35}F_3O_4NSi$ $[M+NH_4]^+$: 450.2282. Found 450.2289.

(*S*)-5-((*tert*-Butyldimethylsilyl)oxy)pent-1-en-3-yl (*S*)-3,3,3-trifluoro-2-methoxy-2-phenylpropanoate (*S*)-47

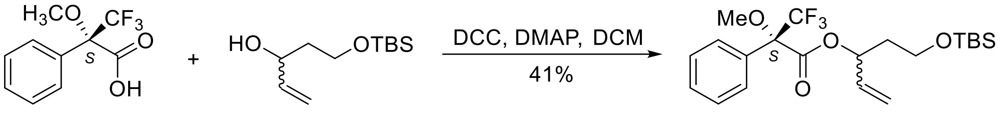

$C_{10}H_9F_3O_3$ 234.17 (*S*)-**46** | $C_{11}H_{24}O_2Si$ 216.40 **45** | $C_{21}H_{31}F_3O_4Si$ 432.56 (*S*)-**47**

45 (8.6 mg, 0.0397 mmol, 1.0 eq.), (*S*)-Mosher's acid ((*S*)-**46**, 20 mg, 0.085 mmol, 2.2 eq.), DMAP (3.5 mg, 0.0397 mmol, 1.0 eq.) and DCC (12.3 mg, 0.0595 mmol, 1.5 eq.) were placed in a flask. DCM (0.3 ml) was added and the reaction mixture was allowed to stir overnight at r.t. The reaction was diluted with pentane (0.5 ml) and filtered over SiO_2 (2 cm) with pentane. The solvent was removed under reduced pressure. Purification by column chromatography (silica gel, pentane to pentane/Et_2O 8:1) delivered the desired compound (*S*)-**47** as colorless resin (7.0 mg, 0.016 mmol, 41%).

$[\alpha]_D^{20}$ = -34.5, c = 0.4, DCM.

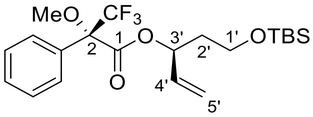

(*S*,*S*)-**47**/(*R*,*R*)-**47**:

^{1}H-NMR (500 MHz, $CDCl_3$): δ = 0.01 (d, *J* = 3.1 Hz, 6H, Si-(C*H*$_3$)$_2$), 0.88 (s, 9H, Si-C(C*H*$_3$)$_3$), 1.77 - 1.85 (m, 1H, C$^{2'}$-*H*$_2$), 1.88 - 1.96 (m, 1H, C$^{2'}$-*H*$_2$), 3.51 - 3.57 (m, 5H, C^2-OC*H*$_3$ and C$^{1'}$-*H*$_2$), 5.27 (dt, *J* = 10.4, 1.0 Hz, 1H, *cis*-C$^{5'}$-*H*$_2$), 5.38 (dt, *J* = 17.2, 1.2 Hz, 1H, *trans*-C$^{5'}$-*H*$_2$), 5.61 - 5.67 (m, 1H, C$^{3'}$-*H*), 5.86 (ddd, *J* = 17.3, 10.3, 7.2 Hz, 1H, C$^{4'}$-*H*), 7.37 - 7.40 (m, 3H, Ar-C*H*), 7.50 - 7.54 (m, 2H, Ar-C*H*) ppm.

Impurities at δ = 0.04, 0.89, 3.41 - 3.43 ppm.

^{13}C-NMR (126 MHz, $CDCl_3$): δ = -5.4 (Si-(*C*H$_3$)$_2$), 18.3 (Si-*C*(CH$_3$)$_3$), 25.9 (Si-C(C*H*$_3$)$_3$), 37.1 (*C*$^{2'}$-H$_2$s), 55.5 (C^2-O*C*H$_3$), 58.6 (*C*$^{1'}$-H$_2$), 74.9 (*C*$^{3'}$-H), 119.1 (*C*$^{5'}$-H$_2$), 123.5 (q, *J* = 289 Hz, *C*-F$_3$), 127.5 (Ar-*C*H), 128.4 (Ar-*C*H), 129.6 (Ar-*C*H), 132.6 (Ar-*C*$_{quart}$), 135.2 (*C*$^{4'}$-H), 165.8 (*C*(=O)) ppm.

HRMS (pos. APCI): Calcd for $C_{21}H_{35}F_3O_4NSi$ $[M+NH_4]^+$: 450.2282. Found 450.2289.

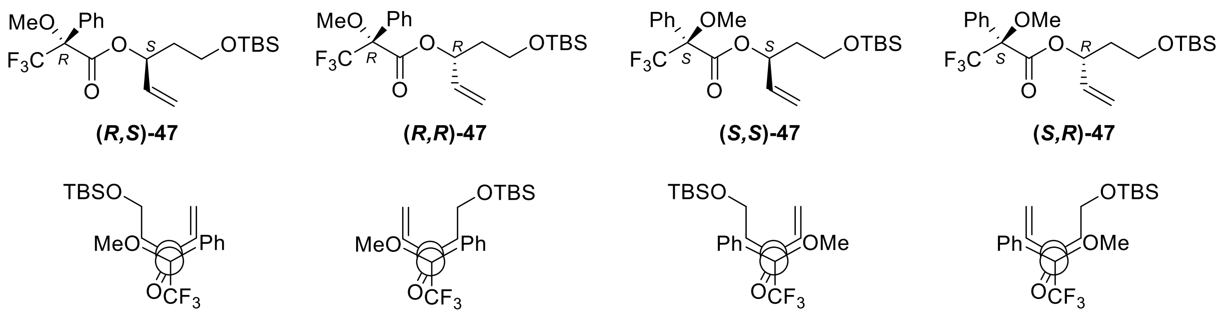

Figure 2: Possible Mosher esters and their Newman projections. For the assignment, we followed the method described in the literature.[8]

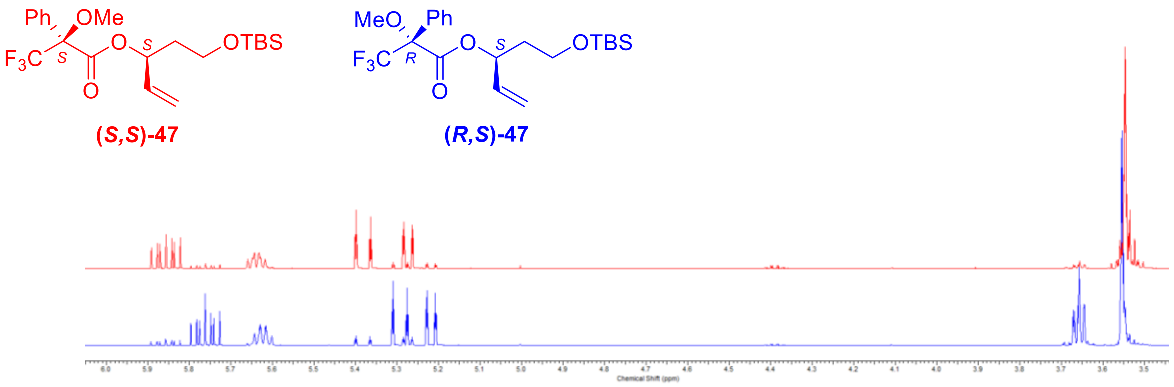

Figure 3: Comparison of ^{1}H-NMR spectra of (*S*)-47 (upper, red) and (*R*)-47 (lower, blue) for determination of the absolute configuration of the chiral allylic alcohol 44.

Coupling of Northern and Southern Part and further Conversion

(*S*)-5-((*tert*-Butyldimethylsilyl)oxy)pent-1-en-3-yl (3*S*,4*R*,5*S*)-4-amino-3-((*tert*-butyldimethylsilyl)oxy)-5-methylheptanoate (13)

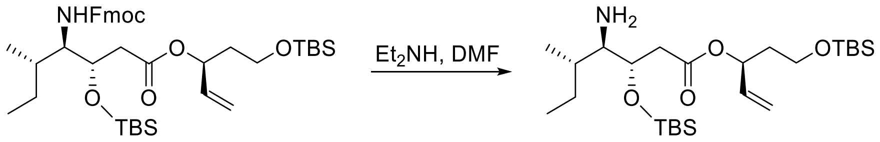

$C_{40}H_{63}NO_6Si_2$
710.10
26

$C_{25}H_{53}NO_4Si_2$
487.87
13

To **26** (19 mg, 0.030 mmol, 1.0 eq.) in DMF (1 ml) was added diethylamine (0.10 ml). After 10 min (TLC control), the deprotection was performed completely. Volatiles were removed under reduced pressure. A crude NMR and mass spectrum were taken. The crude product **13** was obtained as yellow oil and was used in the next step without further purification.

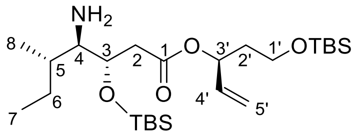

^{1}H-NMR (300 MHz, CDCl$_3$): δ = 0.02 - 0.11 (m, 12H, Si-(CH_3)$_2$ (×2)), 0.81 - 0.97 (m, 24H, Si-C(CH_3)$_3$ (×2), C^8-H_3 and C^7-H_3), 1.14 - 1.30 (m, 1H, C^6-H_2), 1.37 - 1.56 (m, 2H, C^5-H and C^6-H_2), 1.86 (dd, J = 17.7, 6.7 Hz, 2H, C$^{2'}$-H_2), 2.49 - 2.57 (m, 1H, C^2-H_2), 2.64 - 2.77 (m, 1H, C^2-H_2), 3.65 (t, J = 6.4 Hz, 1H, C$^{1'}$-H), 4.18 - 4.28 (m, 1H, C^4-H), 5.17 (d, J = 10.5 Hz, 1H, *cis*-C$^{5'}$-H_2), 5.27 (d, J = 17.2 Hz, 1H, *trans*-C$^{5'}$-H_2), 5.34 - 5.43 (m, 1H, C$^{3'}$-H), 5.80 (ddd, J = 17.2, 10.5, 6.7 Hz, 1H, C$^{4'}$-H), 6.09 (s, CH$_2$, *dibenzofulvene*), 7.28 - 7.43 (m, Ar-CH *dibenzofulvene*), 7.69 - 7.77 (m, Ar-CH *dibenzofulvene*) ppm.

HRMS (pos. ESI): Calcd for $C_{25}H_{54}NO_4Si_2$ [M+H]$^+$: 488.3586. Found 488.3587.

(*S*)-5-((*tert*-Butyldimethylsilyl)oxy)pent-1-en-3-yl (5*R*,8*R*,11*R*,12*S*)-11-((*S*)-*sec*-butyl)-5-butyl-12-((*tert*-butyldimethylsilyl)oxy)-1-(9*H*-fluoren-9-yl)-8-methyl-3,6,9-trioxo-2-oxa-4,7,10-triazatetradecan-14-oate (11)

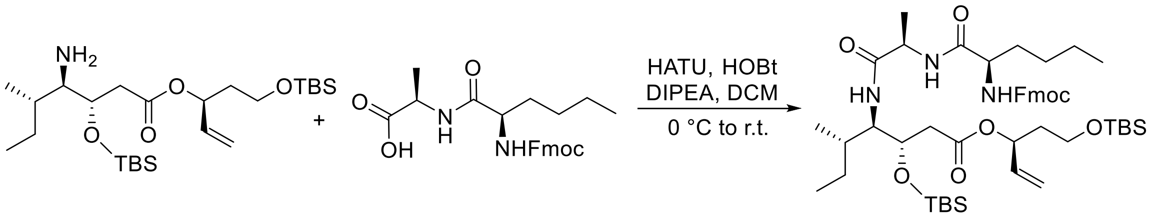

$C_{25}H_{53}NO_4Si_2$	$C_{24}H_{28}N_2O_5$	$C_{49}H_{79}N_3O_8Si_2$
487.87	424.50	894.35
**13**	**12**	**11**

To a solution of the dipeptide **12** (216 mg, 0.508 mmol, 1.4 eq.) in DCM (3.1 ml) were added HATU (220 mg, 0.581 mmol, 1.6 eq.) and HOBt (78 mg, 0.58 mmol, 1.6 eq.) at 0 °C, followed by DIPEA (198 µl, 150 mg, 1.16 mmol, 3.2 eq.). After 30 min at this temperature, the crude **13** (177 mg, 0.363 mmol, 1.0 eq.) in DCM (2.0 ml) was added. The flask was rinsed with DCM (2×0.5 ml) which was added to the reaction mixture additionally. After stirring for 16 h at r.t., the solvent was removed under reduced pressure. After column chromatography (silica gel, PE/EA 4:1 – 3:1), the desired product **11** was obtained as a colorless oil and the crude product was used in the next step without further purification.

HRMS (pos. ESI): Calcd for $C_{49}H_{79}N_3O_8Si_2Na$ [M+Na]$^+$: 916.5298. Found 916.5287.

(*S*)-5-Hydroxypent-1-en-3-yl (5*R*,8*R*,11*R*,12*S*)-11-((*S*)-*sec*-butyl)-5-butyl-12-((*tert*-butyldimethylsilyl)oxy)-1-(9*H*-fluoren-9-yl)-8-methyl-3,6,9-trioxo-2-oxa-4,7,10-triazatetradecan-14-oate (27)

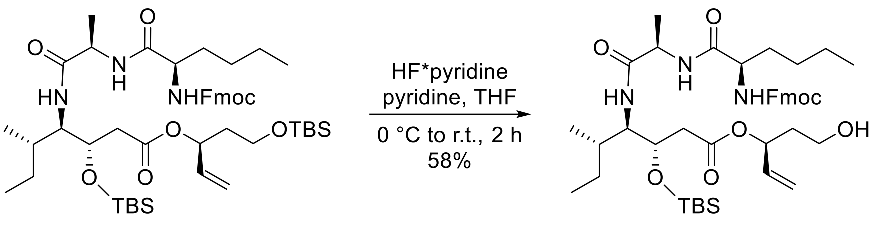

$C_{49}H_{79}N_3O_8Si_2$ 894.35 **11** → $C_{43}H_{65}N_3O_8Si$ 780.09 **27**

HF*pyridine (70%, 1.41 ml, 1.09 mg, 54.5 mmol, 150 eq.) was placed in an argon-purged 50 ml-PETP-tube with a septum and cooled to 0 °C. Pyridine (5.2 ml) was added slowly at this temperature, followed by THF (5.9 ml). The reaction mixture was allowed to come to r.t. and stirred vigorously. Then, it was again cooled down to 0 °C before **11** (325 mg, 0.363 mmol, 1.0 eq.) in THF (12.1 ml) was added at this temperature. The reaction mixture was allowed to stir for 2 h at r.t. Sat. aq. $NaHCO_3$ (52 ml) was added dropwise to the PETP-tube. Strong reaction was observed. Layers were separated and the aq. phase was extracted with EA (3×). The combined org. layers were washed with brine (1×), dried over Na_2SO_4 and the solvent was removed under reduced pressure. Purification by column chromatography (silica gel, DCM:EA:EtOH 6:1:0.1) delivered the desired product **27** as colorless oil (165 mg, 0.212 mmol, 58% combined yield of diastereomers over 3 steps from **26**). Before using in the next step, the product was dissolved in MeCN, filtrated and concentrated under reduced pressure.

$[\boldsymbol{\alpha}]_{\boldsymbol{D}}^{\mathbf{20}}$ = 37.8, c = 1.0, $CHCl_3$.

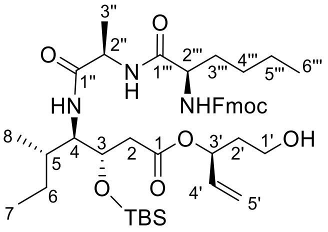

^{1}H-NMR (500 MHz, $CDCl_3$): δ = 0.09 (d, *J* = 2.1 Hz, 6H, Si-(CH_3)$_2$), 0.83 - 0.91 (m, 9H, $C^{6'''}$-H_3, C^7-H_3 and C^8-H_3), 0.88 (s, 9H, Si-C(CH_3)$_3$), 1.03 - 1.12 (m, 1H, C^6-H_2), 1.17 - 1.25 (m, 1H, C^6-H_2), 1.25 – 1.32 (m, 4H, $C^{4'''}$-H_2 and $C^{5'''}$-H_2), 1.33 (d, *J* = 6.9 Hz, 3H, $C^{3''}$-H_3), 1.54 - 1.63

(m, 1H, C$^{3'''}$-*H*$_2$), 1.72 - 1.79 (m, 1H, C$^{3'''}$-*H*$_2$), 1.80 - 1.86 (m, 1H, C^5-*H*), 1.86 - 1.97 (m, 2H, C$^{2'}$-*H*$_2$), 2.55 (d, *J* = 5.8 Hz, 2H, C^2-*H*$_2$), 3.70 - 3.75 (m, 2H, C$^{1'}$-*H*$_2$), 4.00 - 4.06 (m, 1H, C^4-*H*), 4.07-4.14 (m, 1H, C$^{2'''}$-*H*), 4.16 - 4.23 (m, 2H, *Fmoc*-C*H* and C^3-*H*), 4.33 – 4.41 (m, 2H, *Fmoc*-C*H*$_2$ and C$^{2''}$-*H*), 4.41 - 4.46 (m, 1H, *Fmoc*-C*H*$_2$), 5.16 (d, *J* = 10.5 Hz, 1H, *cis*-C$^{5'}$-*H*$_2$), 5.25 (d, *J* = 17.2 Hz, 1H, *trans*-C$^{5'}$-*H*$_2$), 5.28 - 5.33 (m, 1H, C$^{2'''}$-N*H*), 5.42 - 5.49 (m, 1H, C$^{3'}$-*H*), 5.81 (ddd, *J* = 17.1, 10.6, 6.3 Hz, 1H, C$^{4'}$-*H*), 6.51 - 6.56 (m, 1H C$^{2''}$-N*H*), 6.59 (d, *J* = 10.2 Hz, 1H, C^4-N*H*), 7.28 - 7.33 (m, 2H, Ar-C*H*), 7.40 (t, *J* = 7.5 Hz, 2H, Ar-C*H*), 7.56 - 7.60 (m, 2H, Ar-C*H*), 7.76 (d, *J* = 7.5 Hz, 2H, Ar-C*H*) ppm.

^{13}C-NMR (126 MHz, CDCl$_3$): δ = -4.8 (Si-(*C*H$_3$)$_2$), -4.3 (Si-(*C*H$_3$)$_2$), 11.9 (*C*^{7}-H$_3$), 13.8 (*C*^{8}-H$_3$), 13.9 (*C*$^{6'''}$-H$_3$), 17.2 (*C*$^{3''}$-H$_2$), 18.0 (Si-*C*(CH$_3$)$_3$), 22.4 (*C*$^{5'''}$-H$_2$), 25.8 (Si-C(*C*H$_3$)$_3$), 27.5 (*C*^{6}-H$_2$), 27.7 (*C*$^{4'''}$-H$_2$), 32.6 (*C*$^{3'''}$-H$_2$), 34.3 (*C*^{5}-H), 36.7 (*C*$^{2'}$-H$_2$), 41.7 (*C*^{2}-H$_2$), 47.3 (*Fmoc*-*C*H), 49.2 (*C*$^{2''}$-H), 55.1 (*C*$^{2'''}$-H), 56.3 (*C*^{4}-H), 58.6 (*C*$^{1'}$-H$_2$), 67.1 (*Fmoc*-*C*H$_2$), 69.4 (*C*^{3}-H), 72.6 (*C*$^{3'}$-H), 116.7 (*C*$^{5'}$-H$_2$), 120.1 (Ar-*C*H), 125.1 (Ar-*C*H), 127.2 (Ar-*C*H), 127.8 (Ar-*C*H), 136.4 (*C*$^{4'}$-H), 141.4 (Ar-*C*$_{quart}$), 143.8 (Ar-*C*$_{quart}$), 156.2 (*C*(=O)), 170.7 (*C*(=O)), 171.9 (*C*(=O)), 172.1 (*C*(=O)) ppm.

HRMS (pos. ESI): Calcd for $C_{43}H_{65}N_3O_8SiNa$ [M+Na]$^+$: 802.4433. Found 802.4430.

(5*R*,8*R*,11*R*,12*S*,16*S*)-11-((*S*)-*sec*-Butyl)-5-butyl-12-((*tert*-butyldimethylsilyl)oxy)-1-(9*H*-fluoren-9-yl)-8-methyl-3,6,9,14-tetraoxo-16-vinyl-2,15-dioxa-4,7,10-triaza-octadecan-18-oic acid (10)

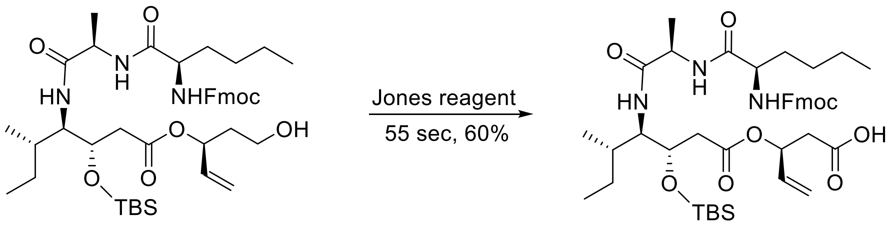

$C_{43}H_{65}N_3O_8Si$ 780.09 **27** → $C_{43}H_{63}N_3O_9Si$ 794.07 **10**

To **27** (74 mg, 0.095 mmol, 1.0 eq.) in acetone (7 ml) were added 14 drops of Jones reagent (CrO_3 in aq. H_2SO_4). After 55 sec at r.t., the reaction mixture was quenched with *i*-PrOH (9 drops) and solid $NaHCO_3$ followed by aq. $NaHCO_3$ (0.4 ml). The mixture was allowed to stir for 5 min before it was filtrated over $MgSO_4$ (3×3 cm). The $MgSO_4$ pad was rinsed with EA (30 ml). Solvents were removed under reduced pressure. Purification by column chromatography (silica gel (desact. with NEt_3, washed until neutral pH), DCM/acetone/EtOH 4:1:1, TLC developed in phosphomolybdenic acid/cerium sulfat) delivered the desired product **10** as

colorless solid (42 mg, 0.053 mmol, 60% brsm). Recovered starting material was obtained cleanly and reused in this step.

Mp.: 76 - 79 °C.

$[\alpha]_D^{20}$ = 35.9, c = 1.0, $CHCl_3$.

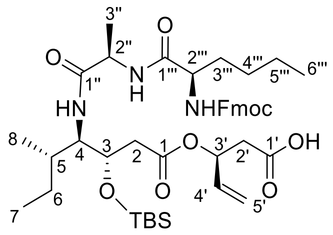

^{1}H-NMR (500 MHz, $CDCl_3$): δ = 0.09 (d, *J* = 6.4 Hz, 6H, Si-(CH_3)$_2$), 0.79 (d, *J* = 6.9 Hz, 3H, C^{8}-H_3), 0.82 - 0.92 (m, 6H, $C^{6'''}$-H_3 and C^{7}-H_3), 0.88 (s, 9H, Si-C(CH_3)$_3$), 1.00 – 1.10 (m, 1H, C^{6}-H_2), 1.14 - 1.23 (m, 1H, C^{6}-H_2), 1.23 - 1.34 (m, 11H, $C^{4'''}$-H_2 and $C^{5'''}$-H_2 and impurities), 1.38 (d, *J* = 6.0 Hz, 3H, $C^{3''}$-H_3), 1.53 - 1.64 (m, 1H, $C^{3'''}$-H_2), 1.71 - 1.79 (m, 1H, $C^{3'''}$-H_2), 1.80 - 1.87 (m, 1H, C^{5}-*H*), 2.50 - 2.56 (m, 2H, C^{2}-H_2), 2.59 - 2.71 (m, 2H, $C^{2'}$-H_2), 4.00 (t, *J* = 8.1 Hz, 1H, C^{4}-*H*), 4.11 - 4.16 (m, 1H, C^{3}-*H*), 4.16 - 4.24 (m, 2H, *Fmoc*-C*H* and $C^{2'''}$-*H*), 4.26 - 4.36 (m, 2H, *Fmoc*-CH_2 and $C^{2''}$-*H*), 4.43 (dd, *J* = 10.1, 7.3 Hz, 1H, *Fmoc*-CH_2), 5.22 (d, *J* = 10.2 Hz, 1H, *cis*-$C^{5'}$-H_2), 5.31 (d, *J* = 16.9 Hz, 1H, *trans*-$C^{5'}$-H_2), 5.45 - 5.52 (m, 1H, $C^{2'''}$-N*H*), 5.74 (d, *J* = 6.6 Hz, 1H, $C^{3'}$-*H*), 5.80 (ddd, *J* = 16.6, 10.4, 6.3 Hz, $C^{4'}$-*H*), 6.42 (d, *J* = 9.8 Hz, 1H, C^{4}-N*H*), 6.76 - 6.86 (m, 1H, $C^{2''}$-N*H*), 7.27 - 7.33 (m, 2H, Ar-C*H*), 7.39 (t, *J* = 7.2 Hz, 2H, Ar-C*H*), 7.56 (d, *J* = 6.7 Hz, 2H, Ar-C*H*), 7.76 (d, *J* = 7.3 Hz, 2H, Ar-C*H*) ppm.

^{13}C-NMR (126 MHz, $CDCl_3$): δ = -4.8 (Si-(CH_3)$_2$), -4.4 (Si-(CH_3)$_2$), 12.0 (C^{7}-H_3), 13.5 (C^{8}-H_3), 13.9 ($C^{6'''}$-H_3), 16.7 ($C^{3''}$-H_2), 17.9 (Si-*C*(CH_3)$_3$), 22.4 ($C^{5'''}$-H_2), 25.8 (Si-C(CH_3)$_3$), 27.4 (C^{6}-H_2), 27.6 ($C^{4'''}$-H_2), 32.8 ($C^{3'''}$-H_2), 34.1 (C^{5}-H), 39.4 ($C^{2'}$-H_2), 42.6 (C^{2}-H_2), 47.2 (*Fmoc-C*H), 49.6 ($C^{2''}$-H), 55.1 ($C^{2'''}$-H), 56.4 (C^{4}-H), 67.3 (*Fmoc-C*H_2), 69.7 (C^{3}-H), 71.0 ($C^{3'}$-H), 117.7 ($C^{5'}$-H_2), 120.1 (Ar-*C*H), 125.1 (Ar-*C*H), 127.2 (Ar-*C*H), 127.9 (Ar-*C*H), 134.9 ($C^{4'}$-H), 141.4 (Ar-C_{quart}), 143.8 (Ar-C_{quart}), 156.4 (*C*(=O)), 169.4 (*C*(=O)), 172.0 (*C*(=O)), 172.2 (*C*(=O)), 172.6 (*C*(=O)) ppm.

Minor impurities at δ = 1.09, 14.2, 22.8, 29.4, 29.8, 30.3, 30.4, 31.5, 32.0 ppm.

HRMS (pos. ESI): Calcd for $C_{43}H_{63}N_3O_9SiNa$ $[M+Na]^+$: 816.4226. Found 816.4219.

(*S*)-3-(((3*S*,4*R*,5*S*)-4-((*R*)-2-((*R*)-2-Aminohexanamido)propanamido)-3-((*tert*-butyldimethylsilyl)oxy)-5-methylheptanoyl)oxy)pent-4-enoic acid (48)

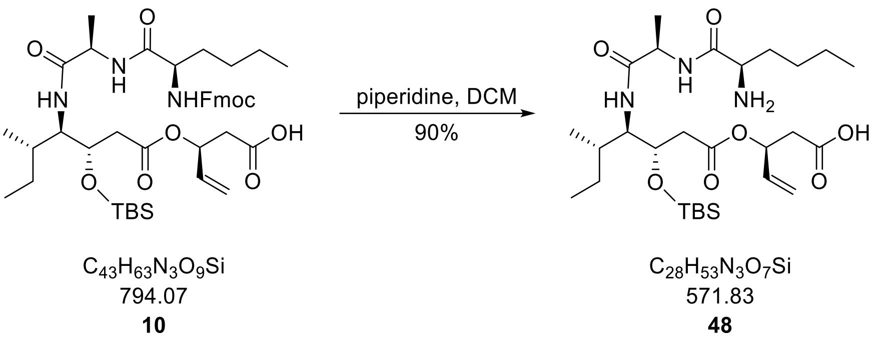

To **10** (66 mg, 83 µmol, 1.0 eq.) in DCM (1.9 ml) was added freshly distilled piperidine (0.19 ml). The reaction mixture was allowed to stir at r.t. for 45 min until the starting material was consumed completely. The solvent was removed under reduced pressure. Purification by RP column chromatography (Interchim Puriflash, C18AQ, H_2O/MeCN 100:0 – 0:100) delivered the desired product as slightly brown oil (43 mg, 75 µmol, 90%). Impurities were not removed completely. The product **48** was used in the next step without further purification.

$[\alpha]_D^{20}$ = 28.1, c = 1.0, $CHCl_3$.

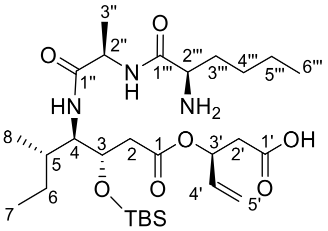

^{1}H-NMR (500 MHz, $CDCl_3$): δ = 0.07 (d, *J* = 3.5 Hz, 6H, Si-(C*H*$_3$)$_2$), 0.80 (d, *J* = 6.9 Hz, 3H, C^8-*H*$_3$), 0.84 - 0.90 (m, 6H, C$^{6'''}$-*H*$_3$ and C^7-*H*$_3$), 0.86 (s, 9H, Si-C(C*H*$_3$)$_3$), 1.00 - 1.08 (m, 1H, C^6-*H*$_2$), 1.15 - 1.24 (m, 1H, C^6-*H*$_2$), 1.25 (s, 3H, impurity), 1.27 – 1.32 (m, 4H, C$^{4'''}$-*H*$_2$ and C$^{5'''}$-*H*$_2$), 1.33 (d, *J* = 6.9 Hz, 3H, C$^{3''}$-*H*$_3$), 1.44 - 1.56 (m, 1H, C$^{3'''}$-*H*$_2$), 1.68 - 1.76 (m, 1H, C$^{3'''}$-*H*$_2$), 1.77 - 1.84 (m, 1H, C^5-*H*), 2.42 - 2.48 (m, 2H, C^2-*H*$_2$), 2.50 (d, *J* = 5.8 Hz, 2H, C$^{2'}$-*H*$_2$), 3.40 - 3.48 (m, 1H, C$^{2'''}$-*H*), 4.08 - 4.18 (m, 2H, C^3-*H* and C^4-*H*), 4.47 - 4.54 (m, 1H, C$^{2''}$-*H*), 5.10 (d, *J* = 10.5 Hz, 1H, *cis*-C$^{5'}$-*H*$_2$), 5.20 (d, *J* = 17.2 Hz, 1H, *trans*-C$^{5'}$-*H*$_2$), 5.57 - 5.62 (m, 1H, C$^{3'}$-*H*), 5.80 (ddd, *J* = 17.0, 10.8, 5.8 Hz, 1H, C$^{4'}$-*H*), 7.29 - 7.36 (m, 1H, C^4-N*H*), 7.84 - 7.91 (m, 1H, C$^{2''}$-N*H*) ppm.

^{13}C-NMR (126 MHz, CDCl$_3$): δ = -4.8 (Si-(CH$_3$)$_2$), -4.2 (Si-(CH$_3$)$_2$), 12.1 (C^7-H$_3$), 13.6 (C^8-H$_3$), 14.0 ($C^{6'''}$-H$_3$), 17.5 ($C^{3''}$-H$_2$), 17.9 (Si-C(CH$_3$)$_3$), 22.6 ($C^{5'''}$-H$_2$), 25.8 (Si-C(CH$_3$)$_3$), 27.5 (C^6-H$_2$), 27.8 ($C^{4'''}$-H$_2$), 34.2 ($C^{3'''}$-H$_2$), 34.5 (C^5-H), 41.7 ($C^{2'}$-H$_2$), 42.1 (C^2-H$_2$), 49.2 ($C^{2''}$-H), 54.8 ($C^{2'''}$-H), 56.5 (C^4-H), 68.9 (C^3-H), 72.7 ($C^{3'}$-H), 116.0 ($C^{5'}$-H$_2$), 136.3 ($C^{4'}$-H), 170.3 (C(=O)), 173.2 (C(=O)), 174.8 (C(=O)), 177.2 (C(=O)) ppm.

Impurities at δ = 1.10, 14.2, 20.8, 21.0, 21.4, 22.8, 25.3, 25.4, 29.3, 29.4, 29.6, 29.8, 31.3, 31.8, 34.1, 35.5, 40.7, 60.4, 62.1, 62.4, 69.2, 74.6, 74.7, 87.5, 126.7, 127.6, 128.5 ppm.

HRMS (pos. ESI): Calcd for $C_{28}H_{54}N_3O_7Si$ [M+H]$^+$: 572.3726. Found 572.3727.

HRMS (neg. ESI): Calcd for $C_{28}H_{52}N_3O_7Si$ [M-H]$^-$: 570.3580. Found 570.3580.

(2*S*,6*R*,9*R*,12*R*,13*S*)-12-((*S*)-*sec*-Butyl)-6-butyl-13-((*tert*-butyldimethylsilyl)oxy)-9-methyl-2-vinyl-1-oxa-5,8,11-triazacyclopentadecane-4,7,10,15-tetraone (9)

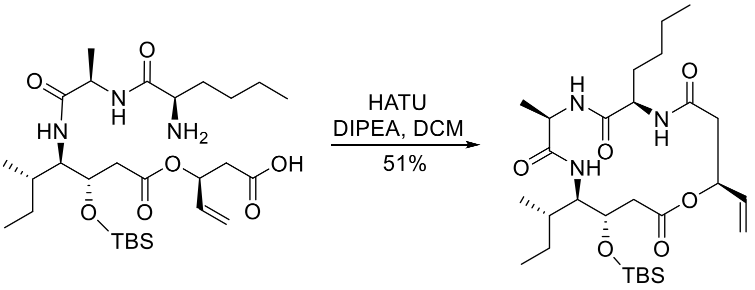

$C_{28}H_{53}N_3O_7Si$
571.83
48

$C_{28}H_{51}N_3O_6Si$
553.82
9

To a solution of HATU (85 mg, 0.22 mmol, 3.0 eq.) in DCM (37 ml) was added DIPEA (76 µl, 58 mg, 0.45 mmol, 6.0 eq.). Then, **48** (43 mg, 75 µl, 1.0 eq.) in DCM (37 ml) was added via syringe pump during 3 h. The reaction mixture was allowed to stir over night at r.t. Sat. $NaHCO_3$ was added, phases were separated and the aq. phase was extracted with DCM (3×). The combined org. layers were washed with NaCl (1×) and dried over Na_2SO_4. The solvent was removed under reduced pressure. Purification by RP column chromatography (Interchim Puriflash, C18, H_2O/MeCN 90:10 – 0:100) delivered the desired product **9** as colorless solid (21 mg, 38 µmol, 51%).

Mp.: 190 – 191 °C.

$[\alpha]_D^{20}$ = -4.9, c = 0.5, $CHCl_3$.

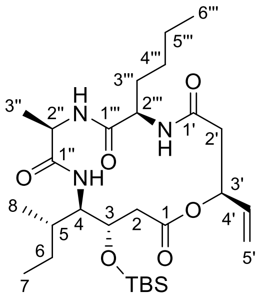

^{1}H-NMR (500 MHz, Acetone-*d*$_6$): δ = 0.07 (s, 3H, Si-(CH_3)$_2$), 0.11 (s, 3H, Si-(CH_3)$_2$), 0.88 (s, 9H, Si-C(CH_3)$_3$), 0.88 (m, 3H, C$^{6'''}$-H_3), 0.89 (d, J = 6.4 Hz, 3H, C^{8}-H_3), 0.89 (m, 3H, C^{7}-H_3), 1.17 (m, 1H, C^{6}-H_2), 1.28 (m, 1H. C^{6}-H_2), 1.29 (m, 2H, C$^{4'''}$-H_2), 1.30 (m, 2H, C$^{5'''}$-H_2), 1.55 (d, J = 7.3 Hz, 3H, C$^{3''}$-H_3), 1.69 (m, 1H, C$^{3'''}$-H_2), 1.79 (m, 1H, C$^{3'''}$-H_2), 1.95 (m, 1H, C^{5}-*H*), 2.27 (m, 1H, C^{2}-H_2), 2.51 (m, J = 15.6, 3.9 Hz, 1H, C^{2}-H_2), 2.56 (m, 1H, C$^{2'}$-H_2), 2.65 (m, 1H, C$^{2'}$-H_2), 3.82 (td, J = 10.1, 2.4 Hz, 1H, C^{4}-*H*), 3.87 (m, 1H, C$^{2''}$-*H*), 4.09 (td, J = 8.1, 6.5 Hz, 1H, C$^{2'''}$-*H*), 4.25 (dddd, J = 9.9, 6.0, 4.1, 1.8 Hz, 1H, C^{3}-*H*), 5.20 (dt, J = 10.5, 1.1 Hz, 1H, *cis*-C$^{5'}$-H_2), 5.33 (dt, J = 17.3, 1.3 Hz, 1H, *trans*-C$^{5'}$-H_2), 5.69 (m, 1H, C$^{3'}$-*H*), 5.90 (ddd, J = 17.2, 10.6, 6.3 Hz, 1H, C$^{4'}$-*H*), 7.49 (d, J = 10.2 Hz, 1H, C^{4}-N*H*), 7.81 (d, J = 7.6 Hz, 1H, C$^{2'''}$-N*H*), 7.87 (d, J = 6.9 Hz, 1H, C$^{2''}$-N*H*) ppm.

^{13}C-NMR (126 MHz, Acetone-*d*$_6$): δ = -4.6 (Si-(*C*H$_3$)$_2$), -3.5 (Si-(*C*H$_3$)$_2$), 12.4 (*C*^{7}-H$_3$), 13.8 (*C*^{8}-H$_3$), 14.2 (*C*$^{6'''}$-H$_3$), 17.2 (*C*$^{3''}$-H$_2$), 18.5 (Si-*C*(CH$_3$)), 23.0 (*C*$^{5'''}$-H$_2$), 26.2 (Si-C(*C*H$_3$)$_3$), 28.1 (*C*^{6}-H$_2$), 28.9 (*C*$^{4'''}$-H$_2$), 31.0 (*C*$^{3'''}$-H$_2$), 35.0 (*C*^{5}-H), 41.2 (*C*$^{2'}$-H$_2$), 43.5 (*C*^{2}-H$_2$), 54.7 (*C*$^{2''}$-H), 55.0 (*C*$^{2'''}$-H), 56.8 (*C*^{4}-H), 70.1 (*C*^{3}-H), 72.9 (*C*$^{3'}$-H), 117.7 (*C*$^{5'}$-H$_2$), 137.0 (*C*$^{4'}$-H), 170.3 (*C*(=O)), 170.6 (*C*(=O)), 173.4 (*C*(=O)), 173.5 (*C*(=O)) ppm.

HRMS (pos. ESI): Calcd for $C_{28}H_{51}N_3O_6SiNa$ [M+Na]$^+$: 576.3439. Found 576.3435.

(2*S*,6*R*,9*R*,12*R*,13*S*)-12-((*S*)-*sec*-Butyl)-6-butyl-13-hydroxy-9-methyl-2-vinyl-1-oxa-5,8,11-triazacyclopentadecane-4,7,10,15-tetraone (28)

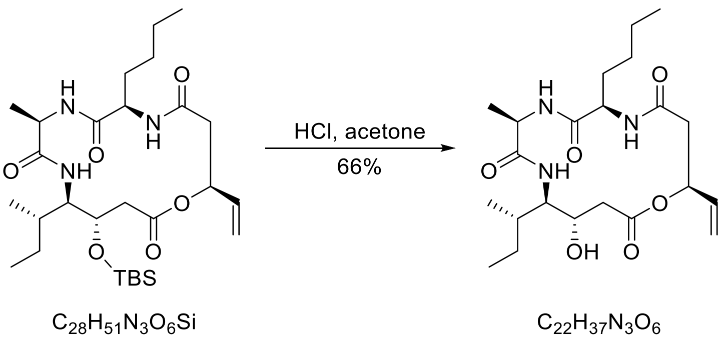

$C_{28}H_{51}N_3O_6Si$
553.82
9

$C_{22}H_{37}N_3O_6$
439.55
28

To **9** (5.0 mg, 9.0 µmol, 1.0 eq.) in acetone (0.7 ml) was added HCl (6 M, 1 drop). The reaction mixture was allowed to stir for 2.5 h at r.t. Chromatography over a pipette (SiO_2, DCM/acetone/EtOH 4:1:0.2) delivered the desired product **28** as colorless solid (2.6 mg, 5.9 µmol, 66%).

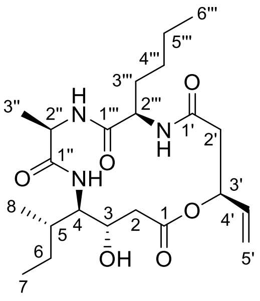

^{1}H-NMR (500 MHz, Acetone-*d*$_6$): δ = 0.83 - 0.94 (m, 12H, C^7-H_3, C^8-H_3, $C^{6'''}$-H_3), 1.19 (m, 1H, C^6-H_2), 1.28 (m, 1H, C^6-H_2), 1.26 - 1.38 (m, 4H, $C^{4'''}$-H_2 and $C^{5'''}$-H_2), 1.54 (d, *J* = 7.5 Hz, 3H, $C^{3''}$-H_3), 1.61 (m, 1H, $C^{3'''}$-H_3), 1.73 (m, 1H, $C^{3'''}$-H_3), 2.02 (m, 1H, C^5-*H*),2.23 (m, 1H, C^2-H_2), 2.46 (m, 1H, C^2-H_2), 2.57 (m, 2H, $C^{2'}$-H_2), 3.64 (m, 1H, C^4-*H*), 3.88 (quin, *J* = 7.4 Hz, 1H, $C^{2''}$-*H*), 4.09 (m, 1H, C^3-*H*), 4.24 (m, 1H, $C^{2'''}$-*H*), 5.11 (dt, *J* = 10.7, 1.4 Hz, 1H, *cis*-$C^{5'}$-H_2), 5.25 (dt, *J* = 17.3, 1.5 Hz, 1H, *trans*-$C^{5'}$-H_2), 5.57 (m, 1H, $C^{3'}$-*H*), 5.88 (ddd, *J* = 17.3, 10.7, 5.0 Hz, 1H, $C^{4'}$-*H*), 7.50 (d, *J* = 8.5 Hz, 1H, $C^{2'''}$-N*H*), 7.71 (d, *J* = 10.7 Hz, 1H, C^4-N*H*), 8.01 (d, *J* = 7.6 Hz, 1H, $C^{2''}$-N*H*) ppm.

^{13}C-NMR (126 MHz, Acetone-*d*$_6$): δ = 12.2 ($C^{6'''}$-H_3), 13.9 (C^8-H_3), 14.2 (C^7-H_3), 16.9 ($C^{3''}$-H_2), 22.9 ($C^{5'''}$-H_2), 28.0 (C^6-H_2), 28.7 ($C^{4'''}$-H_2), 32.1 ($C^{3'''}$-H_2), 34.8 (C^5-H), 41.3 ($C^{2'}$-H_2), 42.4 (C^2-H_2), 54.4 ($C^{2'''}$-H), 55.2 ($C^{2''}$-H), 56.4 (C^4-H), 69.7 (C^3-H), 72.4 ($C^{3'}$-H), 115.9 ($C^{5'}$-H_2), 137.1 ($C^{4'}$-H), 170.1 ($C^{1'}$(=O)), 171.2 (C^1(=O)), 173.3 ($C^{1''}$(=O)), 174.4 ($C^{1'''}$(=O)) ppm.

HRMS (pos. ESI): Calcd for $C_{22}H_{38}N_3O_6$ $[M+H]^+$: 440.2755. Found 440.2756.

Functionalization of the Precursor 9 towards a Thailandepsin B Alanine Derivative

(2*S*,6*R*,9*R*,12*R*,13*S*)-2-((*E*)-4-Bromobut-1-en-1-yl)-12-((*S*)-*sec*-butyl)-6-butyl-13-((*tert*-butyldimethylsilyl)oxy)-9-methyl-1-oxa-5,8,11-triazacyclopentadecane-4,7,10,15-tetraone (30)

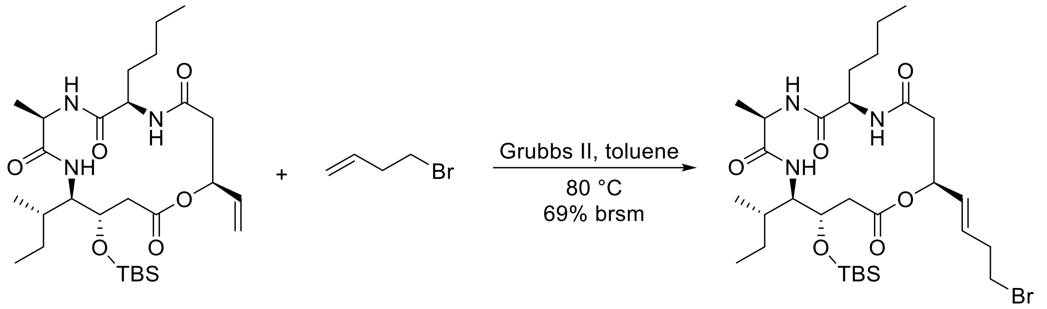

$C_{28}H_{51}N_3O_6Si$ 553.82 **9** | C_4H_7Br 135.00 **29** | $C_{30}H_{54}BrN_3O_6Si$ 660.77 **30**

To a solution of **9** (14 mg, 25 µmol, 1.0 eq.) in toluene (0.82 ml) were added Grubbs II (4.2 mg, 4.9 µmol, 0.2 eq.) and 4-bromo-1-butene (**29**, 50 µl, 67 mg, 0.49 mmol, 20 eq.). The reaction mixture was allowed to stir over night at 80 °C. Purification by RP column chromatography (Interchim Puriflash, C18, H_2O/MeCN 90:10 – 0:100) delivered the desired product **30** as colorless solid (7.4 mg, 11 µmol, 45%, 69% brsm). The starting material (4.7 mg, 8.5 µmol, 34%) was recovered purely and used again in this step.

Mp.: 208-210 °C.
$[\alpha]_D^{20}$ = -7.4, c = 0.5, $CHCl_3$.

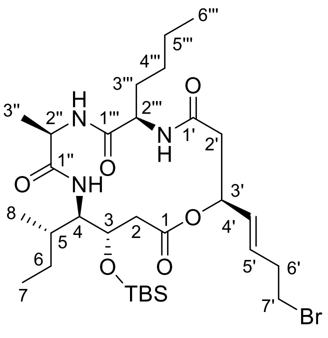

^{1}H-NMR (500 MHz, Acetone-d$_6$): δ = 0.06 (s, 3H, Si-(C*H*$_3$)$_2$), 0.12 (s, 3H, Si-(C*H*$_3$)$_2$), 0.87 (m, 3H, $C^{6'''}$-*H*$_3$), 0.87 (s, 9H, Si-C(C*H*$_3$)$_3$), 0.88 (m, 3H, C^8-*H*$_3$), 0.90 (m, 3H, C^7-*H*$_3$), 1.18 (m, 1H,

C^6-H_2), 1.26 (m, 1H, C^6-H_2), 1.33 (m, 4H, $C^{4'''}$-H_2 and $C^{5'''}$-H_2), 1.55 (d = 7.5 Hz, 3H, $C^{3''}$-H_3), 1.66 (m, 1H, $C^{3'''}$-H_2), 1.79 (m, 1H, $C^{3'''}$-H_2), 1.94 (m, 1H, C^5-*H*), 2.24 (m, 1H, C^2-H_2), 2.48 (m, 1H, C^2-H_2), 2.53 ($C^{2'}$-H_2), 2.60 (dt, 2H, $C^{6'}$-H_2), 2.68 (m, 1H, $C^{2'}$-H_2), 3.47 (t, J = 6.8 Hz, 2H, $C^{7'}$-H_2), 3.80 (m, 1H, C^4-*H*), 3.86 (m, 1H, $C^{2''}$-*H*), 4.11 (m, 1H, $C^{2'''}$-*H*), 4.21 (ddd, J = 10.0, 6.2, 3.7 Hz, 1H, C^3-*H*), 5.67 (m, 2H, $C^{3'}$-*H* and $C^{4'}$-*H*), 5.84 (m, 1H, $C^{5'}$-*H*), 7.52 (d, J = 10.4 Hz, 1H, C^4-N*H*), 7.76 (d, J = 7.6 Hz, 1H, $C^{2'''}$-N*H*), 7.89 (d, J = 6.6 Hz, 1H, $C^{2''}$-N*H*) ppm.

^{13}C-NMR (126 MHz, Acetone-d_6): δ = -4.6 (Si-(CH$_3$)$_2$), -3.4 (Si-(CH$_3$)$_2$), 12.4 (C^7-H$_3$), 13.8 (C^8-H$_3$), 14.2 ($C^{6'''}$-H$_3$), 17.2 ($C^{3''}$-H$_2$), 18.6 (Si-C(CH$_3$)), 23.0 ($C^{5'''}$-H$_2$), 26.2 (Si-C(CH$_3$)$_3$), 28.1 (C^6-H$_2$), 28.9 ($C^{4'''}$-H$_2$), 31.1 ($C^{3'''}$-H$_2$), 32.7 ($C^{7'}$-H$_2$), 34.9 (C^5-H), 36.1 ($C^{6'}$-H$_2$), 41.5 ($C^{2'}$-H$_2$), 43.5 (C^2-H$_2$), 54.9 ($C^{2''}$-H), 54.9 ($C^{2'''}$-H), 56.6 (C^4-H), 70.1 (C^3-H), 72.6 ($C^{3'}$-H), 131.5 ($C^{4'}$-H), 131.9 ($C^{5'}$-H$_2$), 170.2 ($C^{1'}$(=O)), 170.7 (C^1(=O)), 170.9 ($C^{1'''}$(=O)), 173.4 ($C^{1''}$(=O)) ppm.
Impurities at δ = 14.5 20.8, 30.6, 54.8, 60.5 ppm.

HRMS (pos. ESI): Calcd for $C_{30}H_{54}BrN_3O_6SiNa$ [M+Na]$^+$: 682.2857. Found 682.2852.

HRMS (pos. ESI): Calcd for $C_{30}H_{54}{}^{81}BrN_3O_6SiNa$ [M+Na]$^+$: 684.2837. Found 684.2833.

(2*S*,6*R*,9*R*,12*R*,13*S*)-2-((*E*)-4-Bromobut-1-en-1-yl)-12-((*S*)-*sec*-butyl)-6-butyl-13-hydroxy-9-methyl-1-oxa-5,8,11-triazacyclopentadecane-4,7,10,15-tetraone (31)

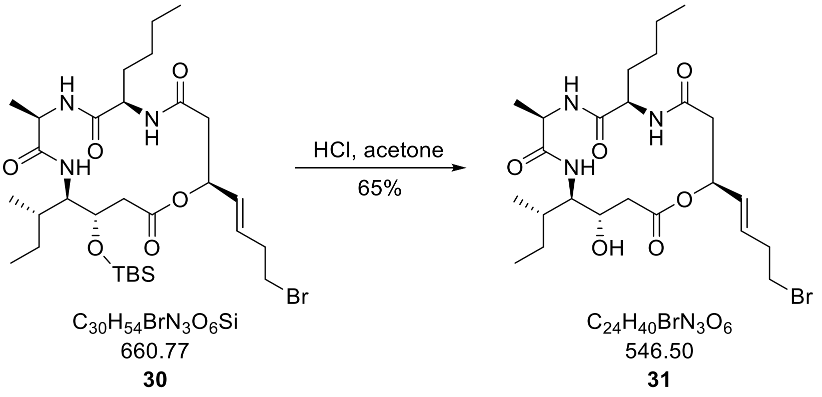

To **30** (3.7 mg, 5.6 µmol, 1.0 eq.) were added 0.4 ml of a solution of half conc. HCl in acetone (1 drop in 0.7 ml). After 2 h at r.t., solids were filtered off. Acetone (1 ml) and solid $NaHCO_3$ were added. Solids were filtered off again and the crude was dried under high vacuum. Column chromatography over a pipette (SiO_2, DCM/acetone 2:1) afforded the desired product **31** as colorless oil (2.0 mg, 3.7 µmol, 65%).

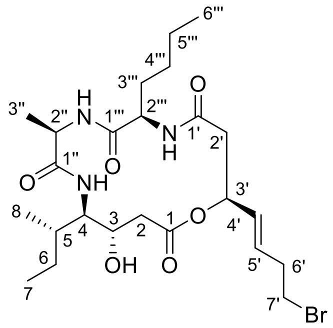

^{1}H-NMR (500 MHz, Acetone-d$_6$): δ = 0.85 - 0.91 (m, 9H, $C^{6'''}$-H_3, C^8-H_3 and C^7-H_3), 1.13 - 1.22 (m, 1H, C^6-H_2), 1.27 - 1.36 (m, 9H, C^6-H_2, $C^{4'''}$-H_2 and $C^{5'''}$-H_2), 1.52 (d, J = 7.5 Hz, 3H, $C^{3''}$-H_3), 1.57 - 1.67 (m, 1H, $C^{3'''}$-H_2), 1.70 - 1.79 (m, 1H, $C^{3'''}$-H_2), 1.98 - 2.02 (m, 1H, C^5-H), 2.18 - 2.26 (m, 1H, C^2-H_2), 2.40 - 2.54 (m, 2H, C^2-H_2 and $C^{2'}$-H_2), 2.54 - 2.68 (m, 3H, $C^{6'}$-H_2 and $C^{2'}$-H_2), 3.45 (t, J = 6.9 Hz, 2H, $C^{7'}$-H_2), 3.59 - 3.66 (m, 1H, C^4-H), 3.87 - 3.95 (m, 1H, $C^{2''}$-H), 4.07 - 4.14 (m, 1H, $C^{2'''}$-H), 4.19 - 4.27 (m, 1H, C^3-H), 5.54 - 5.60 (m, 1H, $C^{3'}$-H), 5.61 - 5.68 (m, 1H, $C^{4'}$-H), 5.70 - 5.78 (m, 1H, $C^{5'}$-H), 7.56 - 7.63 (m, 1H, C-NH), 7.64 - 7.71 (m, 1H, C-NH), 8.01 - 8.08 (m, 1H, C-NH) ppm.

HRMS (pos. ESI): Calcd for $C_{24}H_{41}BrN_3O_6$ $[M+H]^+$: 546.2173. Found 546.2179.

***S*-((*E*)-4-((2*S*,6*R*,9*R*,12*R*,13*S*)-12-((*S*)-*sec*-Butyl)-6-butyl-13-((*tert*-butyldimethyl-silyl)oxy)-9-methyl-4,7,10,15-tetraoxo-1-oxa-5,8,11-triazacyclopentadecan-2-yl)but-3-en-1-yl) ethanethioate (32)**

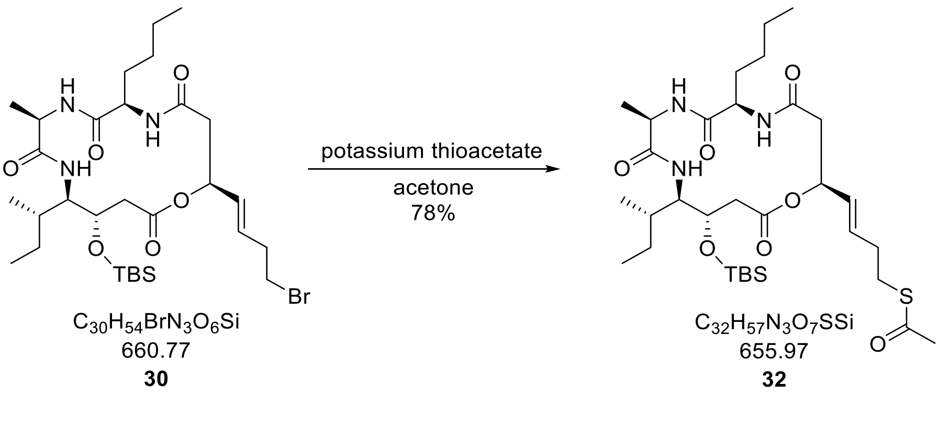

To a solution of **30** (6.8 mg, 10 µmol, 1.0 eq.) in acetone (0.4 ml) was added potassium thioacetate (5.3 mg, 46 µmol, 4.5 eq.). The reaction mixture was allowed to stir for 3 h. The solvent was removed under reduced pressure and a crude NMR was taken to confirm consumption of complete starting material. The desired product **32** was obtained by

chromatography over a pipette (SiO_2, DCM/acetone/EtOH 4:1:0.1) as colorless solid (5.3 mg, 8.1 µmol, 78%).

Mp.: 204-209 °C.

$[\boldsymbol{\alpha}]_D^{20}$ = -10.8, c = 0.26, $CHCl_3$.

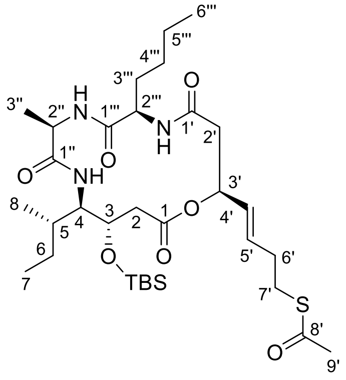

^{1}H-NMR (500 MHz, Acetone-d_6): δ = 0.07 (s, 3H, Si-(CH_3)$_2$), 0.12 (s, 3H, Si-(CH_3)$_2$), 0.88 (m, 3H, C$^{6'''}$-H_3), 0.88 (s, 9H, Si-C(CH_3)$_3$), 0.88 (m, 3H, C^{8}-H_3), 0.89 (m, 3H, C^{7}-H_3), 1.18 (m, 1H, C^{6}-H_2), 1.26 (m, 1H, C^{6}-H_2), 1.33 (m, 4H, C$^{4'''}$-H_2 and C$^{5'''}$-H_2), 1.56 (d, *J* = 7.5 Hz, 3H, C$^{3''}$-H_3), 1.66 (m, 1H, C$^{3'''}$-H_2), 1.79 (m, 1H, C$^{3'''}$-H_2), 1.95 (ddd, *J* = 14.2, 7.1, 2.4 Hz, 1H, C^{5}-*H*), 2.24 (m, 1H, C^{2}-H_2), 2.28 (m, 2H, C$^{6'}$-H_2), 2.29 (s, 3H, C$^{9'}$-H_3), 2.48 (m, 1H, C^{2}-H_2), 2.51 (m, 1H, C$^{2'}$-H_2), 2.66 (m, 1H, C$^{2'}$-H_2), 2.90 (m, 2H, C$^{7'}$-H_2), 3.79 (m, 1H, C^{4}-*H*), 3.84 (m, 1H, C$^{2''}$-*H*), 4.11 (m, 1H, C$^{2'''}$-*H*), 4.19 (td, *J* = 6.6, 3.1 Hz, 1H, C^{3}-*H*), 5.60 (m, 1H, C$^{4'}$-*H*), 5.66 (m, 1H, C$^{3'}$-*H*), 5.81 (m, 1H, C$^{5'}$-*H*), 7.53 (d, *J* = 10.4 Hz,, 1H, C^{4}-N*H*), 7.71 (d, *J* = 7.9 Hz, 1H, C$^{2'''}$-N*H*), 7.87 (d, *J* = 6.9 Hz, 1H, C$^{2''}$-N*H*) ppm.

^{13}C-NMR (126 MHz, Acetone-d_6): δ = -4.6 (Si-(*C*H_3)$_2$), -3.4 (Si-(*C*H_3)$_2$), 12.4 (*C*^{7}-H_3), 13.8 (*C*^{8}-H_3), 14.2 (*C*$^{6'''}$-H_3), 17.1 (*C*$^{3''}$-H_2), 18.6 (Si-*C*(CH_3)$_3$), 23.0 (*C*$^{5'''}$-H_2), 26.2 ((Si-C(*C*H_3)$_3$), 28.1 (*C*^{6}-H_2), 28.6 (*C*$^{7'}$-H_2), 28.9 (*C*$^{4'''}$-H_2), 30.5 (*C*$^{9'}$-H_3), 31.1 (*C*$^{3'''}$-H_2), 33.0 (*C*$^{6'}$-H_2), 34.9 (*C*^{5}-H), 41.5 (*C*$^{2'}$-H_2), 43.5 (*C*^{2}-H_2), 54.8 (*C*$^{2'''}$-H), 55.0 (*C*$^{2''}$-H), 56.6 (*C*^{4}-H), 70.1 (*C*^{3}-H), 72.7 (*C*$^{3'}$-H), 130.5 (*C*$^{4'}$-H), 132.9 (*C*$^{5'}$-H_2), 170.2 (*C*$^{1'}$(=O)), 170.6 (*C*^{1}(=O)), 173.4 (*C*$^{1''}$(=O)), 173.8 (*C*$^{1'''}$(=O)), 195.2 (*C*$^{8'}$(=O)) ppm.

HRMS (pos. ESI): Calcd for $C_{32}H_{58}N_3O_7SSi$ $[M+H]^+$: 656.3770. Found 656.3753.

***S*-((*E*)-4-((2*S*,6*R*,9*R*,12*R*,13*S*)-12-((*S*)-*sec*-Butyl)-6-butyl-13-hydroxy-9-methyl-4,7,10,15-tetraoxo-1-oxa-5,8,11-triazacyclopentadecan-2-yl)but-3-en-1-yl) ethanethioate (33)**

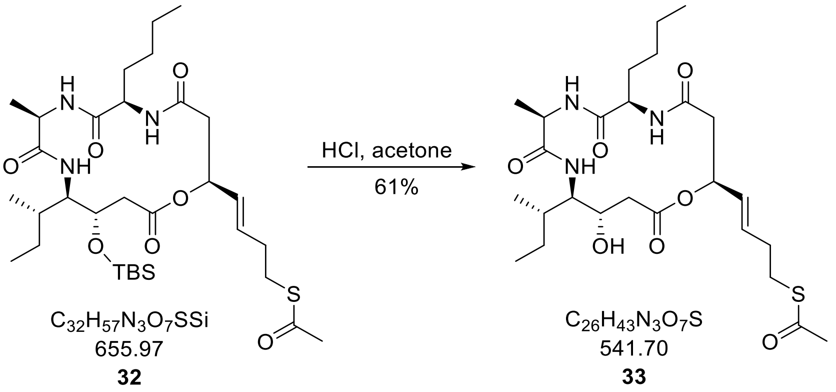

To a solution of **32** (3.0 mg, 4.6 µmol, 1.0 eq.) in acetone (0.5 ml) was added HCl (half conc., 1 drop). The reaction mixture was allowed to stir for 3.5 h before solvents were removed under reduced pressure. Chromatography over a pipette (SiO_2, DCM/acetone 2:1 – 1:1) delivered the desired product **33** as colorless oil (1.5 mg, 2.8 µmol, 61%).

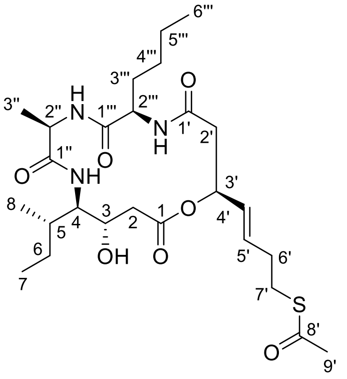

^{1}H-NMR (500 MHz, Acetone-*d*$_6$): δ = 0.88 (m, 3H, $C^{6'''}$-H_3), 0.89 (m, 3H, C^7-H_3), 0.90 (m, 3H, C^8-H_3), 1.18 (m, 1H, C^6-H_2), 1.26 - 1.38 (m, 5H, C^6-H_2, $C^{4'''}$-H_2 and $C^{5'''}$-H_2), 1.52 (d, J = 7.5 Hz, 3H, $C^{3''}$-H_3), 1.62 (m, 1H, $C^{3'''}$-H_2), 1.75 (m, 1H, $C^{3'''}$-H_2), 2.00 (m, 1H, C^5-H), 2.20 (m, 1H, C^2-H_2), 2.25 (m, 2H, $C^{6'}$-H_2), 2.29 (s, 3H, $C^{9'}$-H_3), 2.44 (m, 1H, C^2-H_2), 2.49 (m, 2H, $C^{2'}$-H_2), 2.62 (m, 1H, $C^{2'}$-H_2), 2.88 (t, J = 7.8 Hz, 2H, $C^{7'}$-H_2), 3.63 (m, 1H, C^4-H), 3.90 (m, 1H, $C^{2''}$-H), 4.09 (m, 1H, C^3-H), 4.22 (m, 1H, $C^{2'''}$-H), 5.55 (m, 2H, $C^{3'}$-H), 5.56 (m, 2H, $C^{4'}$-H), 5.71 (m, 1H, $C^{5'}$-H), 7.61 (m, 1H, $C^{2'''}$-NH), 7.68 (d, J = 9.8 Hz, 1H, C^4-NH), 8.04 (m, 1H, $C^{2''}$-NH) ppm.

^{13}C-NMR (126 MHz, Acetone-*d*$_6$): δ = 12.2 (C^7-H_3), 14.0 (C^8-H_3), 14.2 ($C^{6'''}$-H_3), 16.9 ($C^{3''}$-H_2), 22.9 ($C^{5'''}$-H_2), 28.1 (C^6-H_2), 28.7 ($C^{4'''}$-H_2), 28.8 ($C^{7'}$-H_2), 30.5 ($C^{9'}$-H_3), 32.1 ($C^{3'''}$-H_2), 33.0 ($C^{6'}$-H_2), 34.9 (C^5-H), 41.7 ($C^{2'}$-H_2), 42.5 (C^2-H_2), 54.6 ($C^{2'''}$-H), 54.9 ($C^{2''}$-H), 56.4 (C^4-H), 69.5 (C^3-

H), 72.1 ($C^{3'}$-H), 130.8 ($C^{4'}$-H), 130.9 ($C^{5'}$-H_2), 170.2 ($C^{1'}$(=O)), 171.3 (C^{1}(=O)), 173.3 ($C^{1''}$(=O)), 174.2 ($C^{1'''}$(=O)), 195.5 ($C^{8'}$(=O)) ppm.

HRMS (pos. ESI): Calcd for $C_{26}H_{44}N_3O_7S$ $[M+H]^+$: 542.2905. Found 542.2897.

Functionalization of the Precursor 9 towards a Thailandepsin B Alanine Derivative with Hydroxamic acid Warhead

***tert*-Butyl ((*tert*-butoxycarbonyl)oxy)((*E*)-5-((2*S*,6*R*,9*R*,12*R*,13*S*)-12-((*S*)-*sec*-butyl)-6-butyl-13-((*tert*-butyldimethylsilyl)oxy)-9-methyl-4,7,10,15-tetraoxo-1-oxa-5,8,11-triazacyclopentadecan-2-yl)pent-4-enoyl)carbamate (35)**

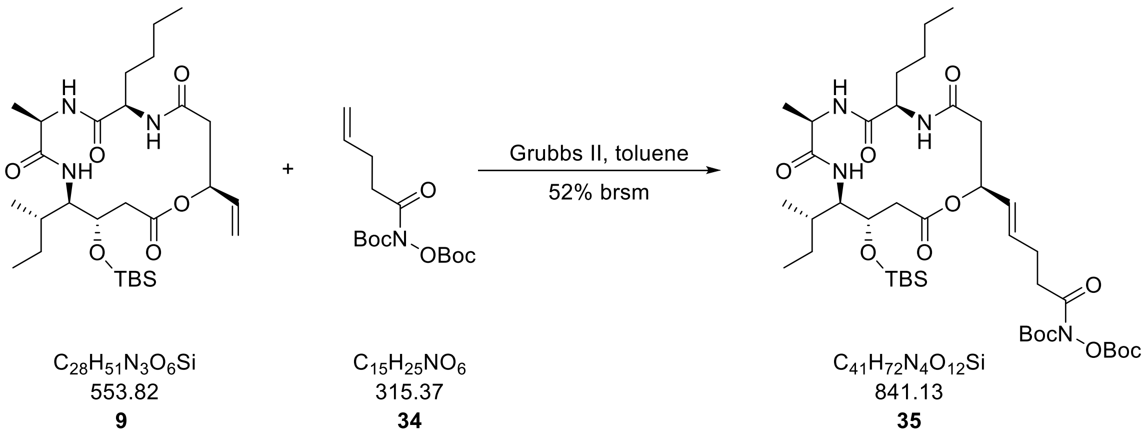

$C_{28}H_{51}N_3O_6Si$ 553.82 **9**; $C_{15}H_{25}NO_6$ 315.37 **34**; $C_{41}H_{72}N_4O_{12}Si$ 841.13 **35**

Compound **9** (6.3 mg, 0.0114 mmol, 1.0 eq.) and Grubbs II (1.9 mg, 2.3 µmol, 0.2 eq.) were placed in a 1 ml round bottom flask with a YOUNG cap. Toluene (0.2 ml) and **34**[9] (38 mg, 0.12 mmol, 10 eq.) were added successively under Argon. The reaction mixture was allowed to stir for 18 h at 80 °C. The solvent was removed under reduced pressure and the crude product was purified by RP column chromatography (Interchim Puriflash, C18, H_2O/MeCN 90:10 – 0:100) followed by column chromatography over a pipette (SiO_2, DCM/acetone 3:1). The desired product **35** was obtained as colorless solid (2.4 mg, 2.9 µmol, 25%, brsm 52%). The starting material (3.3 mg, 6.0 µmol, 52%) was recovered purely and used again in this step.

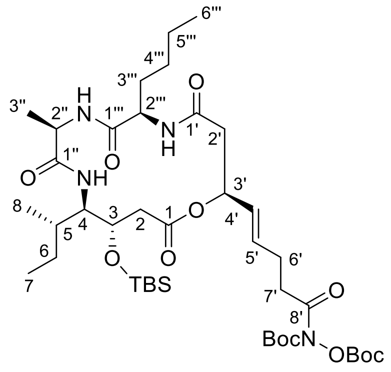

^{1}H-NMR (500 MHz, Acetone-*d_6*): 0.07 (s, 3H, Si-(CH_3)$_2$), 0.12 (s, 3H, Si-(CH_3)$_2$), 0.88 (m, 3H, $C^{6'''}$-H_3), 0.88 (s, 9H, Si-C(CH_3)$_3$), 0.88 (m, 3H, C^{8}-H_3), 0.89 (m, 3H, C^{7}-H_3), 1.16 (m, 1H, C^{6}-H_2), 1.28 (m, 1H, C^{6}-H_2), 1.30 – 1.35 (m, 4H, $C^{4'''}$-H_2 and $C^{5'''}$-H_2), 1.52 (m, 18H, *Boc*), 1.55 (d, J = 7.3 Hz, 3H, $C^{3''}$-H_3), 1.66 (m, 1H, $C^{3'''}$-H_2), 1.78 (m, 1H, $C^{3'''}$-H_2), 1.95 (ddd, J = 13.9, 6.9, 2.3 Hz, 1H, C^{5}-H), 2.22 (m, 1H, C^{2}-H_2), 2.37 (m, 2H, $C^{6'}$-H_2), 2.48 (m, 1H, C^{2}-H_2), 2.51 (m, 1H, $C^{2'}$-H_2), 2.68 (m, 1H, $C^{2'}$-H_2), 2.93 (m, 2H, $C^{7'}$-H_2), 3.80 (td, J = 10.3, 2.4 Hz, 1H, C^{4}-H), 3.87 (quin, J = 7.3 Hz, 1H, $C^{2''}$-H), 4.13 (m, 1H, $C^{2'''}$-H), 4.20 (ddd, J = 9.9, 6.4, 3.3 Hz, 1H, C^{3}-H), 5.60 (m, 1H, $C^{4'}$-H), 5.66 (m, 1H, $C^{3'}$-H), 5.90 (dt, J = 15.0, 6.8 Hz, 1H, $C^{5'}$-H), 7.58 (d, J = 10.4 Hz, 1H, C^{4}-NH), 7.76 (d, J = 5.5 Hz, 1H, $C^{2'''}$-NH), 7.95 (d, J = 6.7 Hz, 1H, $C^{2''}$-NH) ppm.

^{13}C-NMR (126 MHz, Acetone-*d_6*): δ = -4.7 (Si-(CH_3)$_2$), -3.3 (Si-(CH_3)$_2$), 12.4 (C^{7}-H_3), 13.8 (C^{8}-H_3), 14.2 ($C^{6'''}$-H_3), 17.3 ($C^{3''}$-H_2), 18.6 ((Si-C(CH_3)$_3$), 23.0 ($C^{5'''}$-H_2), 26.3 (Si-C(CH_3)$_3$), 27.6 (*Boc*-C(CH)$_3$), 28.0 (*Boc*-C(CH)$_3$), 28.1 (C^{6}-H_2), 28.9 ($C^{4'''}$-H_2), 31.2 ($C^{3'''}$-H_2), 34.9 (C^{5}-H), 36.8 ($C^{7'}$-H_2), 41.5 ($C^{2'}$-H_2), 43.5 (C^{2}-H_2), 54.8 ($C^{2'''}$-H), 55.0 ($C^{2''}$-H), 56.5 (C^{4}-H), 70.2 (C^{3}-H), 72.8 ($C^{3'}$-H), 85.9 (*Boc*-C(CH_3)), 86.5 (*Boc*-C(CH_3)), 129.8 ($C^{4'}$-H), 130.3 ($C^{5'}$-H_2), 150.5 (*Boc*-C(=O)), 169.5 ($C^{8'}$(=O)), 170.2 ($C^{1'}$(=O)), 170.7 (C^{1}(=O)), 173.5 ($C^{1''}$(=O)), 173.7 ($C^{1'''}$(=O)) ppm.

HRMS (pos. ESI): Calcd for $C_{41}H_{72}N_4O_{12}SiNa$ $[M+Na]^+$: 863.4808. Found 863.4797.

***tert*-Butyl ((*tert*-butoxycarbonyl)oxy)((*E*)-5-((2*S*,6*R*,9*R*,12*R*,13*S*)-12-((*S*)-*sec*-butyl)-6-butyl-13-hydroxy-9-methyl-4,7,10,15-tetraoxo-1-oxa-5,8,11-triazacyclopenta-decan-2-yl)pent-4-enoyl)carbamate (36)**

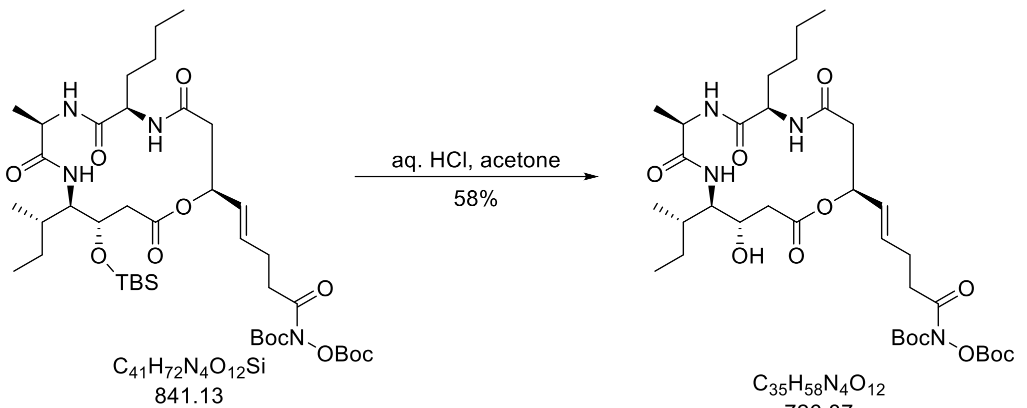

To **35** (4.0 mg, 4.8 µmol, 1.0 eq.) were added 0.3 ml of a solution of half conc. HCl in acetone (1 drop in 0.7 ml). After 2.5 h at r.t., solids were filtered off. Solvents were coevaporated with toluene (1×). Acetone and solid $NaHCO_3$ were added. Solids were filtered off and the crude product was dried on high vacuum. Column chromatography over a pipette (SiO_2, DCM/acetone 4:1 – 2:1) afforded the desired product **36** as colorless oil (2.0 mg, 2.8 µmol, 58%).

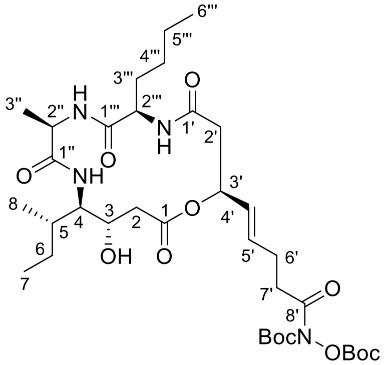

^{1}H-NMR (500 MHz, Acetone-*d*$_6$): 0.85 - 0.91 (m, 9H, $C^{6''''}$-H_3, C^8-H_3 and C^7-H_3), 1.15 - 1.18 (m, 1H, C^6-H_2), 1.30 - 1.38 (m, 9H, C^6-H_2, $C^{4''''}$-H_2 and $C^{5''''}$-H_2), 1.51 - 1.54 (m, 21H, *Boc* and $C^{3''}$-H_3), 1.59 - 1.67 (m, 1H, $C^{3''''}$-H_2), 1.71 - 1.78 (m, 1H, $C^{3''''}$-H_2), 1.99 - 2.03 (m, 1H, C^5-*H*), 2.19 - 2.26 (m, 1H, C^2-H_2), 2.32 - 2.38 (m, 2H, $C^{6'}$-H_2), 2.41 - 2.46 (m, 1H, C^2-H_2), 2.48 - 2.52 (m, 1H, $C^{2'}$-H_2), 2.58 - 2.64 (m, 1H, $C^{2'}$-H_2), 2.90 - 2.95 (m, 2H, $C^{7'}$-H_2), 3.59 - 3.66 (m, 1H, C^4-*H*), 3.86 - 3.93 (m, 1H, $C^{2''}$-*H*), 4.04 - 4.14 (m, 1H, $C^{2''''}$-*H*), 4.17 - 4.24 (m, 1H, C^3-*H*), 5.53 - 5.61 (m, 2H, $C^{4'}$-*H* and $C^{3'}$-*H*), 5.75 - 5.84 (m, 1H, $C^{5'}$-*H*), 7.52 - 7.59 (m, 1H, C^4-N*H*), 7.62 - 7.68 (m, 1H, $C^{2''''}$-N*H*), 7.90 - 8.02 (m, 1H, $C^{2''}$-N*H*) ppm.

^{13}C-NMR (126 MHz, Acetone-d_6): δ = 12.2 (C^{7}-H$_3$), 14.0 (C^{8}-H$_3$), 14.2 ($C^{6'''}$-H$_3$), 16.9 ($C^{3''}$-H$_2$), 22.9 ($C^{5'''}$-H$_2$), 27.6 (*Boc*-C(*C*H)$_3$), 28.0 (*Boc*-C(*C*H)$_3$), 28.1 (C^{6}-H$_2$), 28.8 ($C^{4'''}$-H$_2$), 32.0 ($C^{3'''}$-H$_2$), 34.9 (C^{5}-H), 36.9 ($C^{7'}$-H$_2$), 41.7 ($C^{2'}$-H$_2$), 42.5 (C^{2}-H$_2$), 54.6 ($C^{2'''}$-H), 54.9 ($C^{2''}$-H), 56.6 (C^{4}-H), 69.5 (C^{3}-H), 72.1 ($C^{3'}$-H), 85.9 (*Boc*-*C*(CH)$_3$), 86.5 (*Boc*-*C*(CH)$_3$), 130.0 ($C^{4'}$-H), 131.7 ($C^{5'}$-H$_2$), 150.5 (*Boc*-*C*(=O)), 169.5 ($C^{8'}$(=O)), 170.2 ($C^{1'}$(=O)), 171.3 (C^{1}(=O)), 173.3 ($C^{1''}$(=O)), 174.2 ($C^{1'''}$(=O)) ppm.

HRMS (pos. ESI): Calcd for $C_{35}H_{59}N_4O_{12}$ [M+H]$^+$: 727.4124. Found 727.4116.

(*E*)-5-((2*S*,6*R*,9*R*,12*R*,13*S*)-12-((*S*)-*sec*-Butyl)-6-butyl-13-hydroxy-9-methyl-4,7,10,15-tetraoxo-1-oxa-5,8,11-triazacyclopentadecan-2-yl)-N-hydroxypent-4-enamide (8)

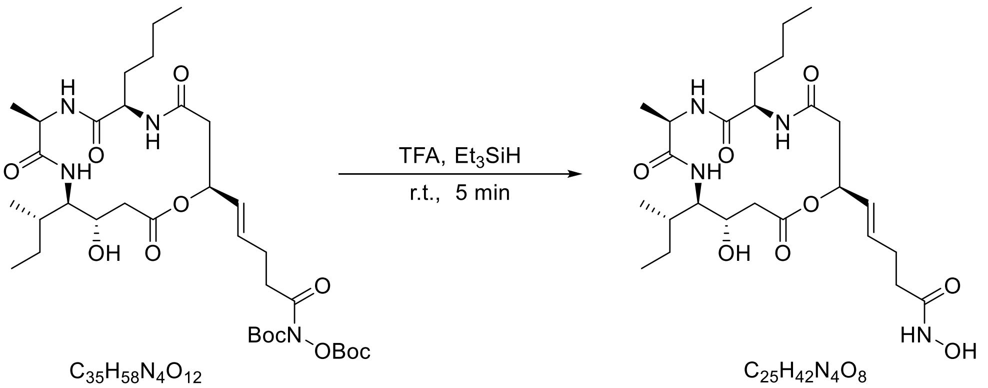

$C_{35}H_{58}N_4O_{12}$
726.87
36

$C_{25}H_{42}N_4O_8$
526.63
8

To **36** (2.0 mg, 2.8 µmol, 1.0 eq.) were added 0.03 ml of a solution of triethylsilane (13 µmol, 0.47 eq.) in DCM (1.0 mg Et$_3$SiH in 0.2 ml DCM) followed by TFA (0.2 ml, 0.3 mg, 2.6 mmol). After 5 min, full conversion was observed. Solids were filtered off. Acetone (0.5 ml) and solid NaHCO$_3$ were added. Solids were filtered off and the crude product was dried on high vacuum. The crude product **8** was obtained as colorless foam (1.8 mg, quant.).

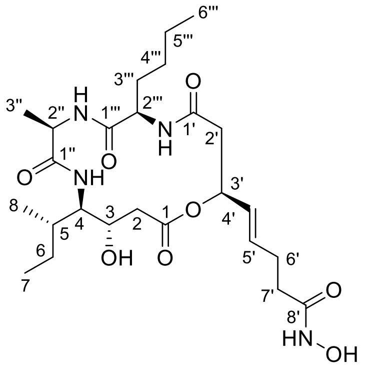

^{1}H-NMR (500 MHz, Acetone-d_6): 0.84 - 0.93 (m, 9H, C$^{6'''}$-*H$_3$*, C^{8}-*H$_3$* and C^{7}-*H$_3$*), 1.12 - 1.22 (m, 1H, C^{6}-*H$_2$*), 1.30 - 1.35 (m, 9H, C^{6}-*H$_2$*, C$^{4'''}$-*H$_2$* and C$^{5'''}$-*H$_2$*), 1.49 (d, J = 7.3 Hz, 3H, C$^{3''}$-*H$_3$*),

1.55 - 1.66 (m, 1H, $C^{3'''}$-H_2), 1.69 - 1.77 (m, 1H, $C^{3'''}$-H_2), 1.98 - 2.02 (m, 1H, C^{5}-H), 2.12 - 2.19 (m, 1H, C^{2}-H_2), 2.26 - 2.32 (m, 2H, $C^{6'}$-H_2), 2.40 - 2.46 (m, 1H, C^{2}-H_2), 2.58 - 2.68 (m, 1H, $C^{2'}$-H_2), 2.90 - 2.95 (m, 2H, $C^{7'}$-H_2), 3.63 - 3.71 (m, 1H, C^{4}-H), 3.98 - 4.05 (m, 1H, $C^{2''}$-H), 4.06 - 4.12 (m, 1H, $C^{2'''}$-H), 4.20 - 4.31 (m, 1H, C^{3}-H), 5.47 - 5.57 (m, 2H, $C^{4'}$-H and $C^{3'}$-H), 5.69 - 5.81 (m, 1H, $C^{5'}$-H), 7.63 (d, J = 9.8 Hz, 1H, C-NH), 7.84 - 7.95 (m, 1H, C-NH), 8.16 - 8.25 (m, 1H, C-NH) ppm.

HRMS (pos. ESI): Calcd for $C_{25}H_{42}N_4O_8Na$ $[M+Na]^+$: 549.2895. Found 549.2891.

Assay Methods

In vitro assays

hHDAC1/6: Activity assays were performed in OptiPlate^{TM}-96 F black microplates (PerkinElmer). Commercial available enzymes (human recombinant HDAC1; BPS Bioscience, catalog no. 50051 and human recombinant HDAC6; BPS Bioscience, catalog no. 50006) were used. Total assay volume of 60 µL contains 42 µl of assay buffer (50 mM Tris-HCl, pH 8.0, 137 mM NaCl, 2.7 mM KCl, 1 mM $MgCl_2$, 0.2 mM TCEP and 1 mg/mL bovine serum albumin), 10 µL of enzyme solution in assay buffer, 3 µL of increasing concentrations of inhibitors in DMSO and 5 µL of the fluorogenic substrate ZMAL (Z-(Ac)Lys-AMC) (126 µM). After incubation (90 min, 37 °C) 60 µL of stop solution, containing 5 µL Trichostatin A (TSA) (33 µM) and 10 µL trypsin (6 mg/mL) in trypsin buffer (Tris-HCl 50 mM, pH 8.0, NaCl 100 mM), were added. After incubation (30 min at 37 °C) fluorescence signal (λ_{ex} = 390 nm, λ_{em} = 460 nm) was measured on a BMG LABTECH POLARstar OPTIMA plate reader (BMG Labtechnologies, Germany).[10]

hHDAC8: Enzyme was obtained from cooperation partners (Romier).[11] Assay was performed as described before. 22.5 µL of enzyme solution in assay buffer (15 mM Tris, pH 7.5, 50 mM KH_2PO_4, 10 mM KCl, 3 mM $MgSO_4 \cdot 7H_2O$ and 0.2 mM TCEP), increasing inhibitor concentrations in DMSO (2.5 µL) and 5 µL of ZMTFAL substrate solution (150 µL) were incubated for 90 min at 37 °C in ½ AreaPlate-96 F microplates (PerkinElmer). 30 µL of stop solution (see HDAC1/6) were added. After incubation (30 min at 37 °C) fluorescence signal was determined as mentioned for HDAC1/6.[12]

Cellular assays

Cell cultivation: HL60 cells were maintained in RPMI 1640 medium (PanBiotech) supplemented with 10% fetal calf serum (FCS, PanBiotech), 2 mM glutamine and antibiotics (Penicillin, Streptomycin). Hela cells were cultivated in Dulbecco's modified Eagle's medium supplemented with 10% FCS, 2 mM glutamine and antibiotics. Cells were passaged every 2 days.

MTS Assay: HL60 cells were plated in sterile 96-well plates (Perkin Elmer) at a density of 7500 cells per well in RPMI 1640 medium containing supplements. Cells were treated with 0.05% DMSO or various concentrations of inhibitor. Hela cells were also plated in sterile 96-well plates at a density of 2000 cells in DMEM medium with supplements and allowed

to adhere overnight. The next day, the cells were treated with 0.1% DMSO or various concentrations of inhibitor.

Cell proliferation was determined by using the Celltiter 96 AQ_{ueous} nonradioactive Proliferation Assay (Promega). After 72h of incubation time with inhibitor or DMSO, 20 µL of a mixture (20:1) consisting of MTS (3-(4,5-dimethylthiazol-2-yl)-5-(3-carboxymethoxyphenyl)-2-(4-sulfophenyl-2H-tetrazolium) and PMS (phenazine methosulfate) were added to each well. Absorption was measured after another 2-4 h with a BMG LABTECH POLARstar OPTIMA plate reader (BMG Labtechnologies, Germany). Experiments were performed in triplicates and GI_{50} values were calculated using the software Origin 2019. GI_{50} was defined as the concentration that led to 50% viable cells.

Cellular trypsin-based activity assay:[13] 1 x 10^4 Hela cells (50 µL) were seeded in sterile dark 96-well microtiter plates (Perkin Elmer) and incubated overnight at 37 °C and 5% CO_2 atmosphere. The next day, the medium was aspirated and the cells were incubated with different inhibitor concentrations or DMSO, diluted in 50 µL medium without supplements and phenol red. After 2 h incubation time, 50 µL of the HDAC substrate MAL (Boc-Lys(Ac)-AMC) were added to a final concentration of 150 µM and the plate was incubated for 2 h at 37°C and 5% CO_2. The reaction was stopped by adding 50 µL of stop solution (50 mM Tris, 137 mM NaCl, 1 mM $MgCl_2$, 2.7 mM KCl, 1 µM TSA, 0.1% Igepal, 1 mg/ml Trypsin). After another 20 min of incubation, the fluorescence signal (λ_{ex} = 390 nm, λ_{em} = 460 nm) was measured on a BMG LABTECH POLARstar OPTIMA plate reader (BMG Labtechnologies, Germany).

Western Blot: 2.5 x 10^5 HL60 cells were seeded in sterile 12-well plates and incubated with different concentrations of compound or DMSO for 4 h. Cells were washed with cold PBS, lysed with 90 µL SDS sample buffer (Cell Signaling, 62.5 mM Tris-HCl (pH 6.8 at 25°C), 2% w/v SDS, 10% v/v glycerol, 50 mM dithiothreitol, 0.01% bromophenol blue), shortly sonicated and heated to 95°C for 5 min. Cell extracts were used directly for SDS-PAGE or kept frozen at -20 °C until usage.

For the SDS-PAGE, 7 µL cell lysate were loaded on a 12.5% gel and the gel was run at 150V for approximately 1 h, followed by the transfer to a nitrocellulose membrane. The membrane was blocked with 5% milk in TBS-T 0.1% for 1 h at room temperature and washed (3 x 5 min) with TBS-T before adding the antibodies. Western blot analysis was performed with the following primary antibodies: anti-acetylated α-tubulin (Sigma-Aldrich, 1:1000), anti-acetyl-histone 3 (Millipore, 1:2000) and anti-GAPDH (Sigma-Aldrich, 1:5000) and secondary antibodies: anti-mouse-IgG-HRP (Sigma-Aldrich, 1:10000) and anti-rabbit IgG-HRP (Sigma-

Aldrich, 1:5000). The antibodies were all diluted in 3% milk in TBS-T 0.1%. Detection was performed via enhanced chemiluminescence (ECL Prime) using a FUSION-SL (PEQLAB) and the FUSION-CAPT software.

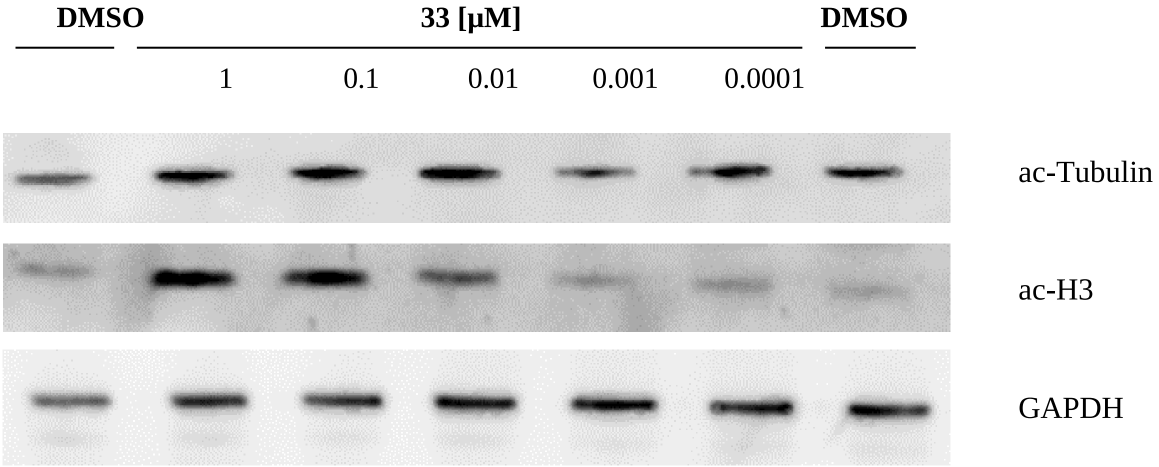

Figure 4: Western blot of acetyl-α-tubulin, acetyl-H3 and GAPDH after treatment of HL60 cells with different concentrations of 33 or DMSO for 4 h. GAPDH was used as loading control, DMSO as negative control.

33 [µM]	ac-histone 3 [%]	ac-tubulin [%]
1	5.0	1.4
0.1	4.4	1.3
0.01	1.8	1.1
0.001	0.9	0.5
0.0001	1.1	1.1

Spectral Data

(9*H*-Fluoren-9-yl)methyl (*R*)-hept-1-en-3-ylcarbamate (17)

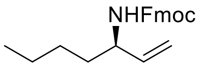

17

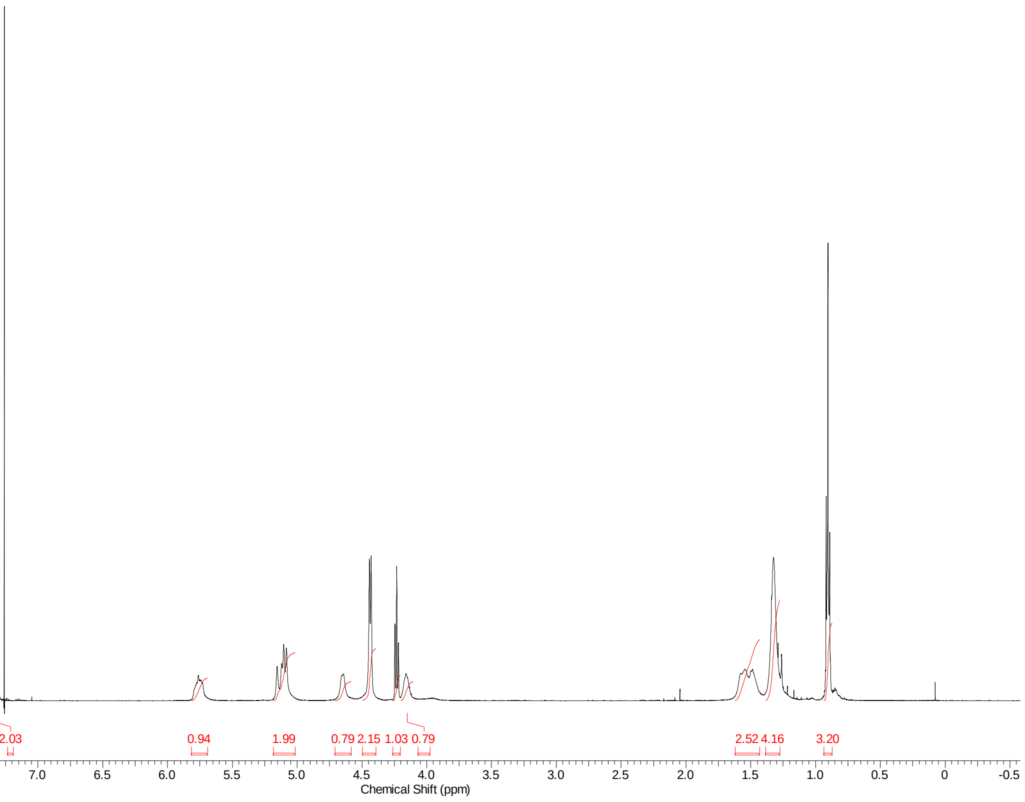

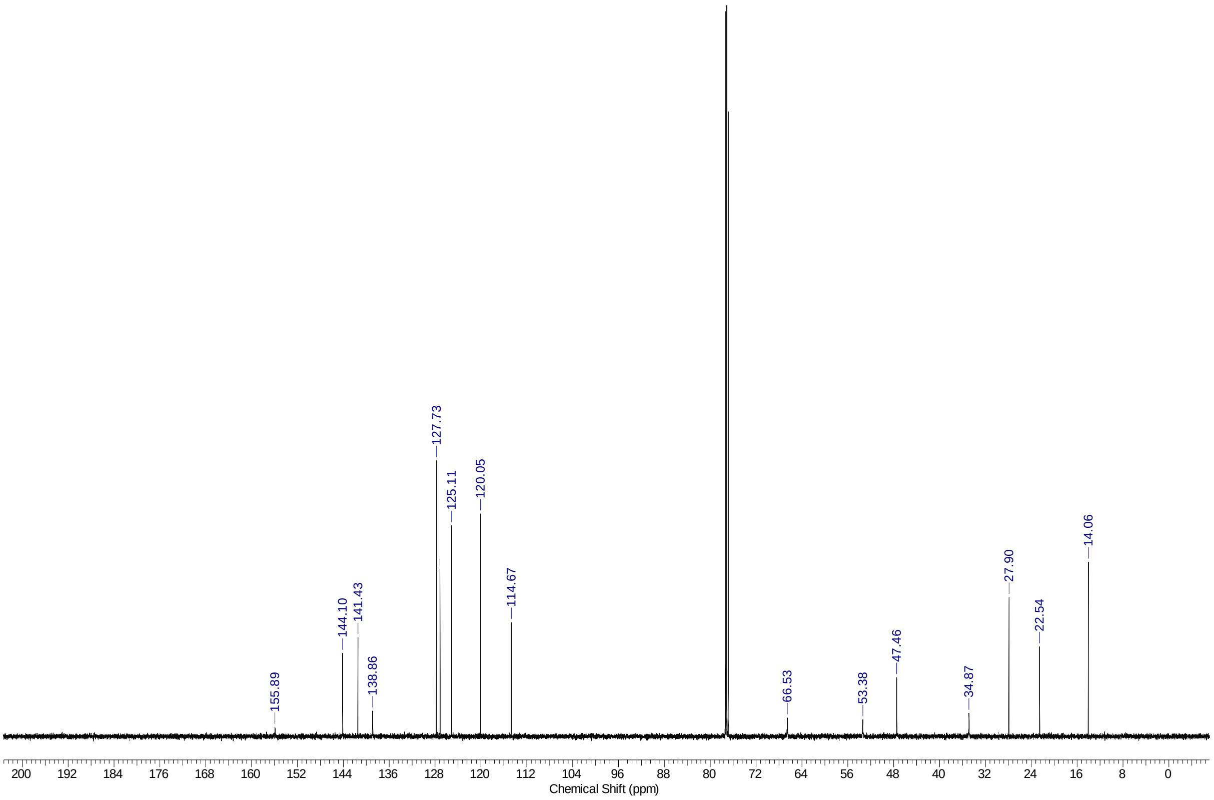

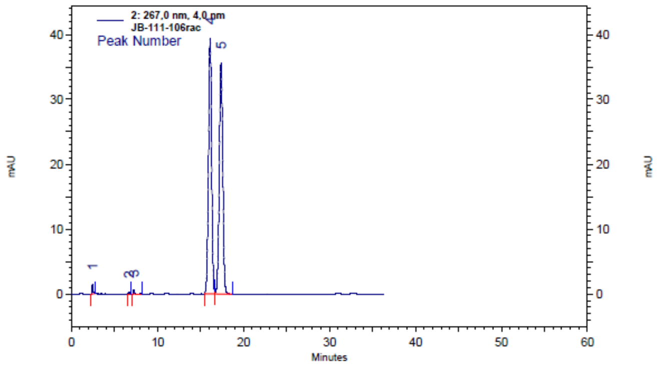

2: 267,0 nm, 4,0 nm Results

Peak Number	Retention Time	Area Percent	Area
1	2,440	0,497	1352173
2	6,707	0,122	330737
3	7,253	0,375	1018667
4	16,127	49,404	134367883
5	17,400	49,603	134908274
Totals		100,000	271977734

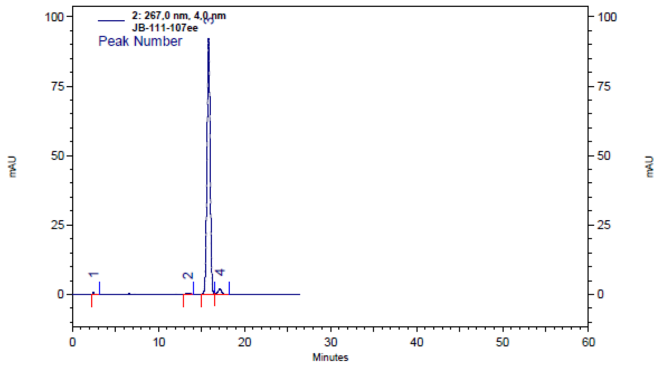

2: 267,0 nm, 4,0 nm Results

Peak Number	Retention Time	Area Percent	Area
1	2,440	0,300	971966
2	13,427	0,535	1735250
3	15,827	96,846	313967029
4	17,140	2,319	7518919
Totals		100,000	324193164

Benzyl (*R*)-hept-1-en-3-ylcarbamate (18)

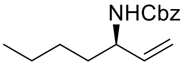

18

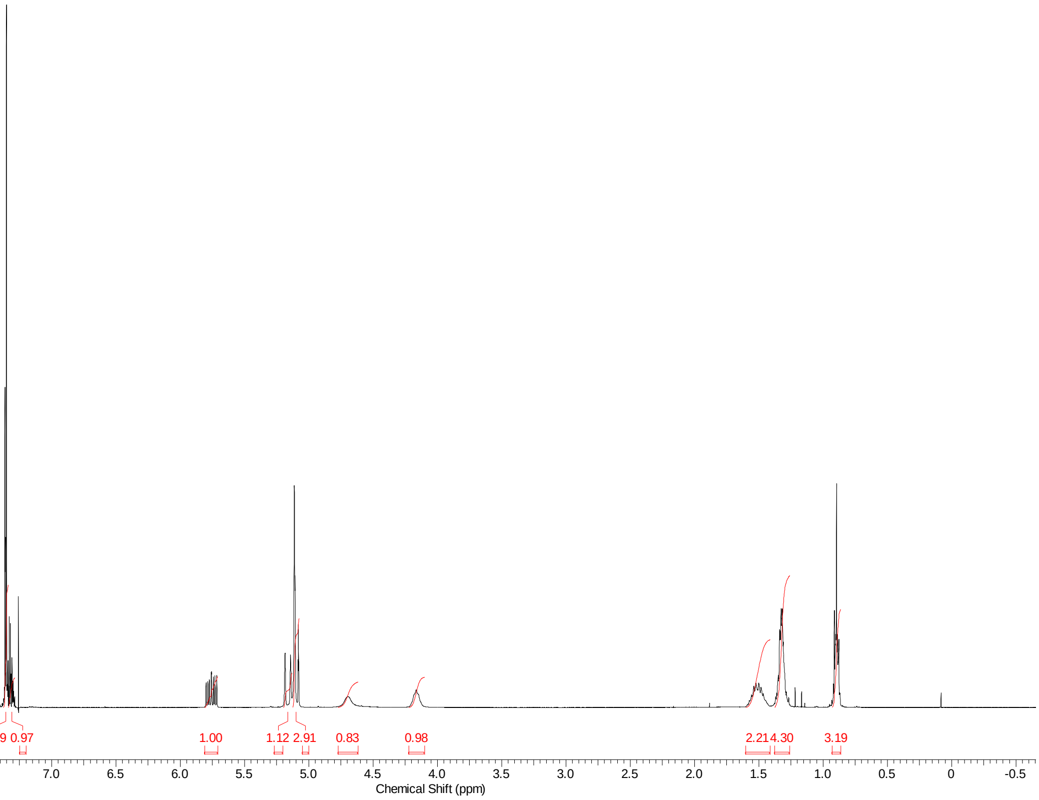

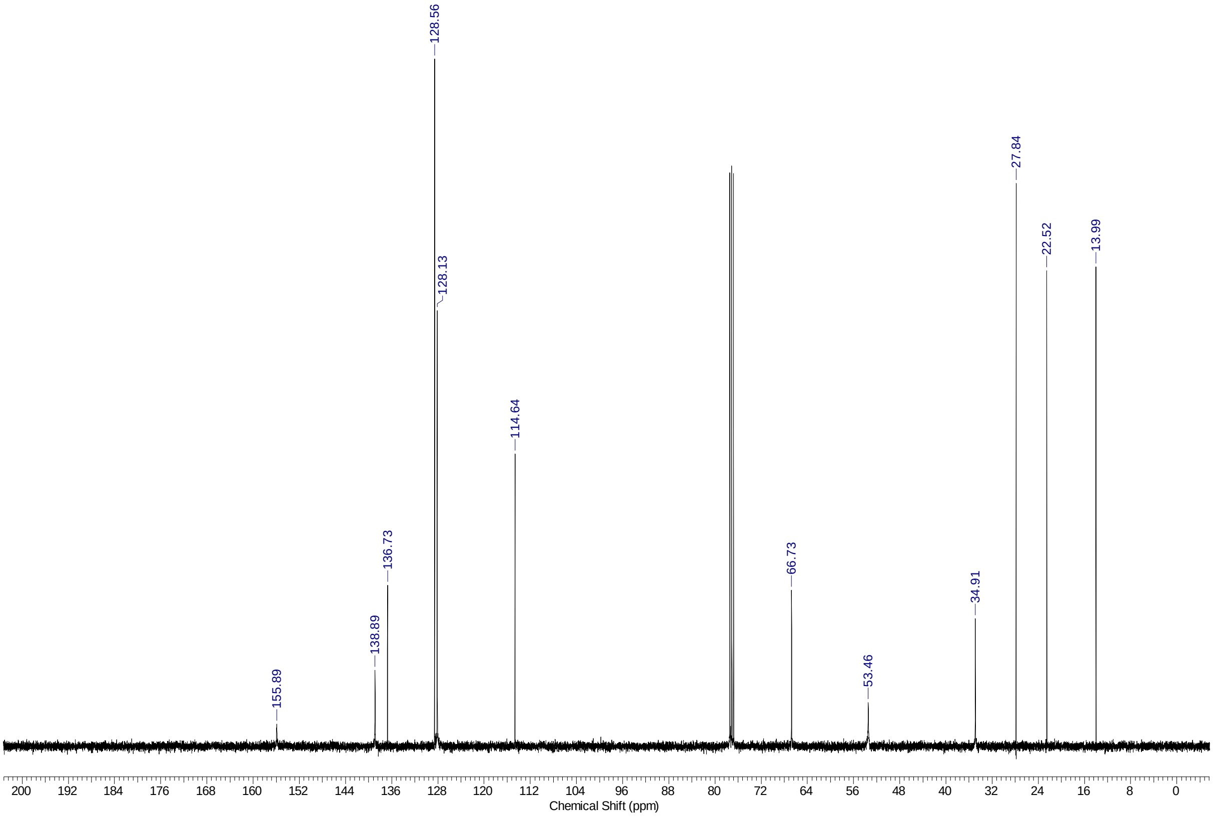

(9H-Fluoren-9-yl)methyl-((3R)-1,2-dihydroxyheptan-3-yl)carbamate (41)

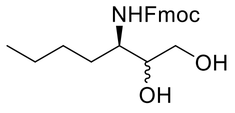

41

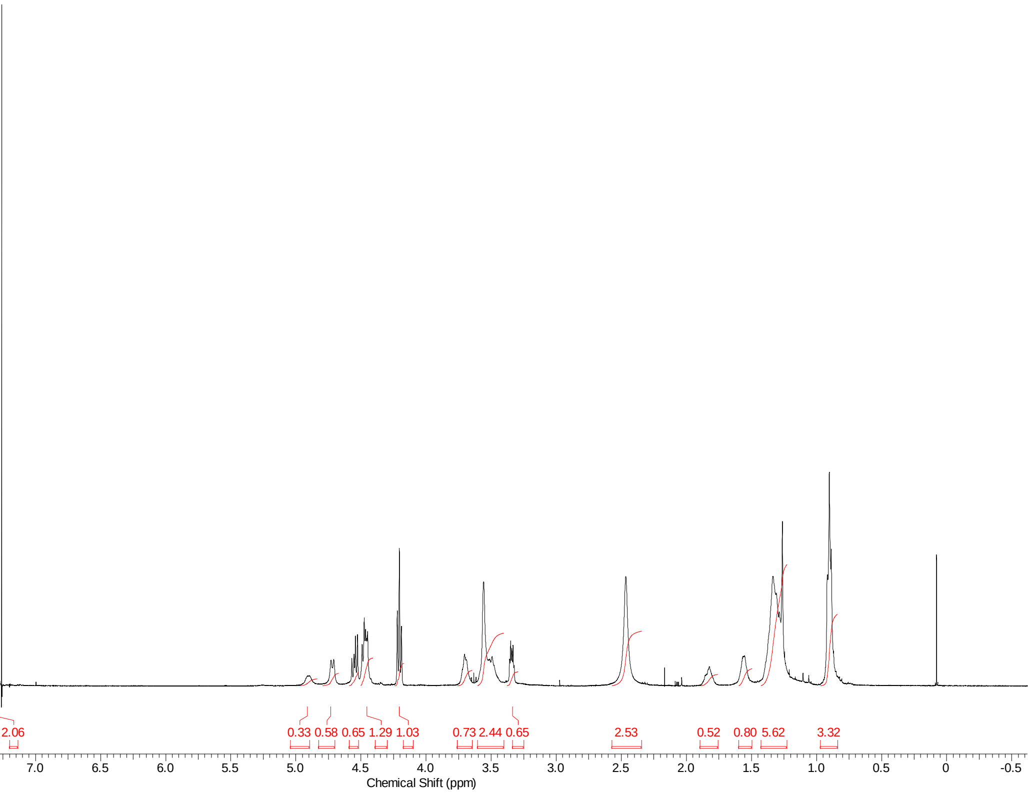

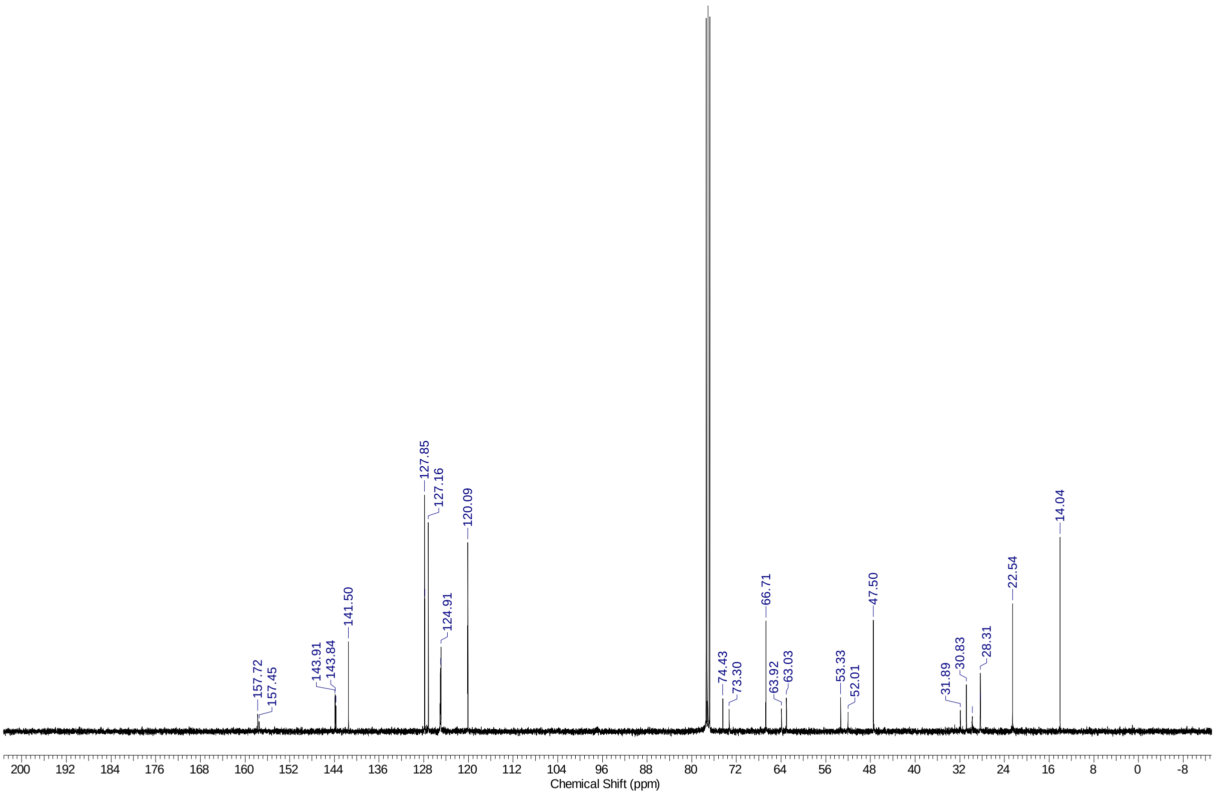

(*R*)-((9*H*-Fluoren-9-yl)methoxy)carbonyl-D-norleucine (19)

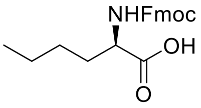

19

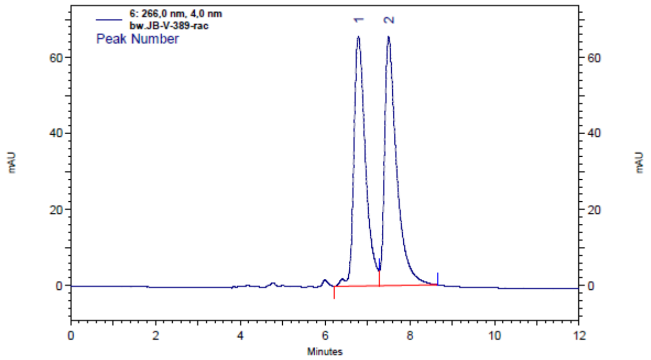

6: 266,0 nm, 4,0 nm Results

Peak Number	Retention Time	Area Percent	Area
1	6,790	49,053	168562796
2	7,503	50,947	175074660
Totals		100,000	343637456

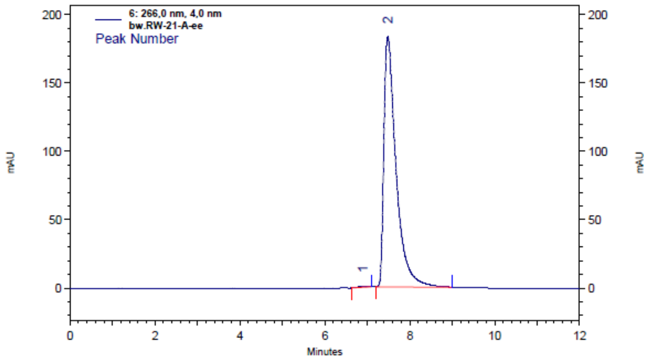

6: 266,0 nm, 4,0 nm Results

Peak Number	Retention Time	Area Percent	Area
1	6,893	0,271	1327774
2	7,483	99,729	488912079
Totals		100,000	490239853

Benzyl-((3*R*)-1,2-dihydroxyheptan-3-yl)carbamate (42)

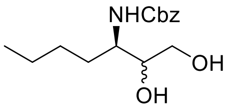

42

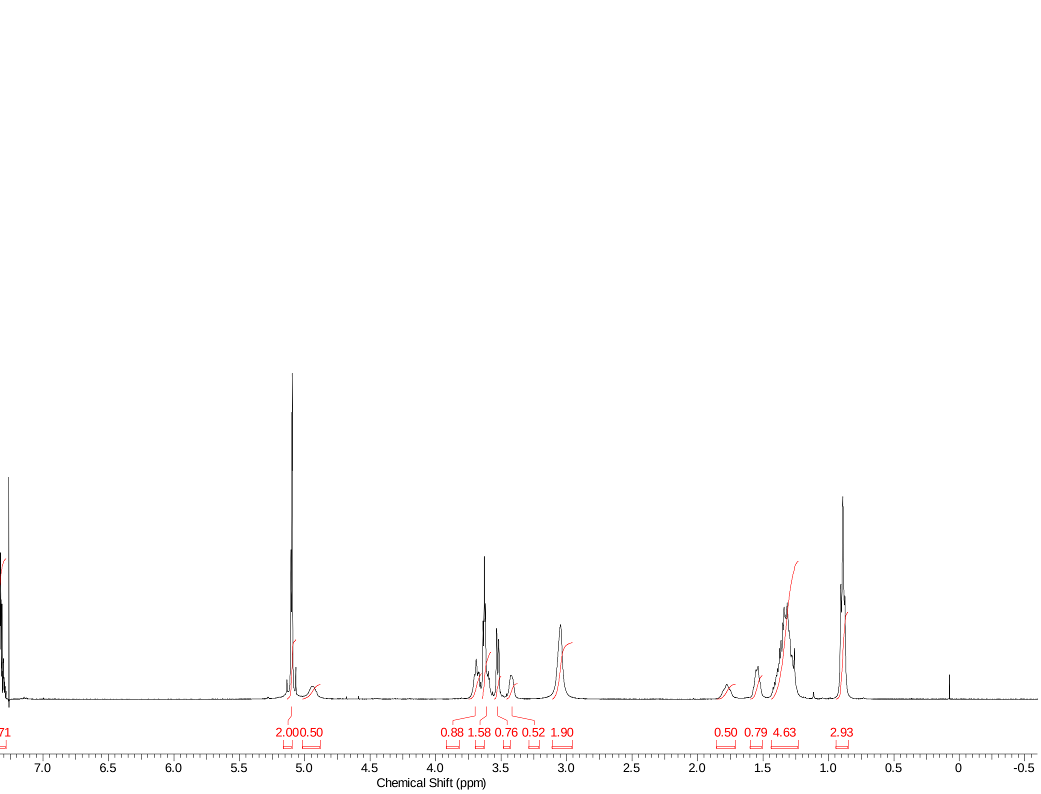

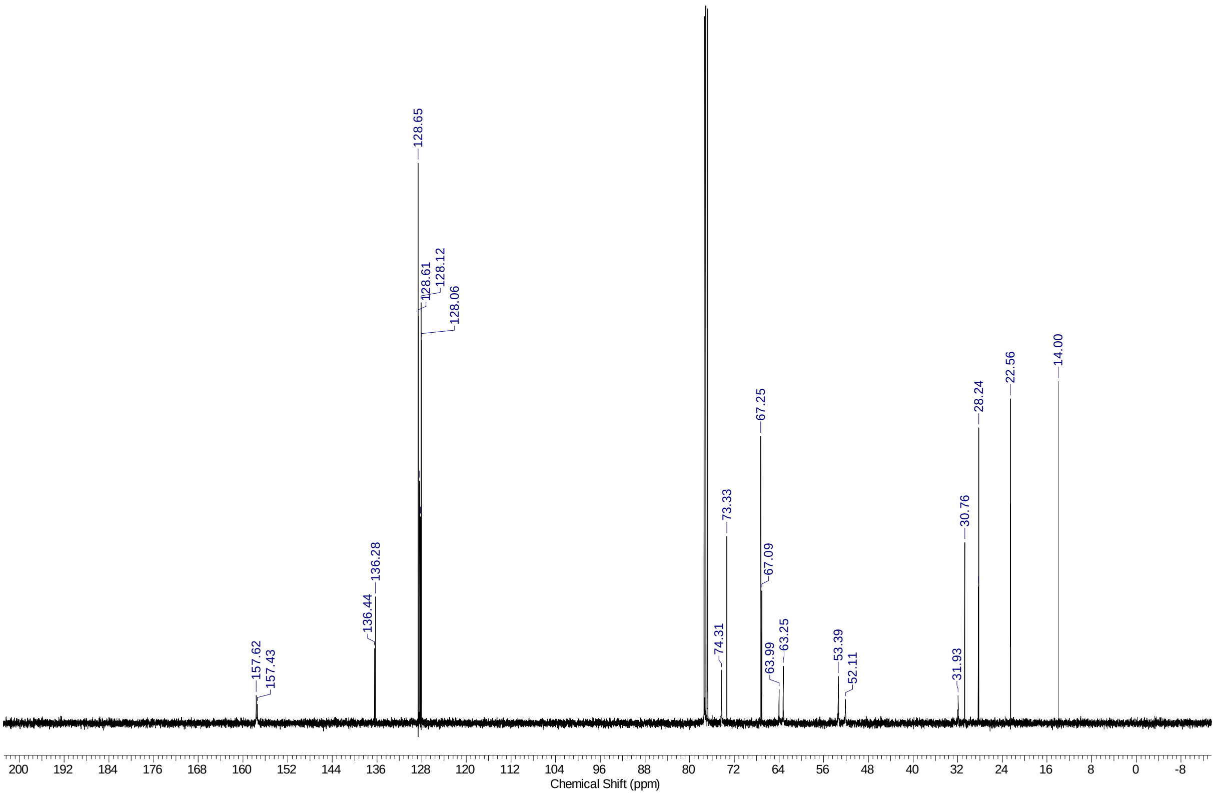

(*R*)-(((Benzyloxy)carbonyl)amino)-D-norleucine (20)

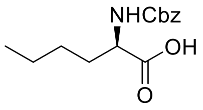

20

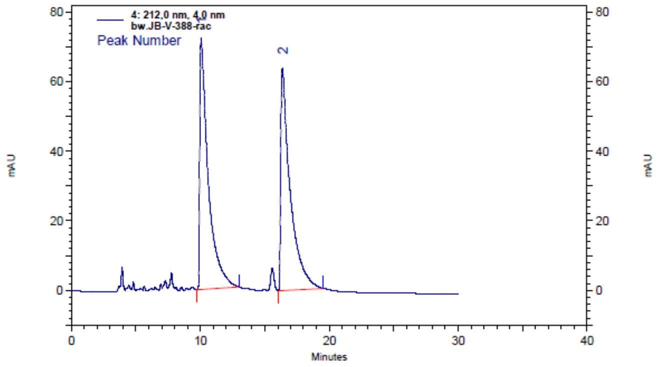

4: 212,0 nm, 4,0 nm Results

Peak Number	Retention Time	Area Percent	Area
1	10,060	50,004	441094475
2	16,373	49,996	441016199
Totals		100,000	882110674

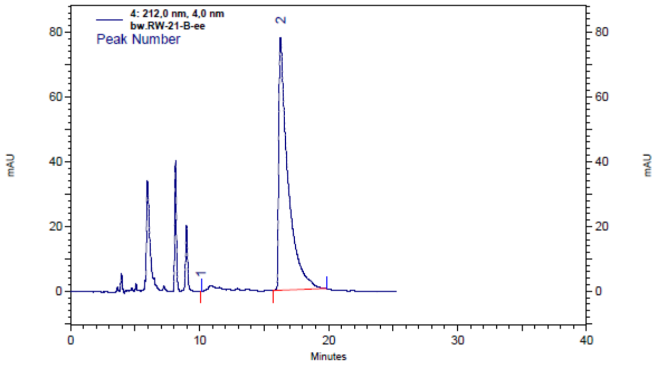

4: 212,0 nm, 4,0 nm Results

Peak Number	Retention Time	Area Percent	Area
1	10,093	0,002	9633
2	16,267	99,998	523554117
Totals		100,000	523563750

tert-Butyl ((*R*)-2-((((9*H*-fluoren-9-yl)methoxy)carbonyl)amino)hexanoyl)-D-alaninate (22)

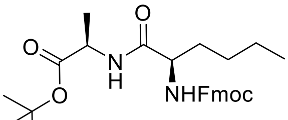

22

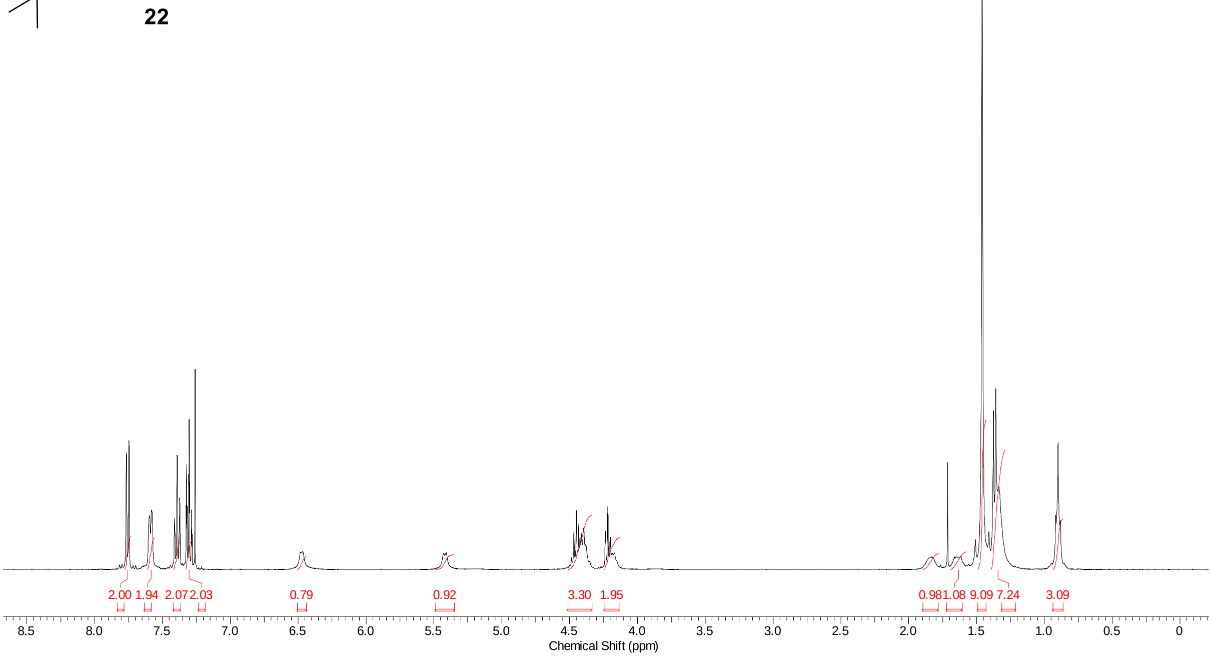

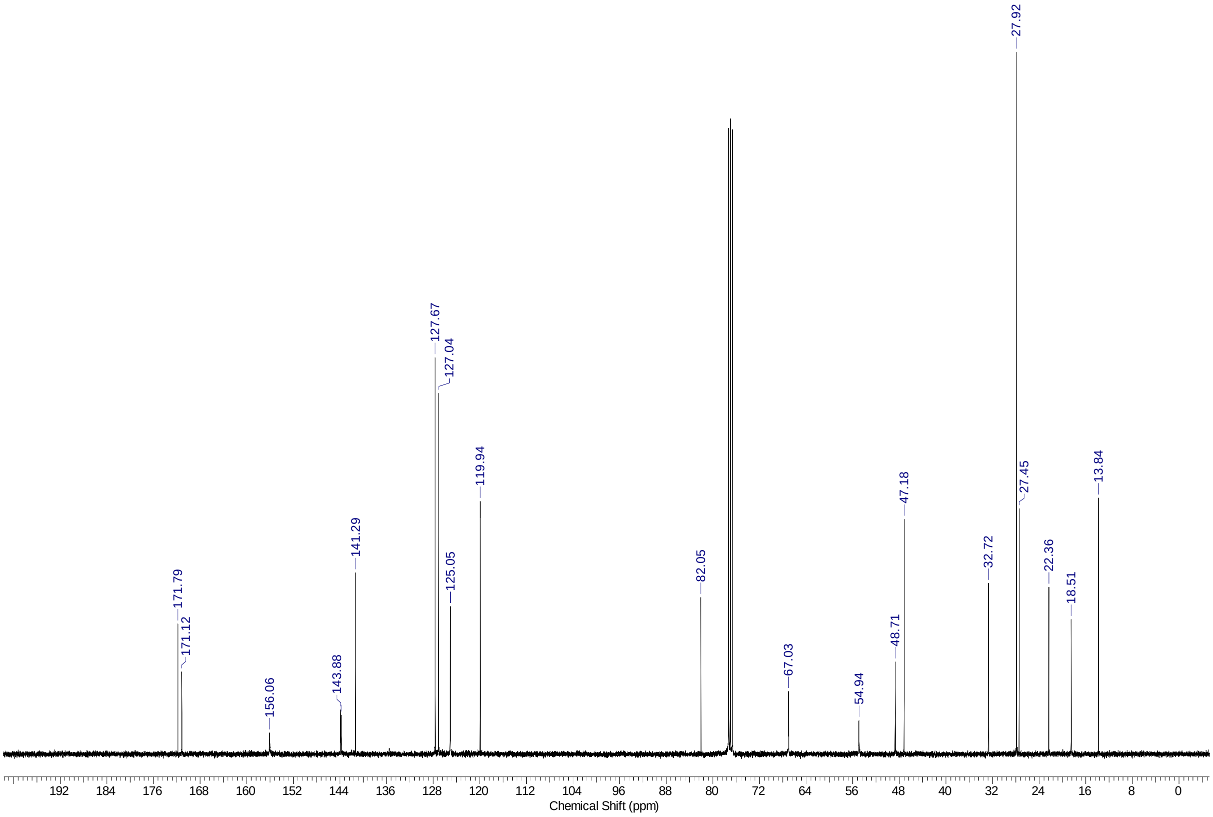

((*R*)-2-((((9*H*-fluoren-9-yl)methoxy)carbonyl)amino)hexanoyl)-D-alanine (12)

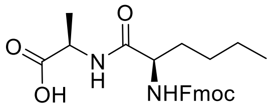

12

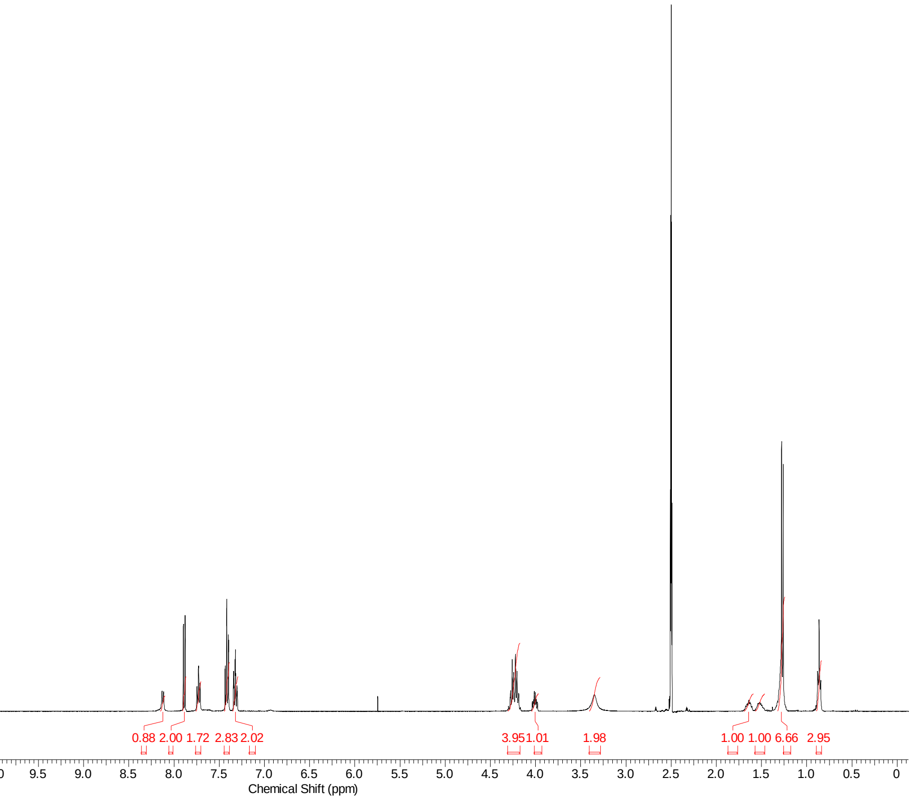

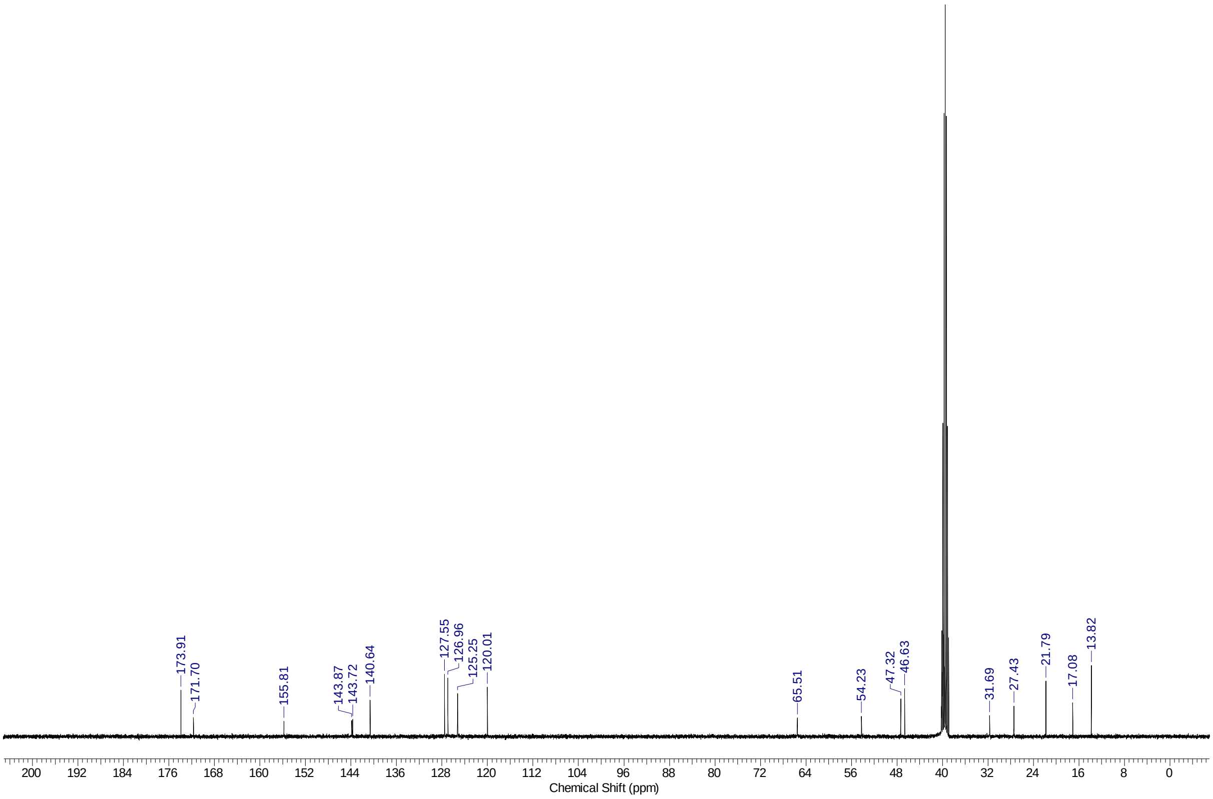

***tert*-Butyl (3*S*,4*R*,5*S*)-4-((((9*H*-fluoren-9-yl)methoxy)carbonyl)amino)-3-((*tert*-butyldimethylsilyl)oxy)-5-methylheptanoate (24)**

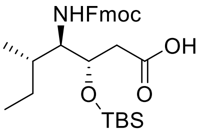

24

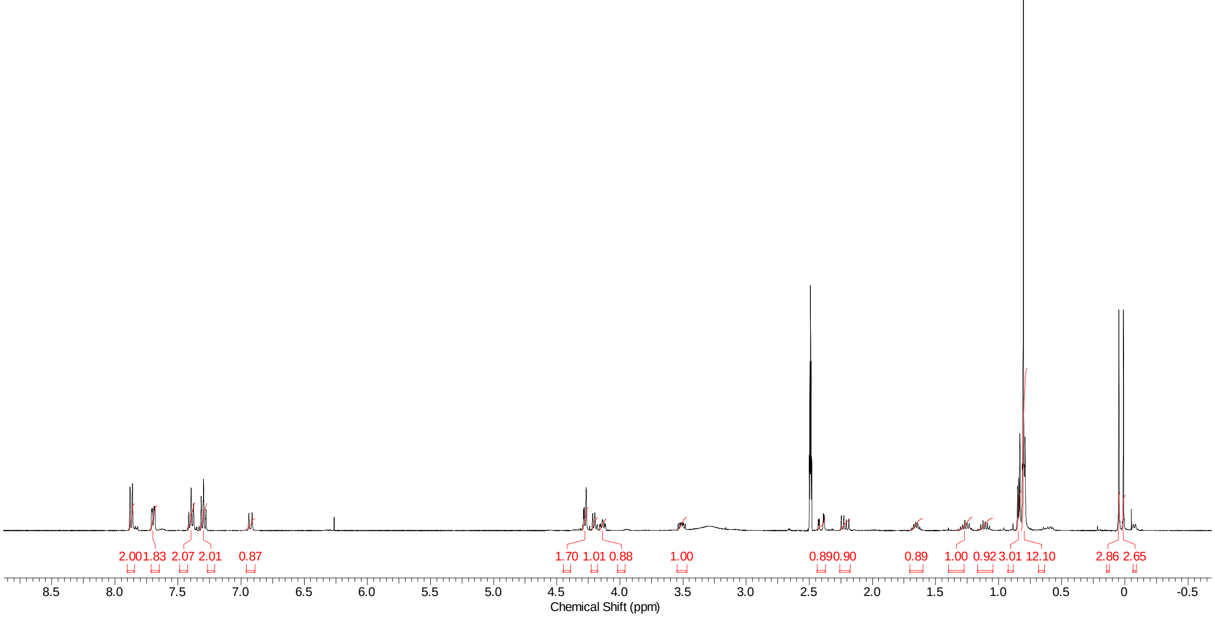

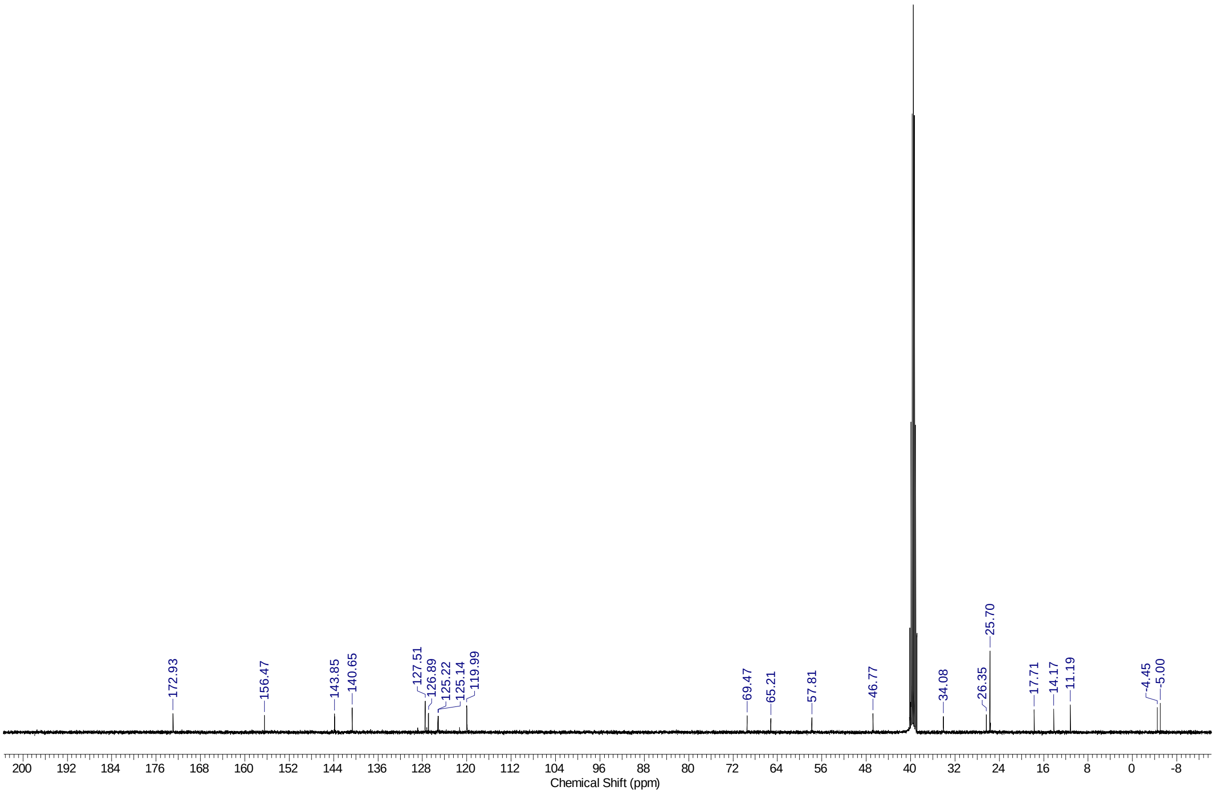

(3S,4R,5S)-((S)-5-(tert-Butyldimethylsilyloxy)pent-1-en-3-yl) 4-(((9H-fluoren-9-yl)methoxy)carbonylamino)-3-(tert-butyldimethylsilyloxy)-5-methylheptanoate (26)

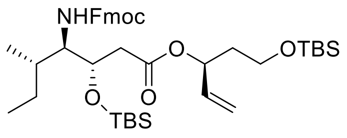

26

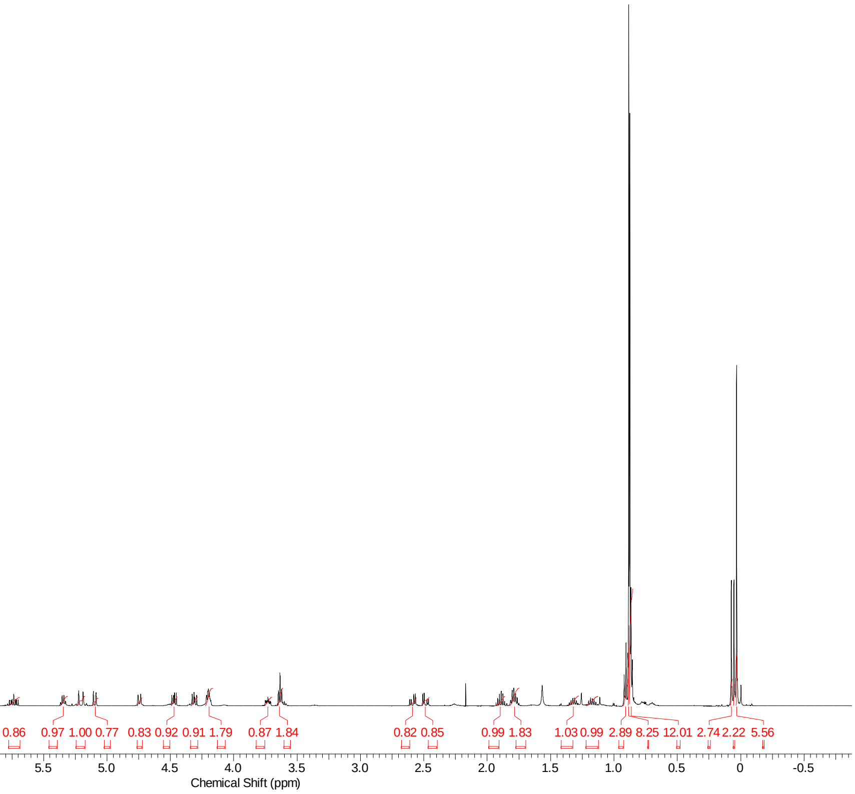

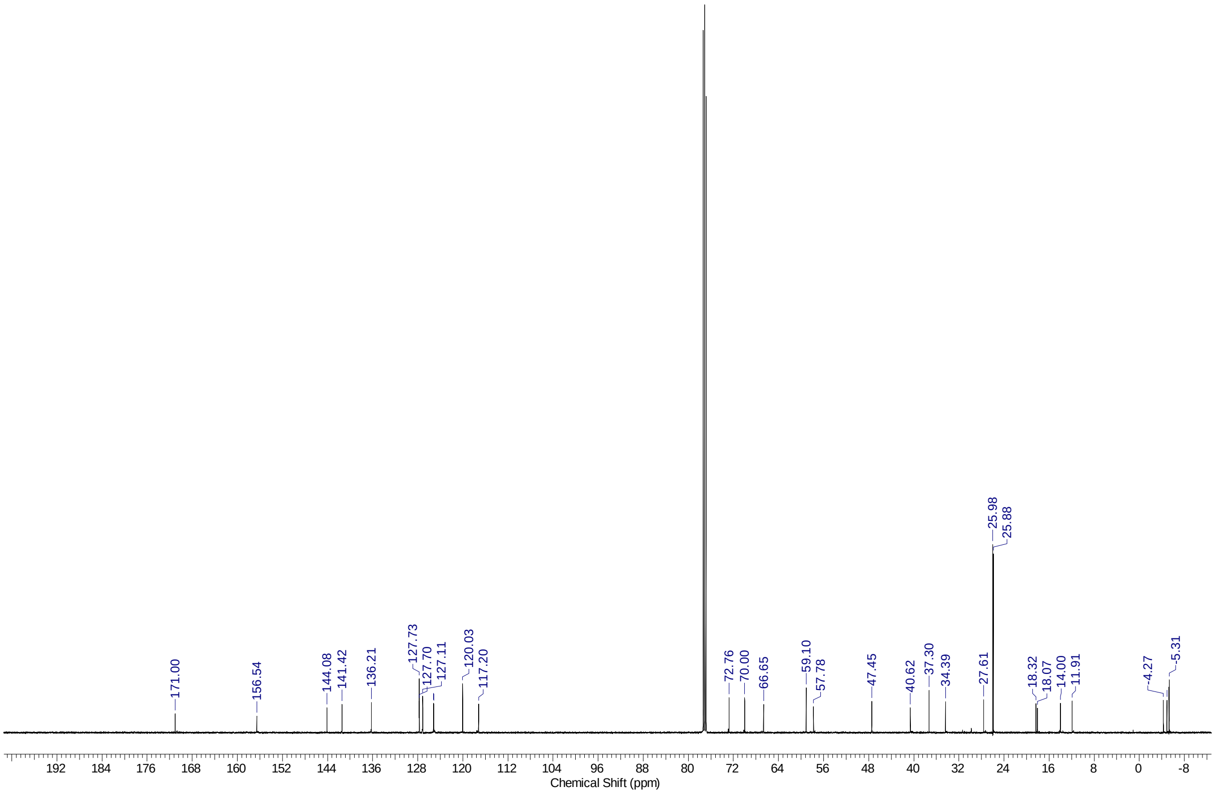

(*R*)-5-((*tert*-Butyldimethylsilyl)oxy)pent-1-en-3-yl (3*S*,4*R*,5*S*)-4-((((9*H*-fluoren-9-yl)methoxy)carbonyl)amino)-3-((*tert*-butyldimethylsilyl)oxy)-5-methylheptanoate (26)

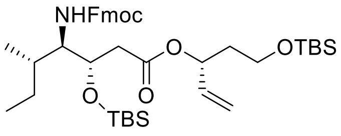

26

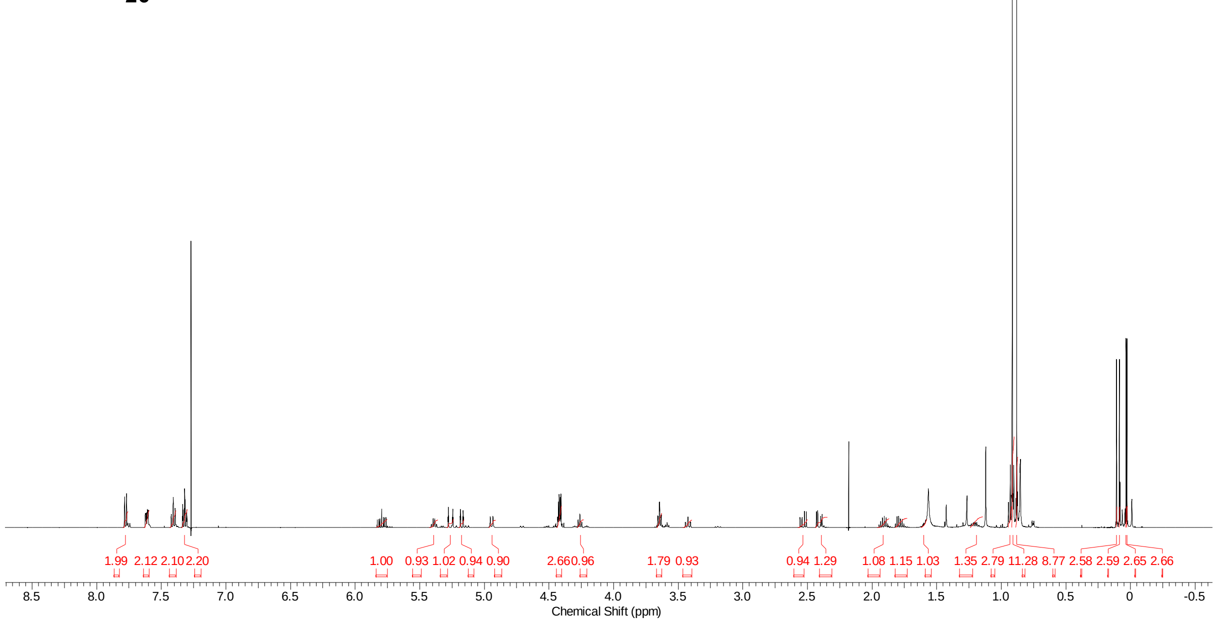

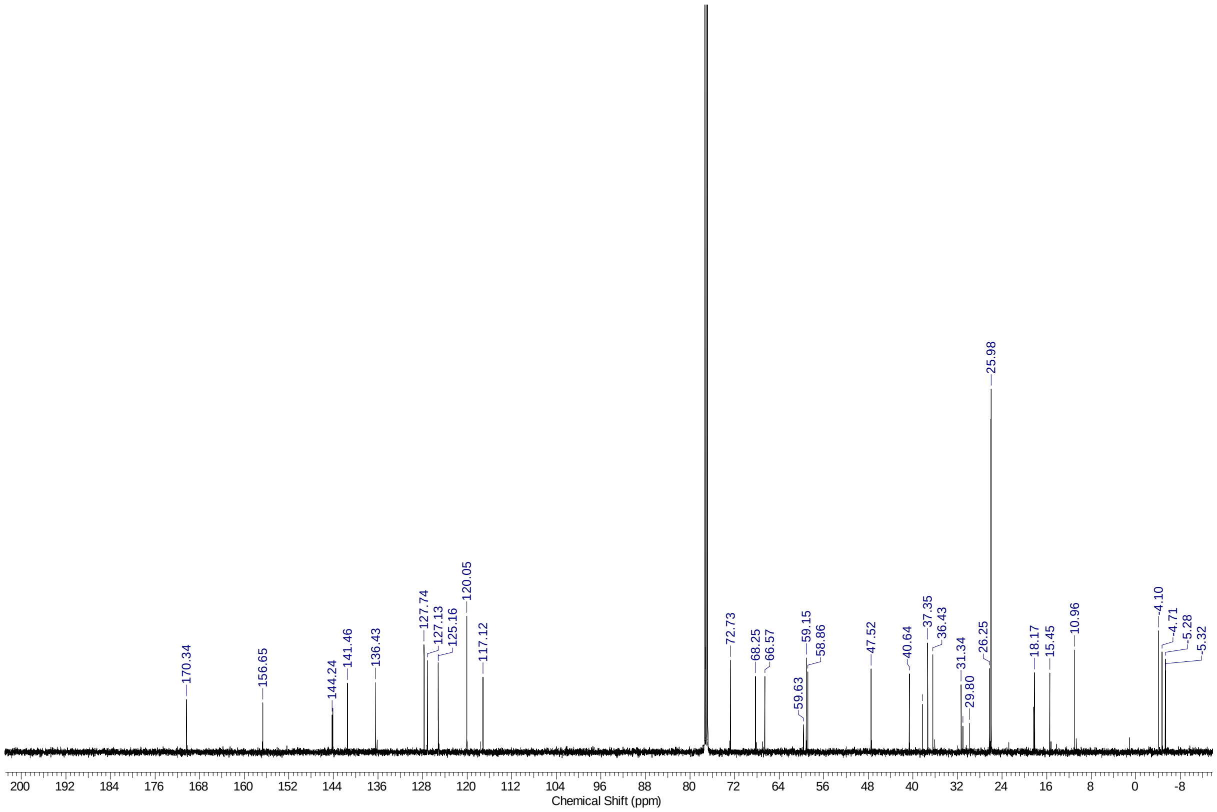

(*S*)-5-((*tert*-Butyldimethylsilyl)oxy)pent-1-en-3-ol (45)

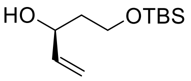

45

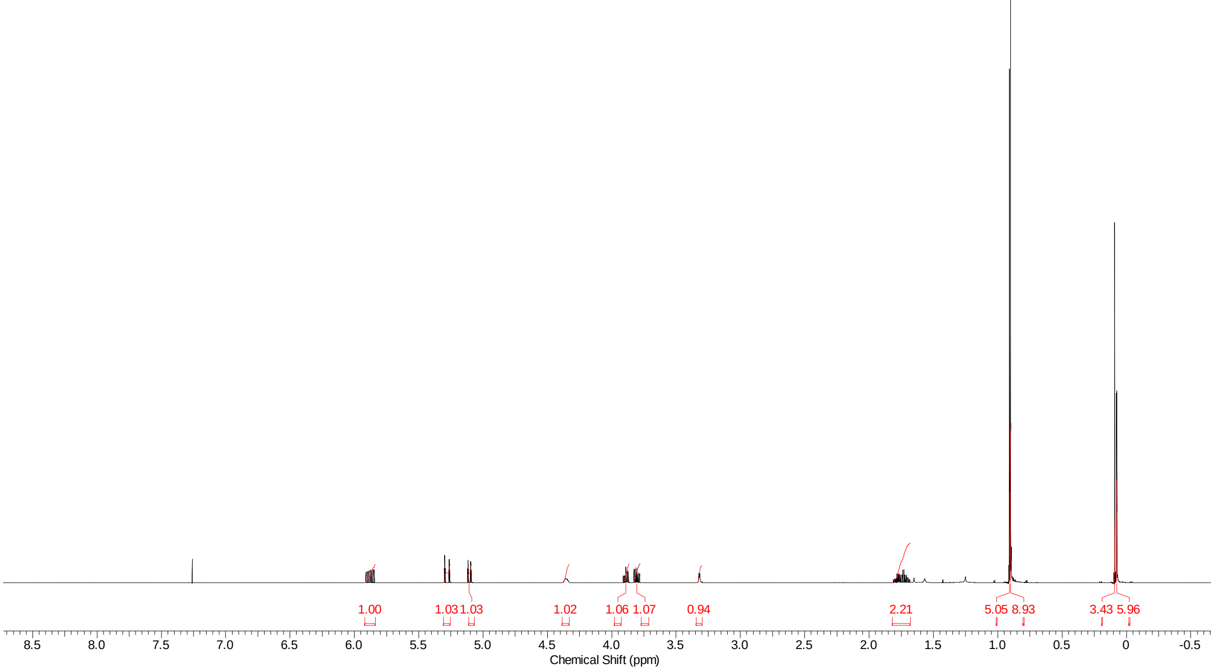

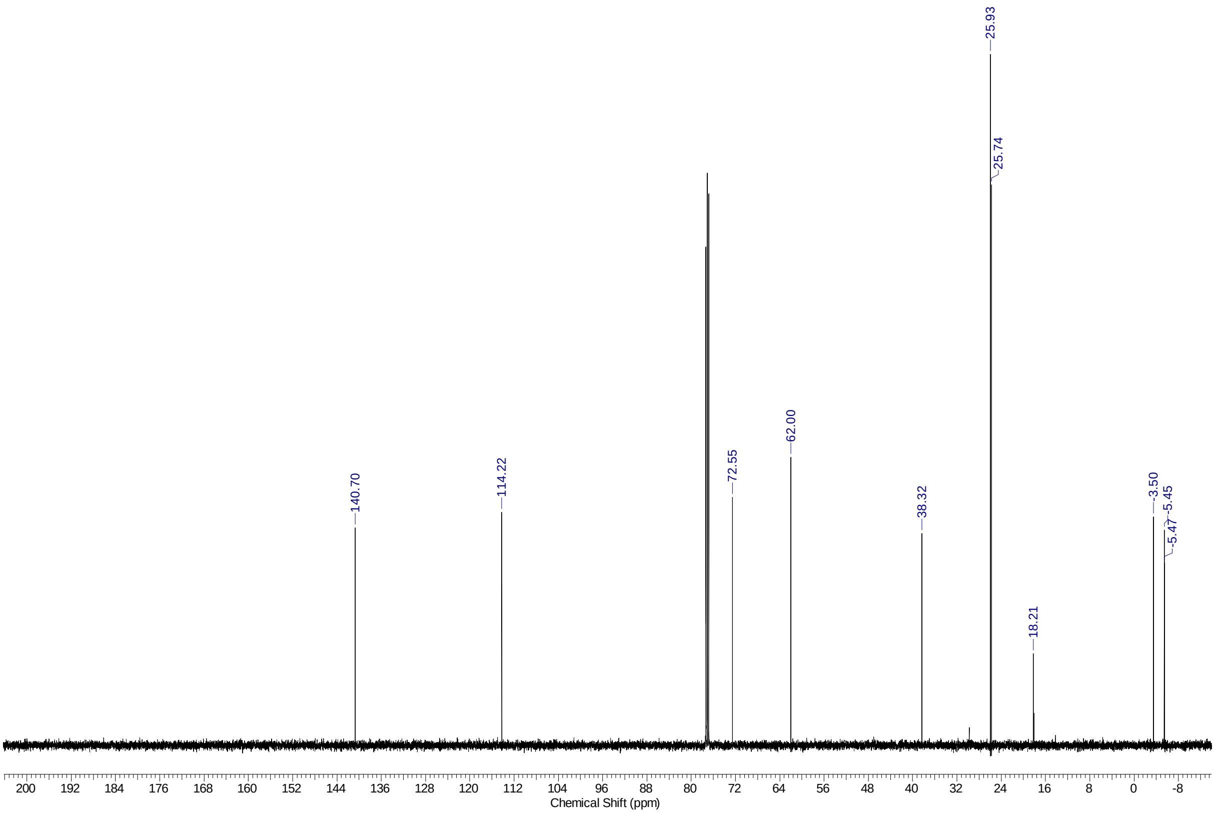

(*S*)-5-((*tert*-Butyldimethylsilyl)oxy)pent-1-en-3-yl (*R*)-3,3,3-trifluoro-2-methoxy-2-phenylpropanoate (*R*)-47

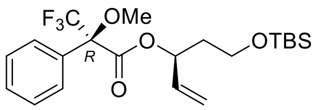

(*R*)-47

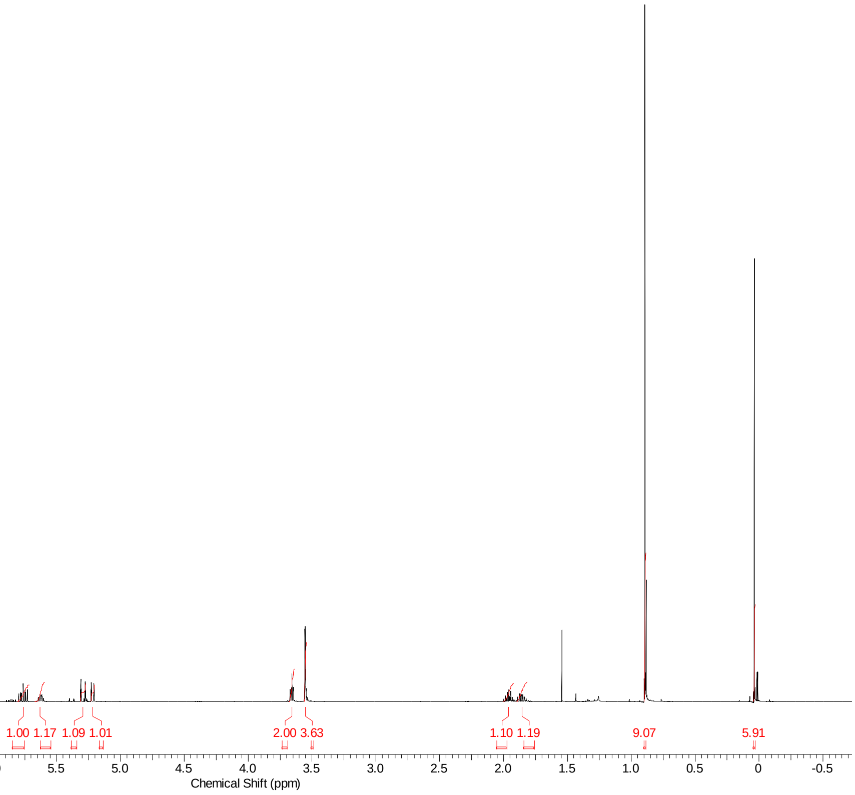

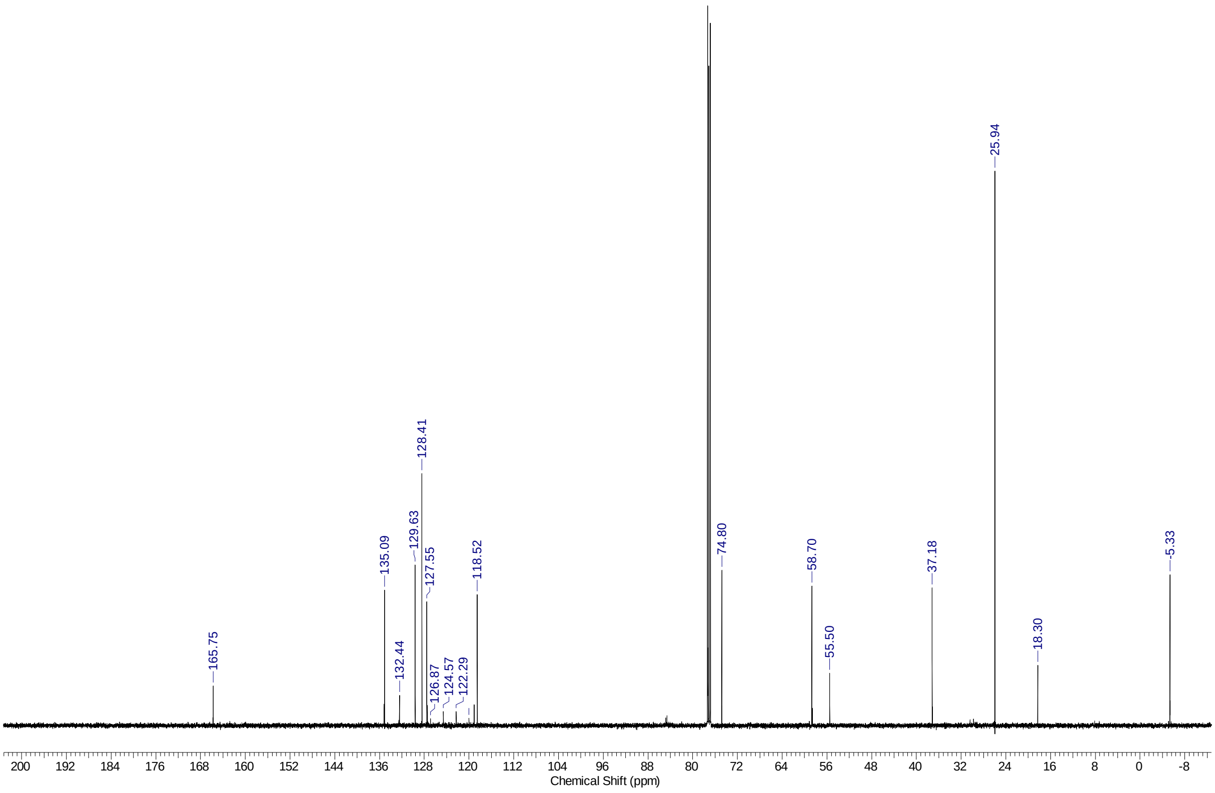

(*S*)-5-((*tert*-Butyldimethylsilyl)oxy)pent-1-en-3-yl (*S*)-3,3,3-trifluoro-2-methoxy-2-phenylpropanoate (*S*)-47

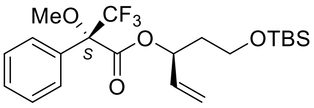

(*S*)-47

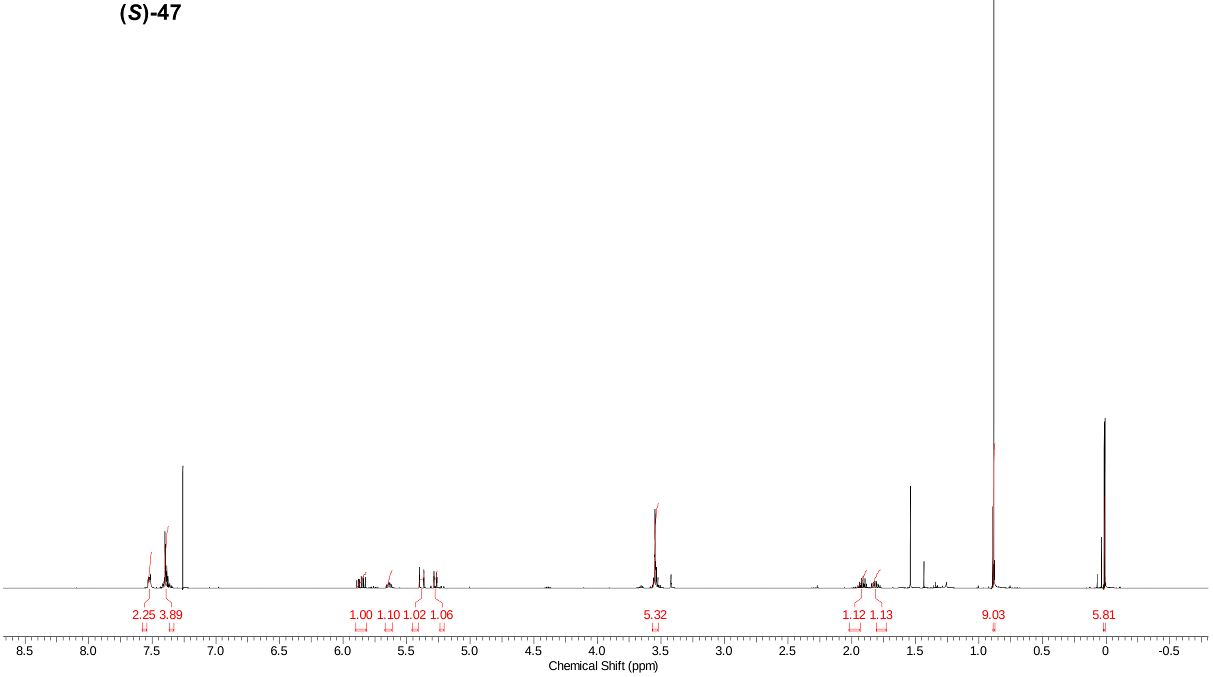

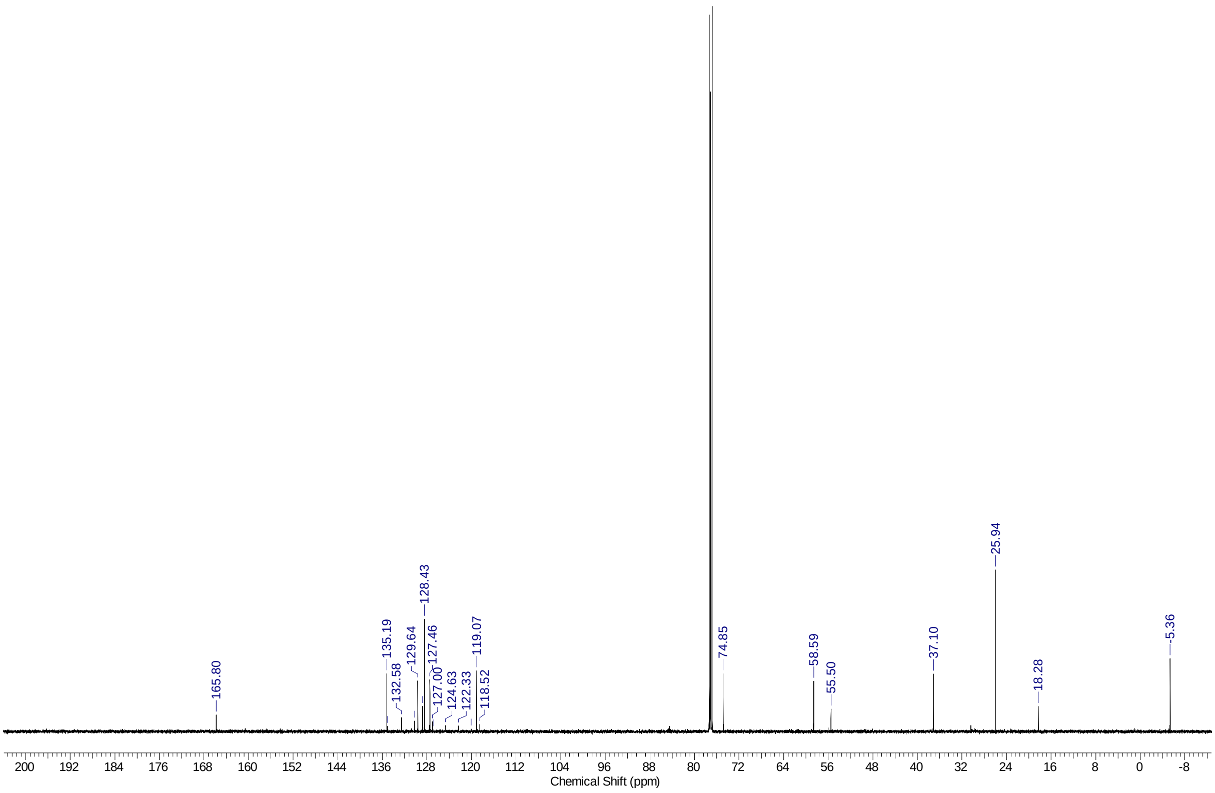

(*S*)-5-((*tert*-Butyldimethylsilyl)oxy)pent-1-en-3-yl (3*S*,4*R*,5*S*)-4-amino-3-((*tert*-butyldimethylsilyl)oxy)-5-methylheptanoate (13)

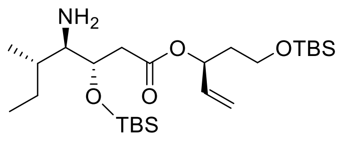

13

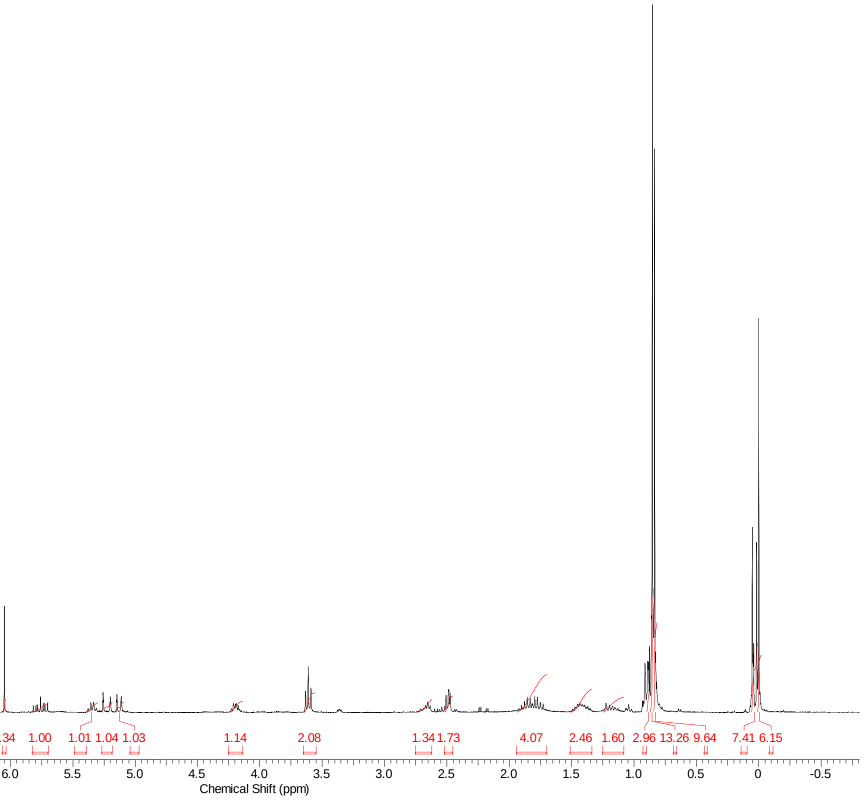

(2*S*,6*R*,9*R*,12*R*,13*S*)-12-((*S*)-*sec*-Butyl)-6-butyl-13-((*tert*-butyldimethylsilyl)oxy)-9-methyl-2-vinyl-1-oxa-5,8,11-triazacyclopentadecane-4,7,10,15-tetraone (27)

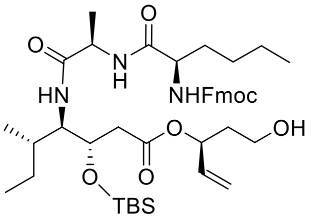

27

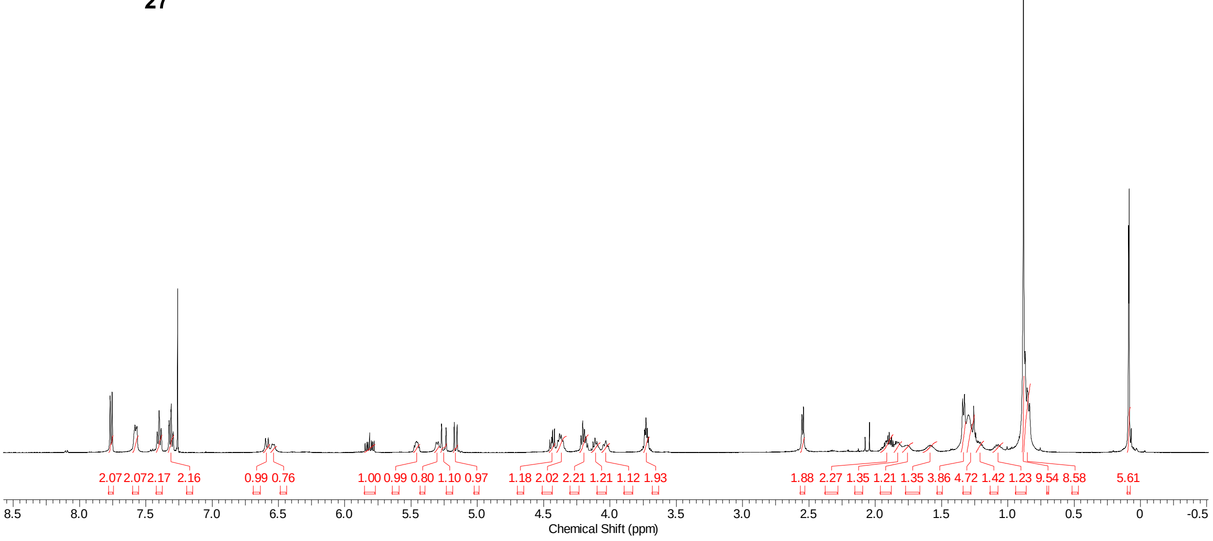

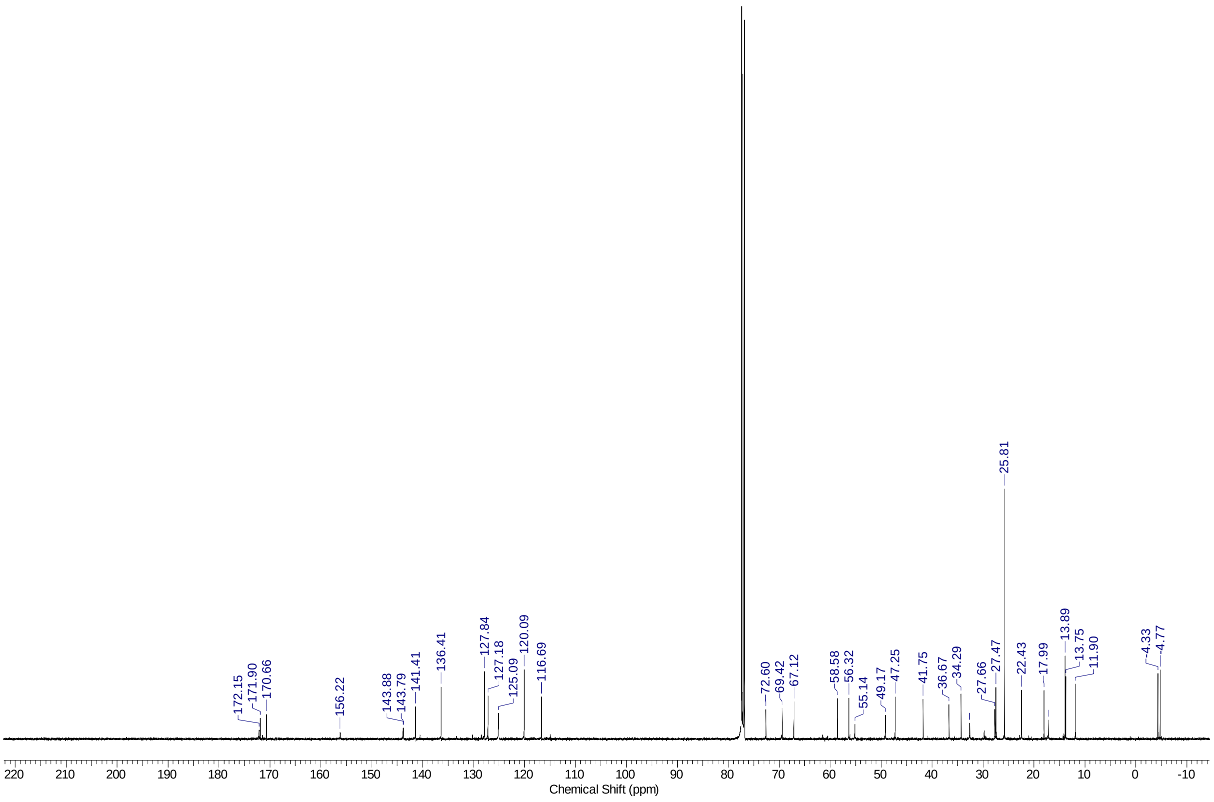

(5*R*,8*R*,11*R*,12*S*,16*S*)-11-((*S*)-*sec*-Butyl)-5-butyl-12-((*tert*-butyldimethylsilyl)oxy)-1-(9*H*-fluoren-9-yl)-8-methyl-3,6,9,14-tetraoxo-16-vinyl-2,15-dioxa-4,7,10-triaza-octadecan-18-oic acid (10)

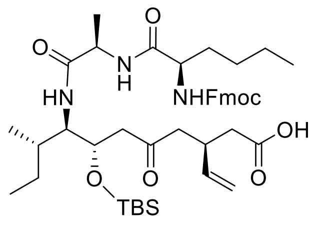

10

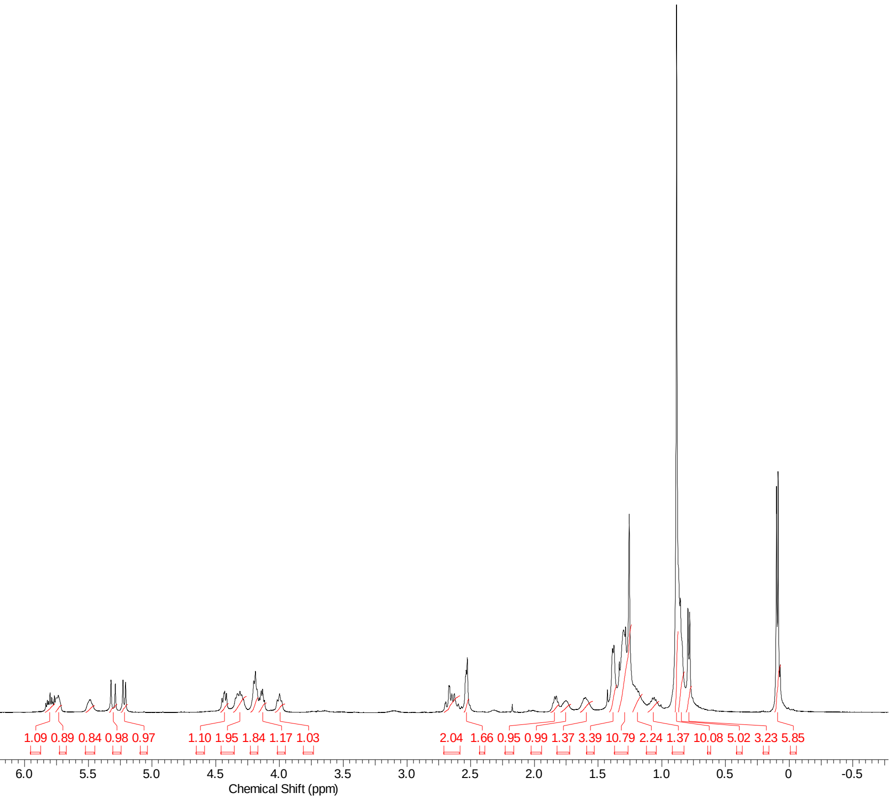

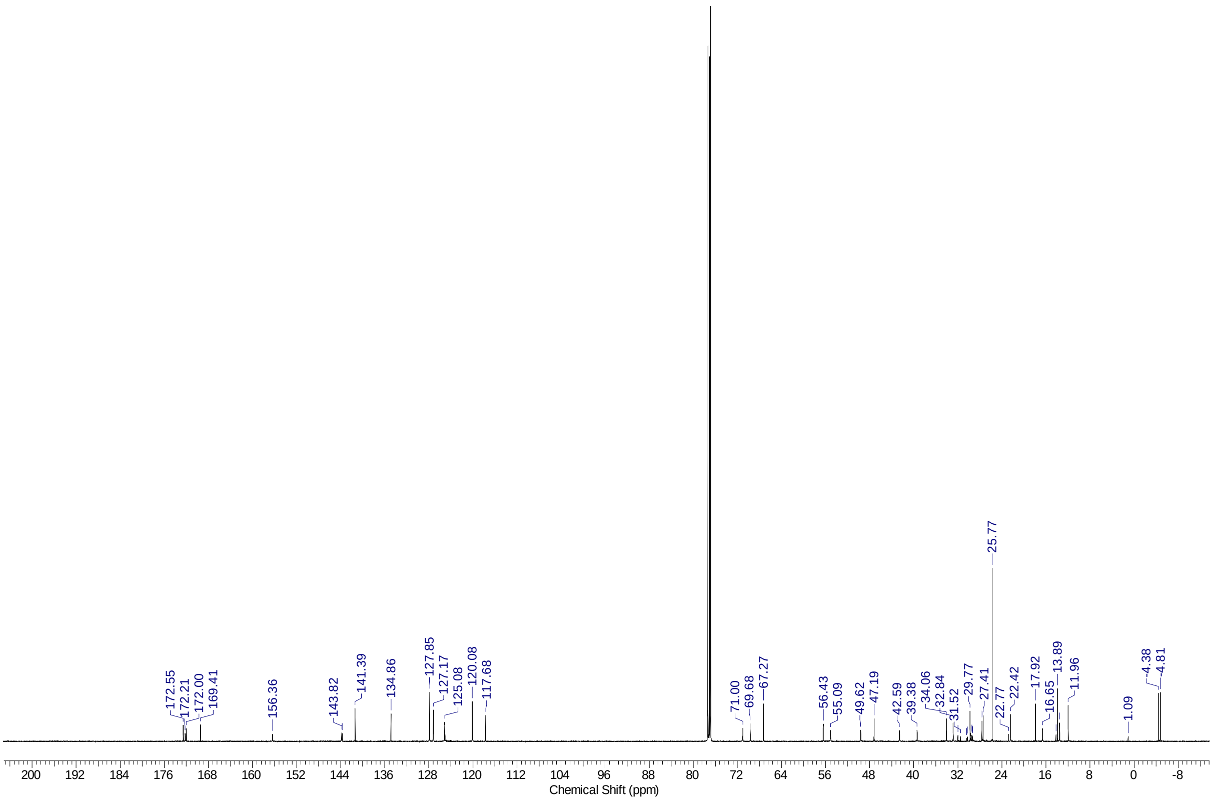

(*S*)-3-(((3*S*,4*R*,5*S*)-4-((*R*)-2-((*R*)-2-Aminohexanamido)propanamido)-3-((*tert*-butyldimethylsilyl)oxy)-5-methylheptanoyl)oxy)pent-4-enoic acid (48)

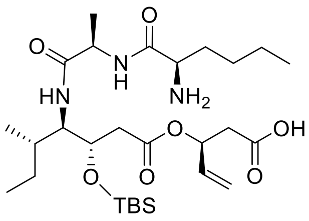

48

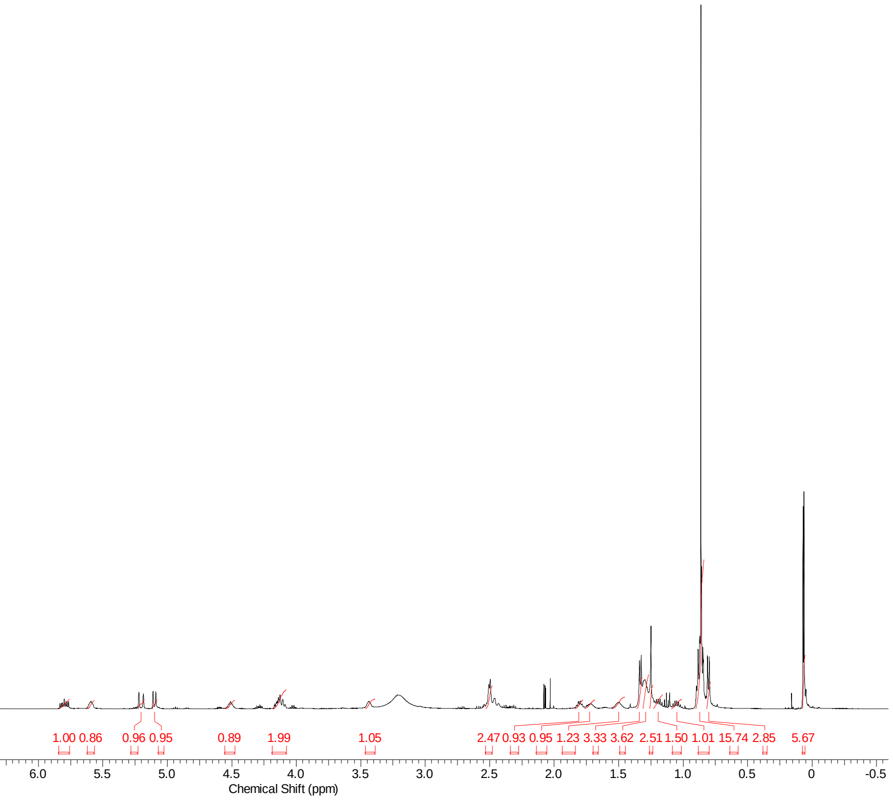

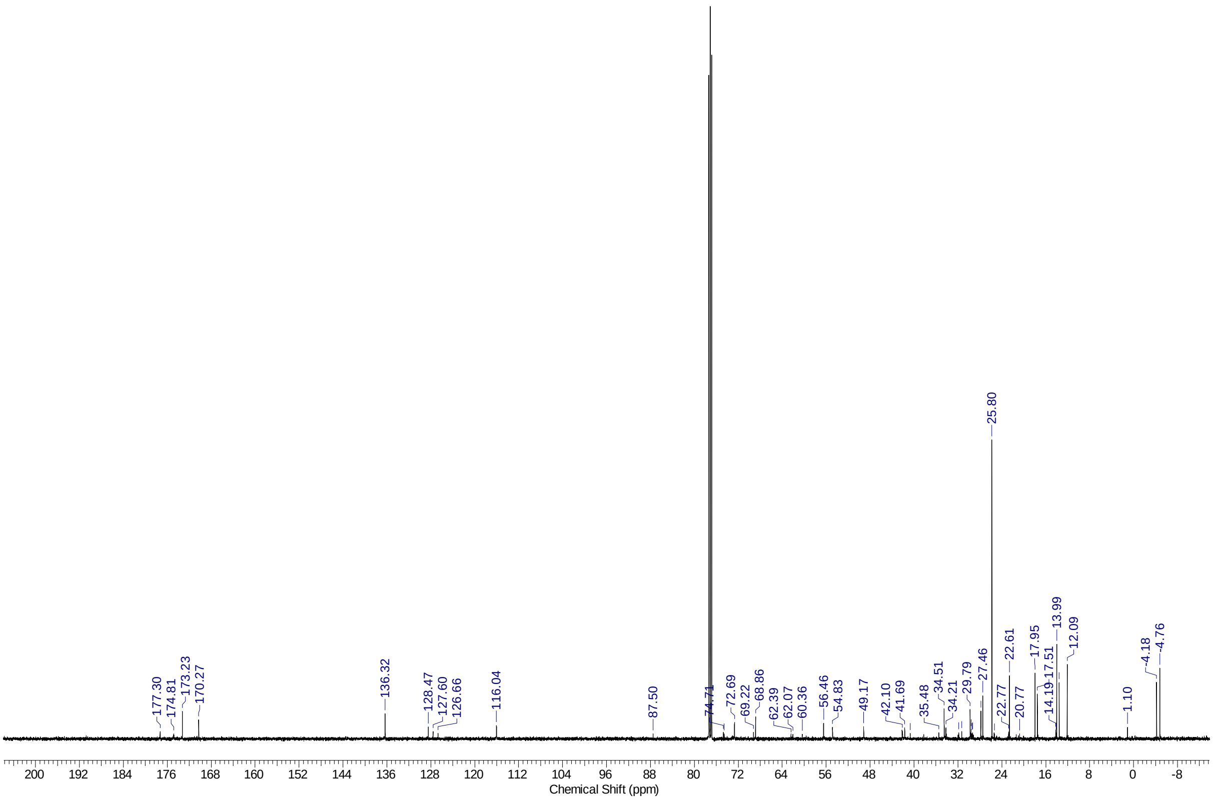

(2*S*,6*R*,9*R*,12*R*,13*S*)-12-((*S*)-*sec*-Butyl)-6-butyl-13-((*tert*-butyldimethylsilyl)oxy)-9-methyl-2-vinyl-1-oxa-5,8,11-triazacyclopentadecane-4,7,10,15-tetraone (9)

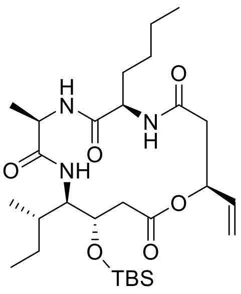

9

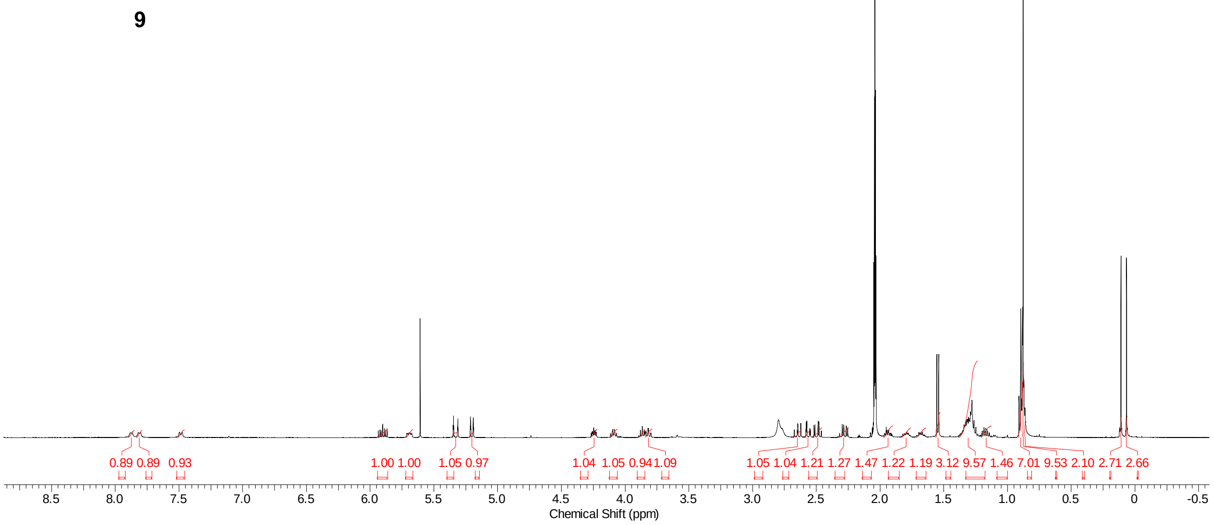

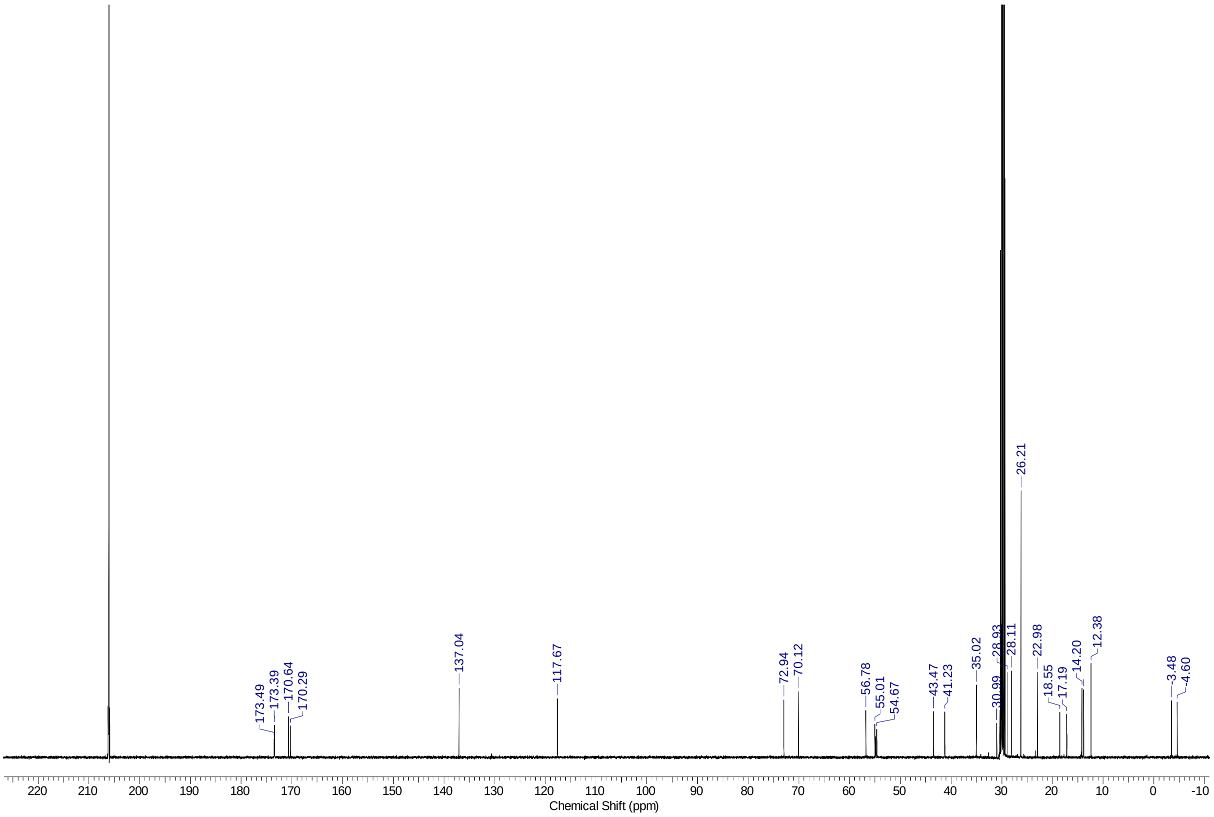

(2*S*,6*R*,9*R*,12*R*,13*S*)-12-((*S*)-*sec*-Butyl)-6-butyl-13-hydroxy-9-methyl-2-vinyl-1-oxa-5,8,11-triazacyclopentadecane-4,7,10,15-tetraone (28)

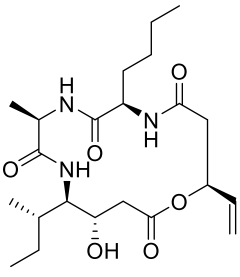

28

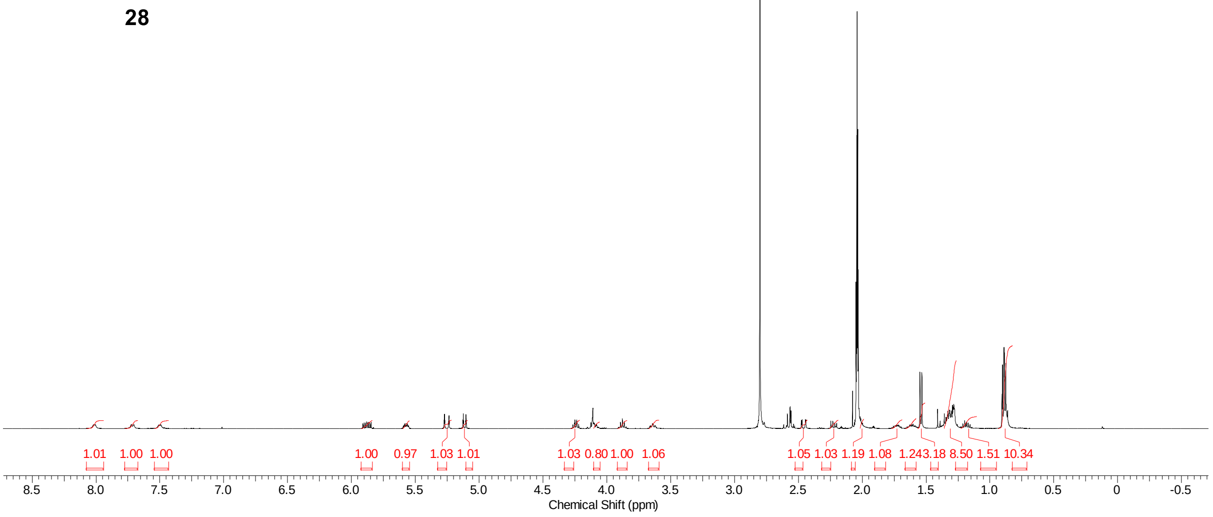

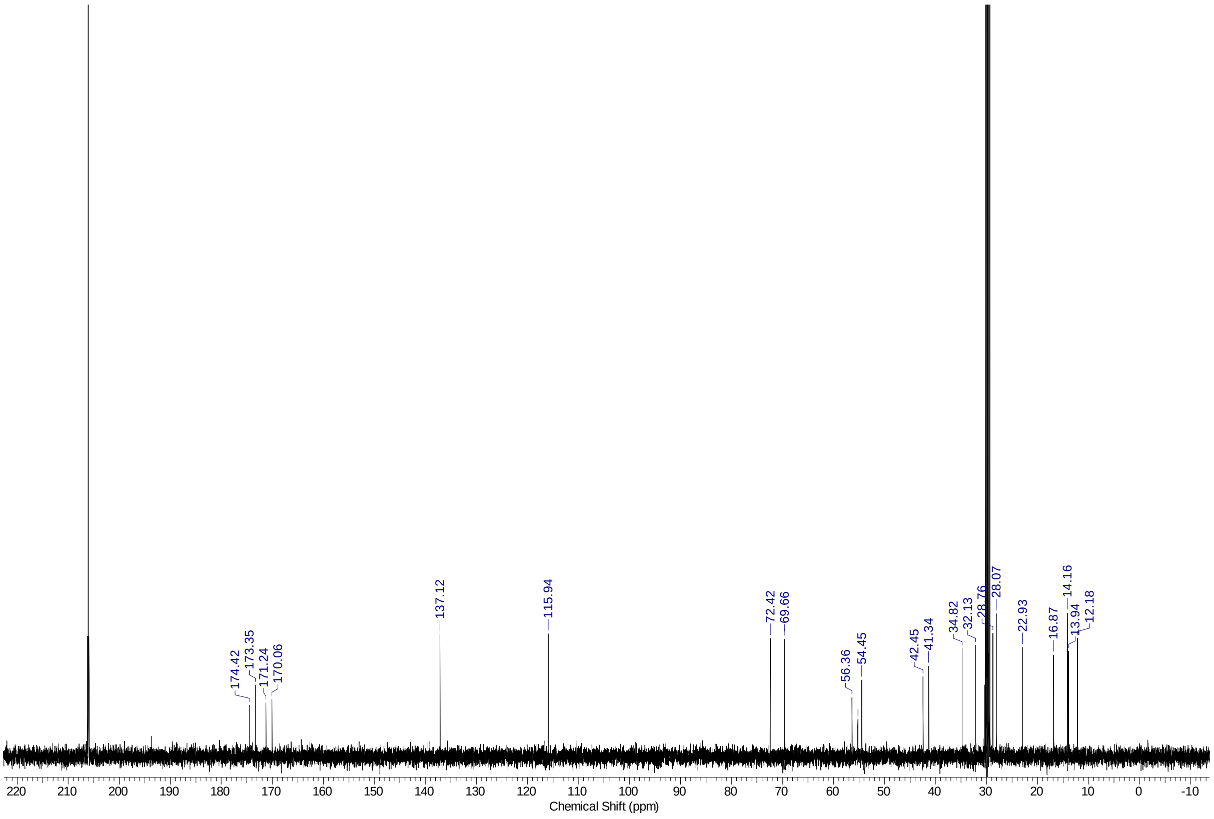

(2*S*,6*R*,9*R*,12*R*,13*S*)-2-((*E*)-4-Bromobut-1-en-1-yl)-12-((*S*)-*sec*-butyl)-6-butyl-13-((*tert*-butyldimethylsilyl)oxy)-9-methyl-1-oxa-5,8,11-triazacyclopentadecane-4,7,10,15-tetraone (30)

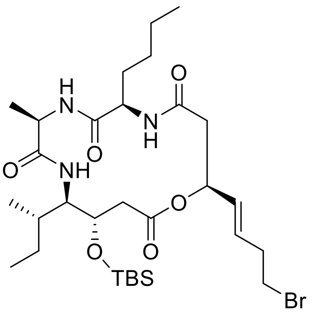

30

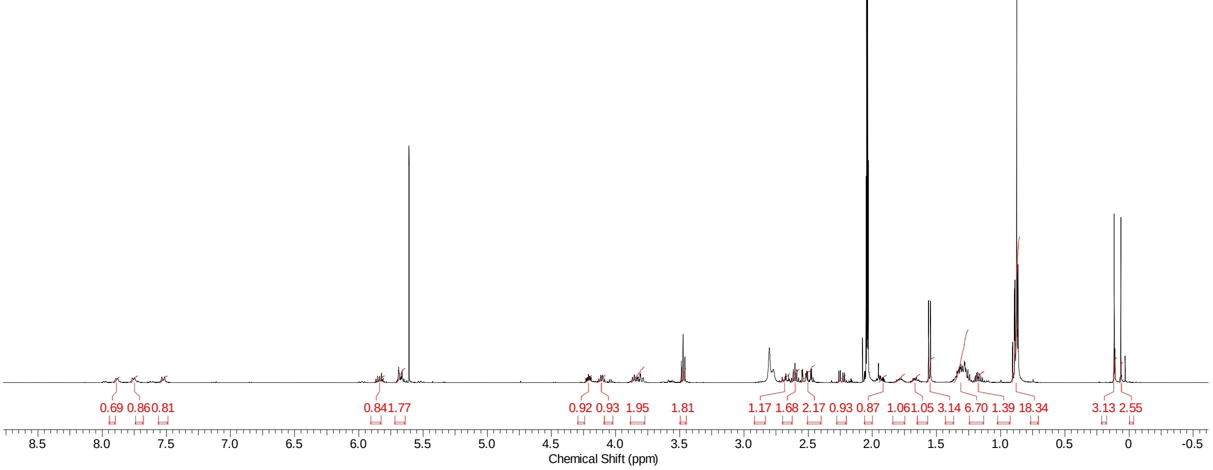

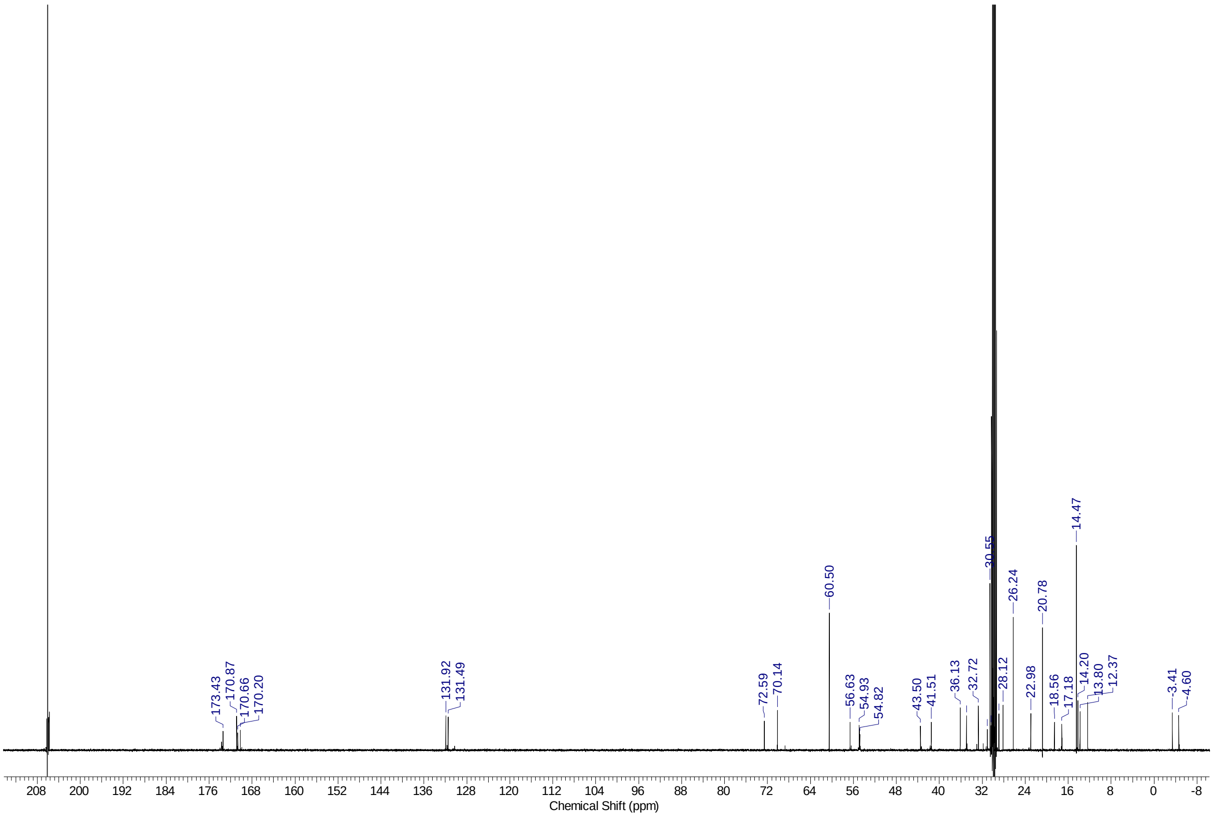

(2*S*,6*R*,9*R*,12*R*,13*S*)-2-((*E*)-4-Bromobut-1-en-1-yl)-12-((*S*)-*sec*-butyl)-6-butyl-13-hydroxy-9-methyl-1-oxa-5,8,11-triazacyclopentadecane-4,7,10,15-tetraone (31)

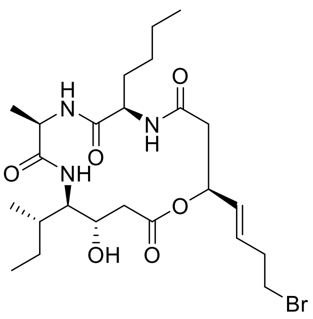

31

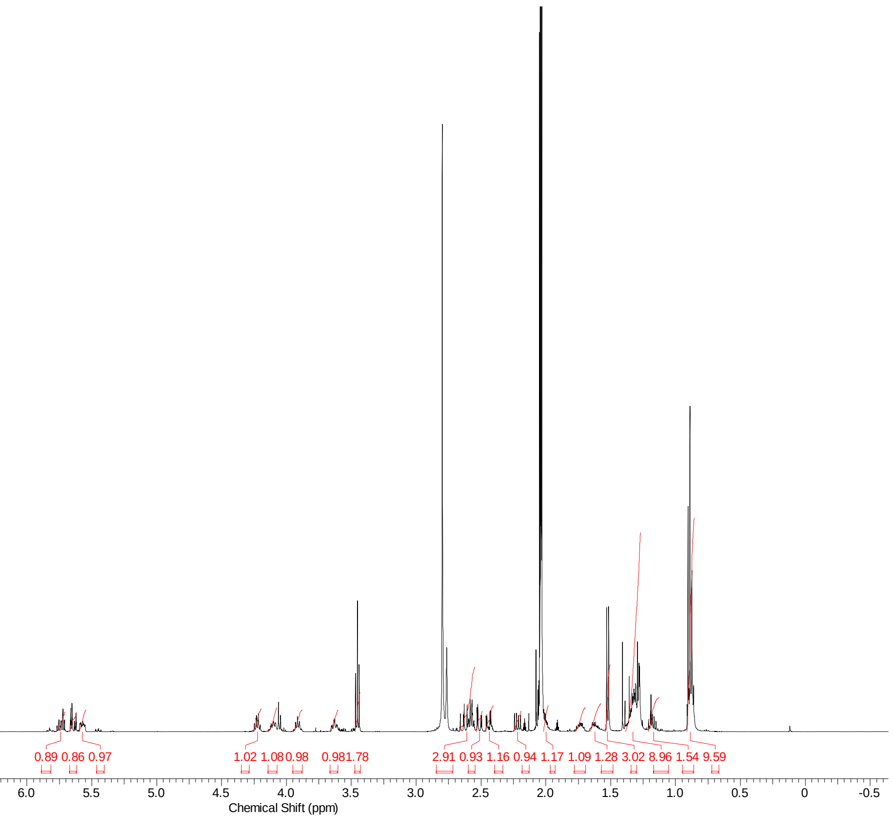

S-((E)-4-((2S,6R,9R,12R,13S)-12-((S)-sec-Butyl)-6-butyl-13-((tert-butyldimethyl-silyl)oxy)-9-methyl-4,7,10,15-tetraoxo-1-oxa-5,8,11-triazacyclopentadecan-2-yl)-but-3-en-1-yl) ethanethioate (32)

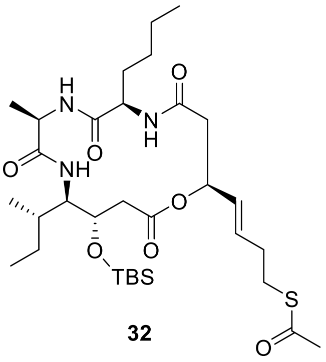

32

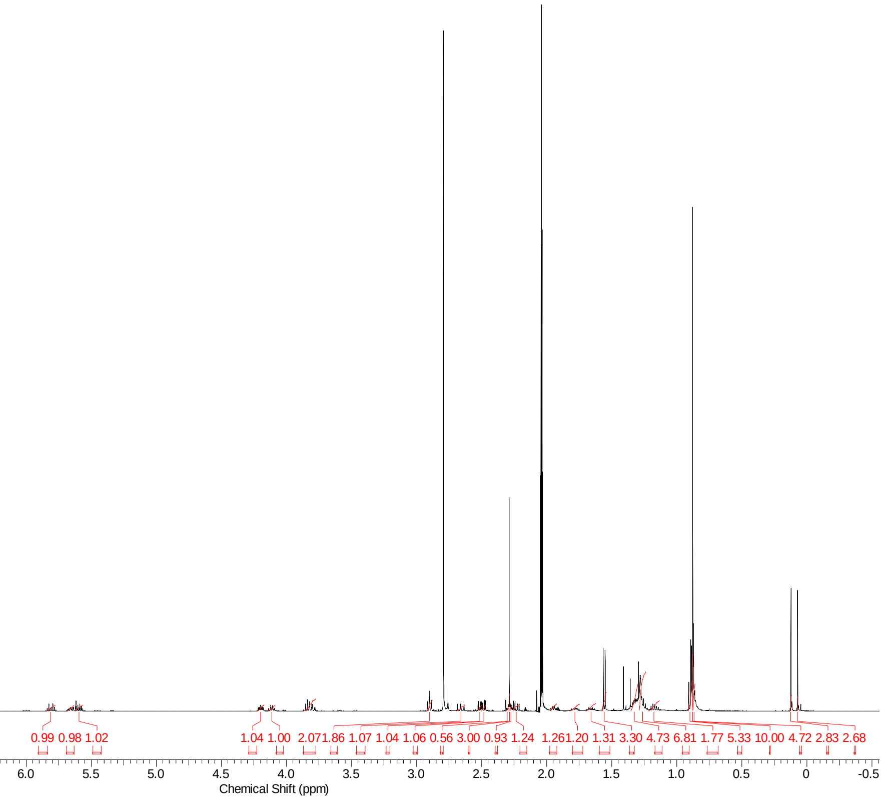

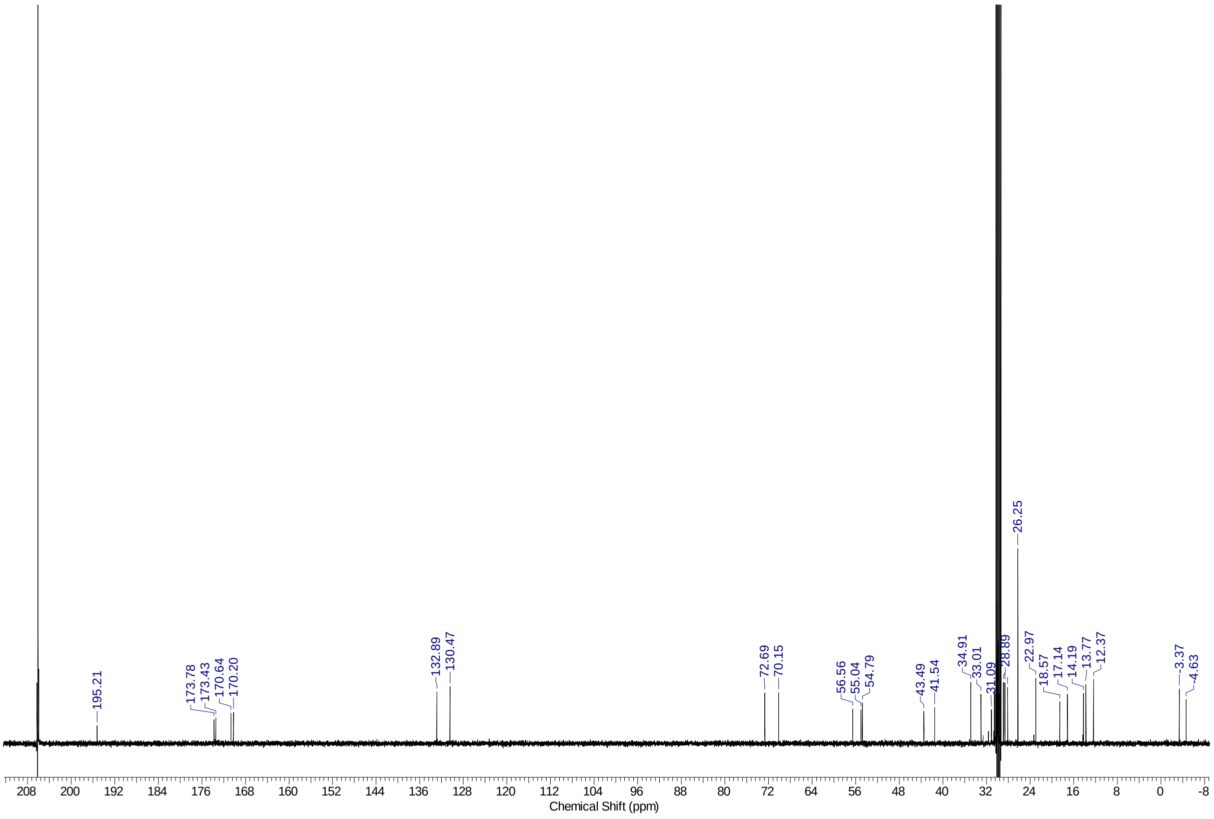

***S*-((*E*)-4-((2*S*,6*R*,9*R*,12*R*,13*S*)-12-((*S*)-*sec*-Butyl)-6-butyl-13-hydroxy-9-methyl-4,7,10,15-tetraoxo-1-oxa-5,8,11-triazacyclopentadecan-2-yl)but-3-en-1-yl) ethane-thioate (33)**

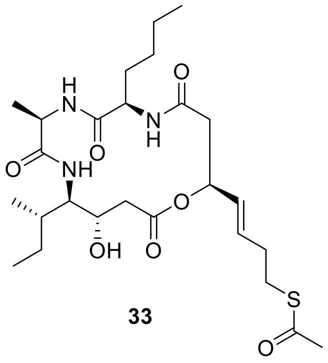

33

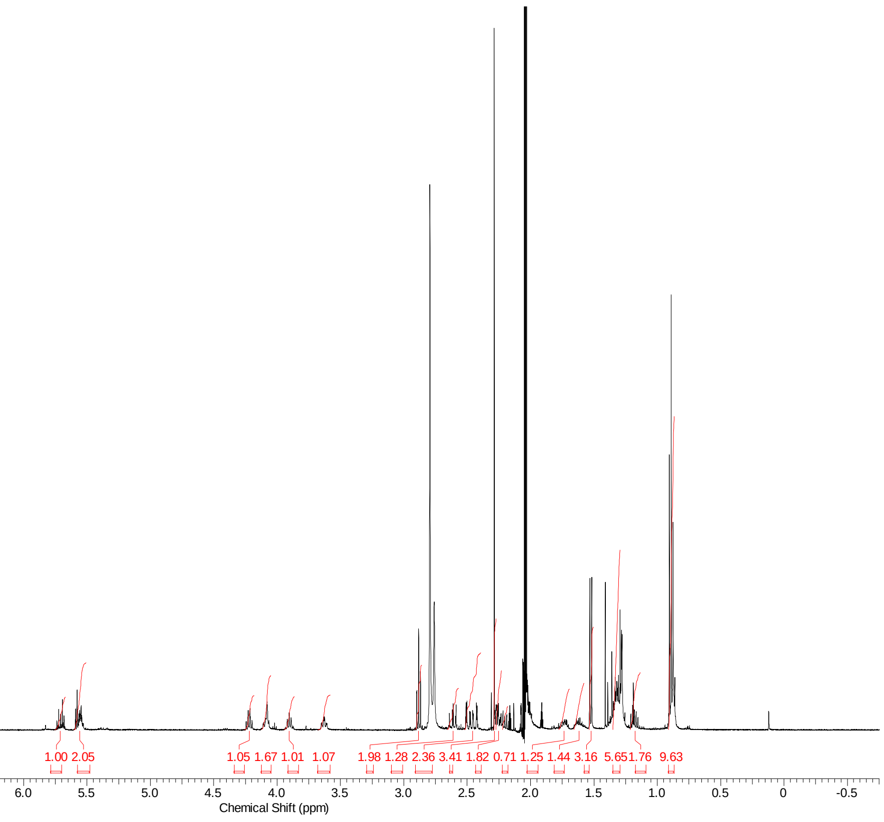

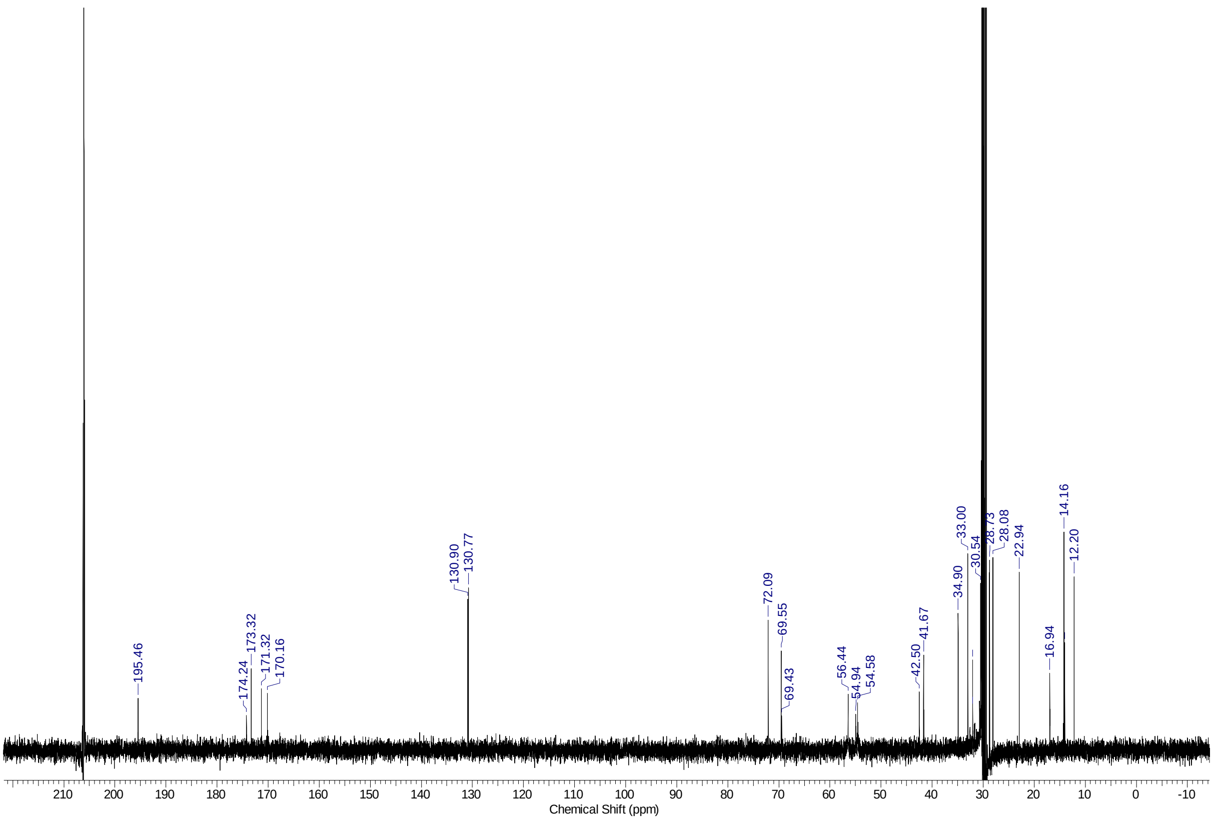

***tert*-Butyl ((*tert*-butoxycarbonyl)oxy)((*E*)-5-((2*S*,6*R*,9*R*,12*R*,13*S*)-12-((*S*)-*sec*-butyl)-6-butyl-13-((*tert*-butyldimethylsilyl)oxy)-9-methyl-4,7,10,15-tetraoxo-1-oxa-5,8,11-triazacyclopentadecan-2-yl)pent-4-enoyl)carbamate (35)**

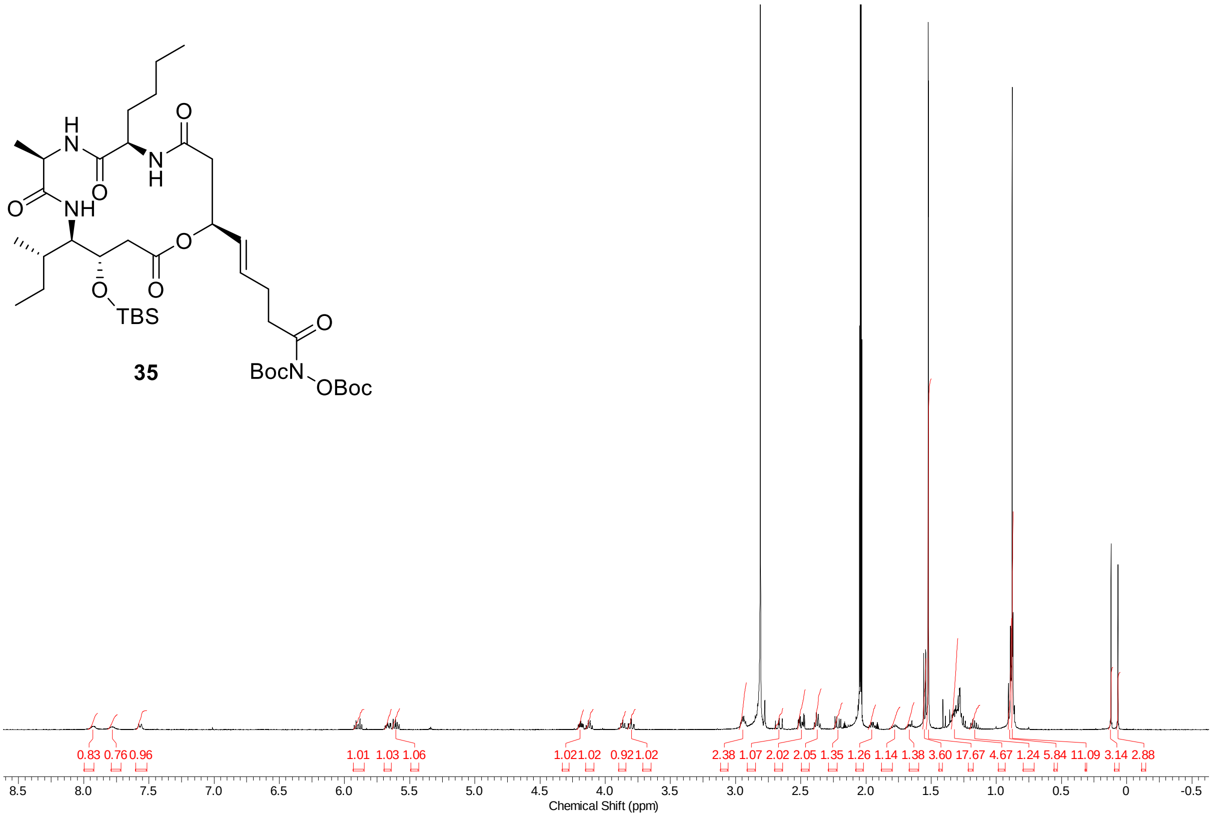

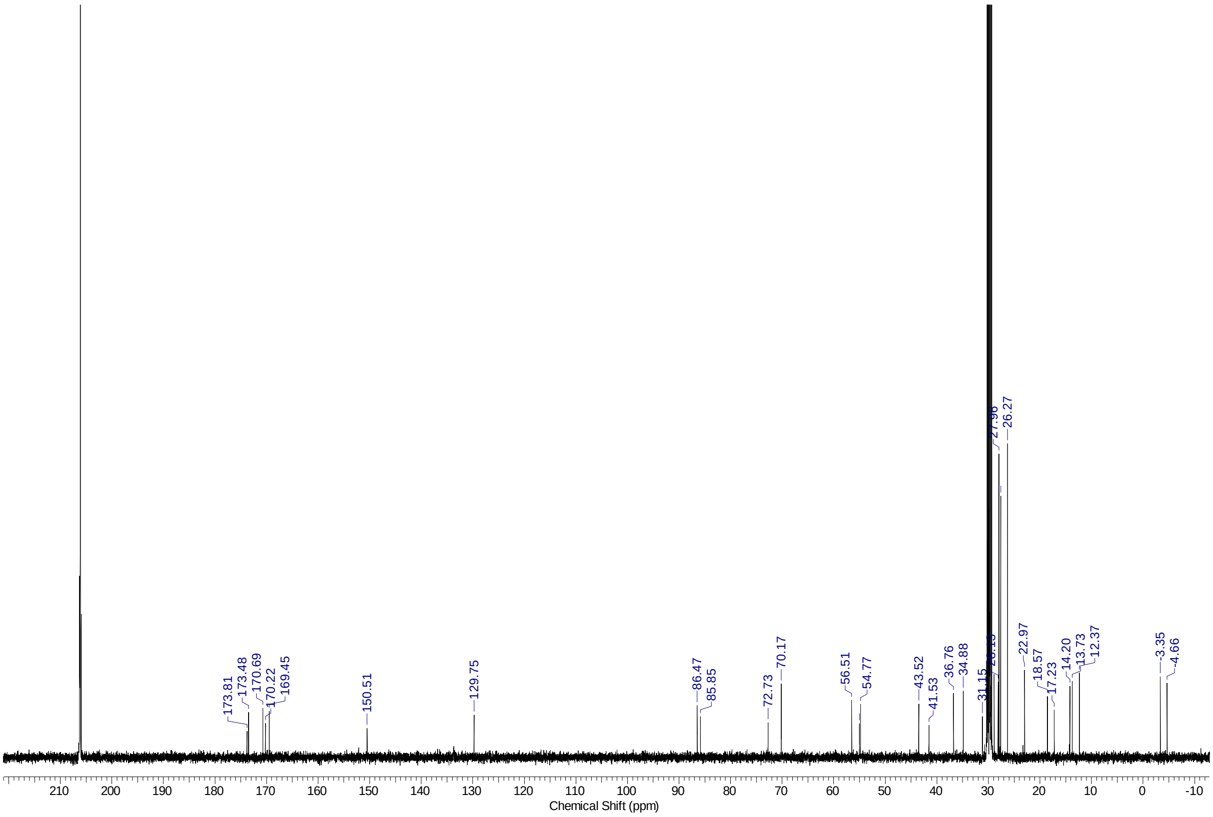

***tert*-Butyl ((*tert*-butoxycarbonyl)oxy)((*E*)-5-((2*S*,6*R*,9*R*,12*R*,13*S*)-12-((*S*)-*sec*-butyl)-6-butyl-13-hydroxy-9-methyl-4,7,10,15-tetraoxo-1-oxa-5,8,11-triazacyclopenta-decan-2-yl)pent-4-enoyl)carbamate (36)**

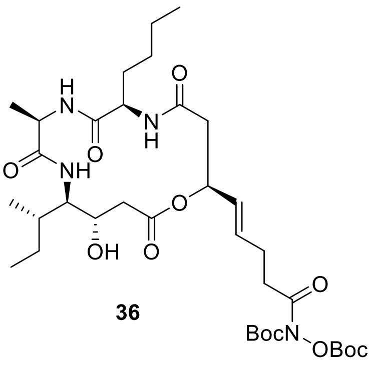

36

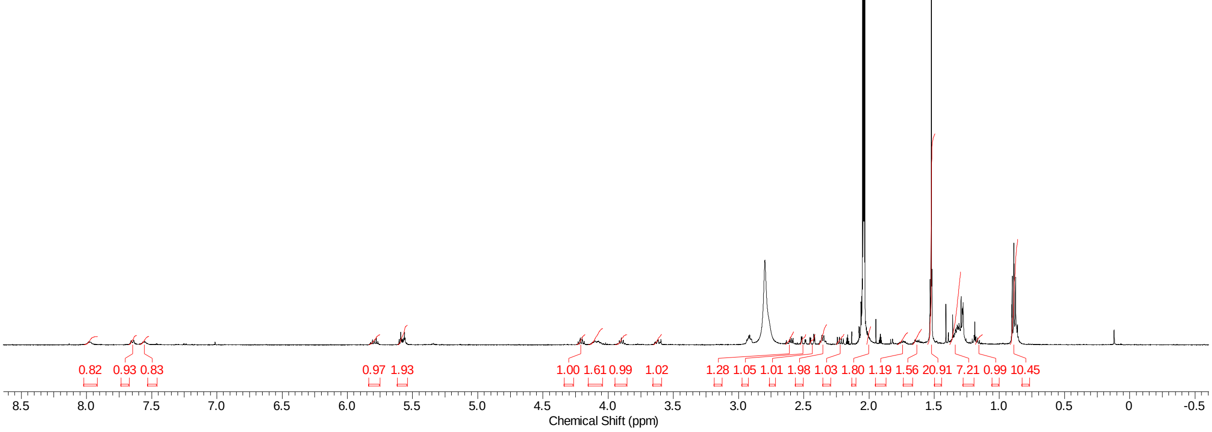

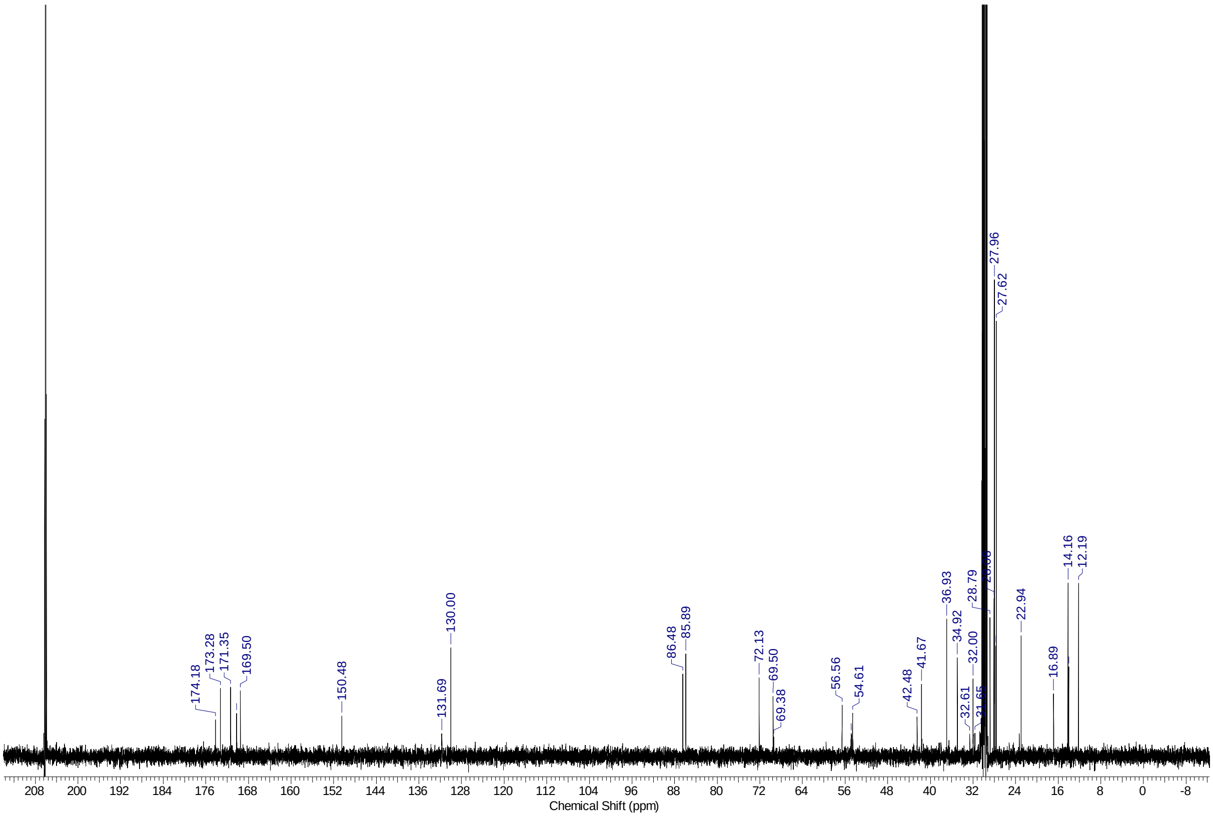

(*E*)-5-((2*S*,6*R*,9*R*,12*R*,13*S*)-12-((*S*)-*sec*-Butyl)-6-butyl-13-hydroxy-9-methyl-4,7,10,15-tetraoxo-1-oxa-5,8,11-triazacyclopentadecan-2-yl)-*N*-hydroxypent-4-enamide (8)

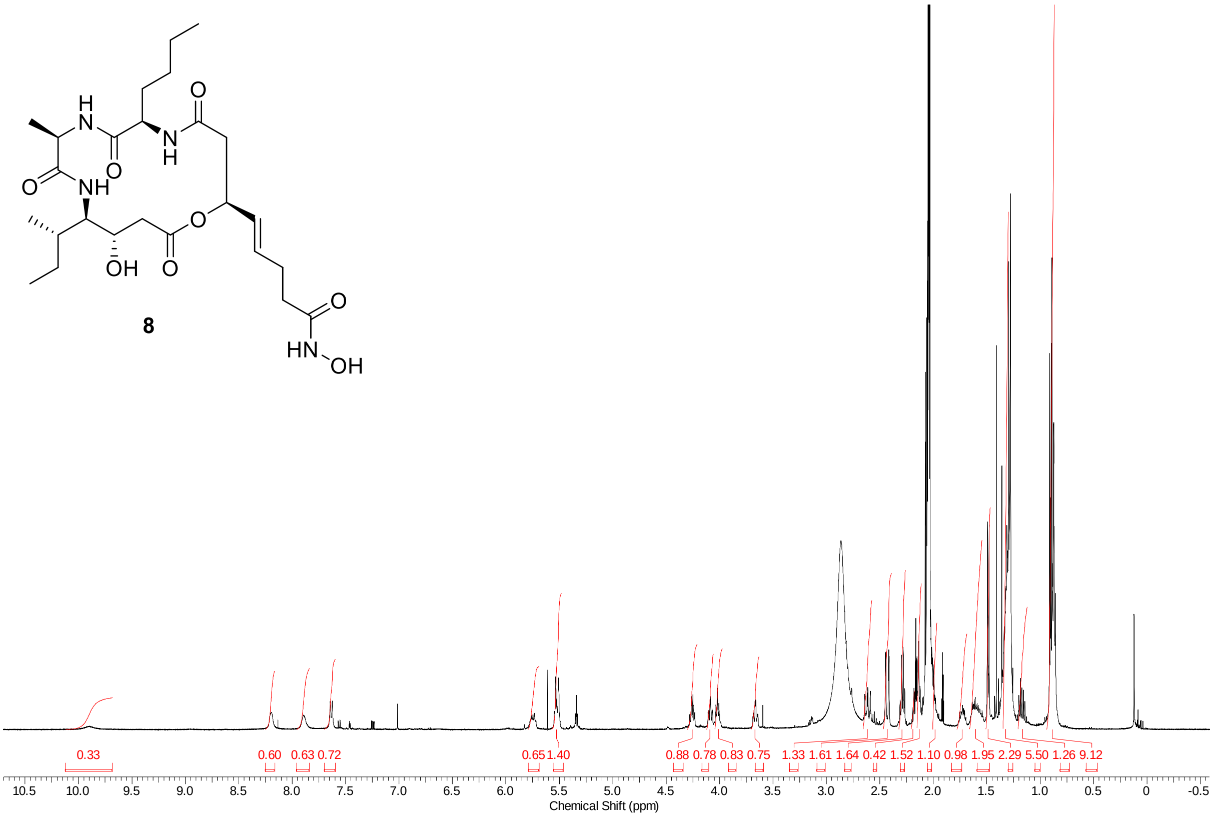

References

[1] B. Bolte, Y. Obadachian. F. Gagosz, *J. Am. Chem. Soc.* **2010**, *132*, 7294-7296.

[2] K. Xu, Y.-H. Wang, V. Khakyzadeh, B. Breit, *Chem. Sci.* **2016**, *7*, 3313-3316.

[3] Z. J. Wang, N. D. Spiccia, W. R. Jackson, A. J. Robinson *J. Pept. Sci*. **2013**, *19*, 470-476.

[4] S. Wang, S. Zhou, J. Wang, Y. Nian, A. Kawashima, H. Moriwaki, J. L. Aceña, V. A. Soloshonok, H. Liu, *J. Org. Chem.* **2015**, *80*, 9817-9830.

[5] Prepared according to a literature known procedure: U. Schmidt, M. Kroner, H. Griesser, *Synthesis* **1989**, 832-835.

[6] Prepared according to a literature known procedure: A. Ďuriš, D. M. Barber, H. J. Sanganee, J. D. Dixon, *Chem. Commun.* **2013**, *49*, 2777- 2779.

[7] M. L. Cooke, K. Xu, B. Breit, *Angew. Chem. Int. Ed.* **2012**, *51*, 10876-10879.

[8] a) J. A. Dale, H. S. Mosher, *J. Am. Chem. Soc.* **1973**, *95*, 512-519; b) J. M. Seco, E. Quinoa, R. Riguera, *Chem. Rev.* **2004**, *104*, 17-117; c) T. R. Hoye, C. S. Jeffrey, F. Shao, *Nature Prot.* **2007**, *2*, 2451-2458.

[9] Prepared according to a literature known procedure: V. Zwick, A. Nurisso, C. Simoes-Pires, S. Bouchet, N. Martinet, A. Lehotzky, J. Ovadi, M. Cuendet, C. Blanquart, P. Bertrand, *Bioorg. & Med. Chem. Lett*. **2016**, *26*, 154-159.

[10] B. Heltweg, J. Trapp, M. Jung, *Methods* **2005**, *36*, 332-337.

[11] M. Marek, S. Kannan, A. T. Hauser, M. Moraes Mourao, S. Caby, V. Cura, D. A. Stolfa, K. Schmidtkunz, J. Lancelot, L. Andrade, J. P. Renaud, G. Oliveira, W. Sippl, M. Jung, J. Cavarelli, R. J. Pierce, C. Romier, *PLoS Pathog* **2013,** *9*, e1003645.

[12] J. Senger, J. Melesina, M. Marek, C. Romier, I. Oehme, O. Witt, W. Sippl, M. Jung, *J. Med. Chem.* **2016**, *59*, 1545-1555.

[13] A.-T. Hauser, J. M. Gajer (née Wagner), M. Jung in *Protein Acetylation: Methods and Protocols* in *Methods Mol. Biol. Vol. 981* (Eds.: S. B. Hake, C. J. Janzen), Springer New York, N. Y., 2013, pp. 211-227.
